# Mental Health Service Use, Suicide Behavior, and Emergency Department Visits Among Rural US Veterans Who Received Video-Enabled Tablets During the COVID-19 Pandemic

**DOI:** 10.1001/jamanetworkopen.2022.6250

**Published:** 2022-04-06

**Authors:** Kritee Gujral, James Van Campen, Josephine Jacobs, Rachel Kimerling, Dan Blonigen, Donna M. Zulman

**Affiliations:** 1 Health Economics Resource Center, VA Palo Alto Health Care System, Menlo Park, California; 2 Center for Innovation to Implementation (Ci2i), VA Palo Alto Health Care System, Menlo Park, California; 3 Division of Primary Care and Population Health, Department of Medicine, Stanford University School of Medicine, Stanford, California; 4 National Center for Post-Traumatic Stress Disorder, VA Palo Alto Health Care System, Menlo Park, California

## Abstract

**Importance:**

Suicide rates are rising disproportionately in rural counties, a concerning pattern as the COVID-19 pandemic has intensified suicide risk factors in these regions and exacerbated barriers to mental health care access. Although telehealth has the potential to improve access to mental health care, telehealth’s effectiveness for suicide-related outcomes remains relatively unknown.

**Objective:**

To evaluate the association between the escalated distribution of the US Department of Veterans Affairs’ (VA’s) video-enabled tablets during the COVID-19 pandemic and rural veterans’ mental health service use and suicide-related outcomes.

**Design, Setting, and Participants:**

This retrospective cohort study included rural veterans who had at least 1 VA mental health care visit in calendar year 2019 and a subcohort of patients identified by the VA as high-risk for suicide. Event studies and difference-in-differences estimation were used to compare monthly mental health service utilization for patients who received VA tablets during COVID-19 with patients who were not issued tablets over 10 months before and after tablet shipment. Statistical analysis was performed from November 2021 to February 2022.

**Exposure:**

Receipt of a video-enabled tablet.

**Main Outcomes and Measures:**

Mental health service utilization outcomes included psychotherapy visits, medication management visits, and comprehensive suicide risk evaluations (CSREs) via video and total visits across all modalities (phone, video, and in-person). We also analyzed likelihood of emergency department (ED) visit, likelihood of suicide-related ED visit, and number of VA’s suicide behavior and overdose reports (SBORs).

**Results:**

The study cohort included 13 180 rural tablet recipients (11 617 [88%] men; 2161 [16%] Black; 301 [2%] Hispanic; 10 644 [80%] White; mean [SD] age, 61.2 [13.4] years) and 458 611 nonrecipients (406 545 [89%] men; 59 875 [13%] Black or African American; 16 778 [4%] Hispanic; 384 630 [83%] White; mean [SD] age, 58.0 [15.8] years). Tablets were associated with increases of 1.8 psychotherapy visits per year (monthly coefficient, 0.15; 95% CI, 0.13-0.17), 3.5 video psychotherapy visits per year (monthly coefficient, 0.29; 95% CI, 0.27-0.31), 0.7 video medication management visits per year (monthly coefficient, 0.06; 95% CI, 0.055-0.062), and 0.02 video CSREs per year (monthly coefficient, 0.002; 95% CI, 0.002-0.002). Tablets were associated with an overall 20% reduction in the likelihood of an ED visit (proportion change, −0.012; 95% CI, −0.014 to −0.010), a 36% reduction in the likelihood of suicide-related ED visit (proportion change, −0.0017; 95% CI, −0.0023 to −0.0013), and a 22% reduction in the likelihood of suicide behavior as indicated by SBORs (monthly coefficient, −0.0011; 95% CI, −0.0016 to −0.0005). These associations persisted for the subcohort of rural veterans the VA identifies as high-risk for suicide.

**Conclusions and Relevance:**

This cohort study of rural US veterans with a history of mental health care use found that receipt of a video-enabled tablet was associated with increased use of mental health care via video, increased psychotherapy visits (across all modalities), and reduced suicide behavior and ED visits. These findings suggest that the VA and other health systems should consider leveraging video-enabled tablets for improving access to mental health care via telehealth and for preventing suicides among rural residents.

## Key Points

**Question:**

Was the US Veterans Affairs initiative to distribute video-enabled tablets during COVID-19 associated with mental health care access, suicide behavior, or emergency department (ED) visits among rural veterans?

**Findings:**

In this retrospective cohort study of 471 791 rural US veterans with a history of mental health care use, receipt of a video-enabled tablet was associated with increased use of mental health care via video, increased psychotherapy visits (across all modalities), and reduced suicide behavior and ED visits.

**Meaning:**

These findings suggest that video-enabled tablets may provide access to critical services for rural patients with mental health needs and reduce instances of suicide behavior and ED visits among them.

## Teaser

This cohort study evaluates the association between the distribution of the US Veterans Affairs’ video-enabled tablets during the COVID-19 pandemic and rural veterans’ mental health service use and suicide-related outcomes.

## Introduction

US suicide rates are at their highest since World War II.^1^ The US veterans’ suicide rate is 1.5 times that of nonveterans^2,3^ and veterans in rural (vs urban) areas are more likely to die by suicide. Suicide is disproportionately affecting rural (vs urban) residents (17.32 vs 11.92 per 100 000 people),^4,5,6,7^ partly because of challenging demographic trends such as growing unemployment and lack of health care resources in rural areas, which have been exacerbated by the COVID-19 pandemic.^5,6,8,9,10^ The pandemic has also intensified suicide risk factors^8,11^—social isolation, intimate partner violence, and firearm access^8,12^—which disproportionately affect rural residents. Reduced interaction with routine health care during the pandemic has reduced opportunities to screen and treat rural residents for suicide risk.^8,13^ Experts have recommended that for rural patients at risk for suicide, ensuring continued access to mental health care via telehealth is crucial.^8^

The US Department of Veterans Affairs (VA) has been leading efforts to expand telehealth and to improve access to care for veterans residing in rural areas.^5,14^ The VA’s substantial efforts to improve access for rural veterans during the years 2007 to 2012 likely helped reduce the rural-urban disparity in psychotherapy access, a first-line treatment for most mental health conditions, including suicide risk.^5,14,15^ Nevertheless, the rural-urban disparity persists as rural veterans continually face challenges related to availability, accessibility, and acceptability of psychotherapy services.^14^ Although telehealth can improve access to mental health care,^16^ there is insufficient evidence regarding rural veterans’ engagement via virtual modalities^14^ and about telehealth’s effectiveness for suicide prevention.^17,18,19^

In 2016, the VA’s Office of Rural Health and Office of Connected Care began distributing video-enabled tablets to veterans with access barriers to facilitate their participation in home-based telehealth.^20,21^ The VA’s tablet distribution efforts intensified during the COVID-19 pandemic. As of September 1, 2021, there were 106 451 tablets in circulation, 93% of which were issued during the pandemic, and approximately one-third of those were issued to veterans living in rural areas. The VA-issued tablets present an opportunity to pilot a scenario in which rural veterans face 1 less barrier to accessing home-based telehealth: smart device ownership. To inform telehealth-related policies, especially pertaining to suicide prevention among rural veterans, we evaluated associations between the VA’s video-enabled tablets issued during COVID-19 and frequency of mental health service use, suicide-related behavior, and emergency department (ED) visits among rural veterans with indicated mental health care needs and within a subcohort VA identified as high-risk for suicide.

## Methods

### Study Cohort

This cohort study included all rural patients who had at least 1 VA mental health care visit in the year 2019 (eFigure 1 in the Supplement). Data on VA patients and visits were obtained from the VA’s Corporate Data Warehouse (CDW), a VA electronic health records repository. Mental health care visits were identified using VA Managerial Cost Accounting stop codes used for characterizing outpatient visits.^22^ Rurality was defined as Rural-Urban Commuting Areas codes other than 1 or 1.1.^23^ Veterans were classified as tablet recipients if they received tablets between March 16, 2020, and April 30, 2021. Veterans who received VA tablets outside this period or received VA smartphones were excluded to isolate associations with VA tablets issued during COVID-19. For all veterans, data were obtained 10 months before the baseline month. Veterans were followed until June 30, 2021; we attempted to observe data 10 months posttablet for most veterans. Our subcohort included rural veterans considered high-risk for suicide using VA’s predictive model that analyzes veterans’ health record data.^24^

This study was designated by the VA’s Office of Rural Health as nonresearch quality improvement and was exempted from review by the Stanford institutional review board, and a waiver of informed patient consent was granted because the study used retrospective observational electronic health records data and data were reported in an aggregate manner in which patients were not identifiable. This study followed the Strengthening the Reporting of Observational Studies in Epidemiology (STROBE) reporting guideline.

### Intervention

Veterans were eligible to receive the VA’s video-enabled tablets with data plans if they did not own a device with broadband or cellular internet service, had an access barrier such as living far from the VA or another transportation challenge, and were able to physically and cognitively operate a tablet. Clinicians initiated tablet consultation meetings for patients they thought would qualify. If veterans were considered eligible, tablets were ordered to be mailed to those veterans. Data regarding tablet recipients and tablet shipment dates were obtained from the VA’s Denver Acquisitions and Logistics Center.^20,21^

### Outcomes

Mental health utilization outcomes included psychotherapy visits, medication management visits and comprehensive suicide risk evaluations (CSREs) via video and total visits across all modalities (phone, video, and in-person). Other outcomes were likelihood of ED visit, likelihood of suicide-related ED visit, and number of VA’s suicide behavior and overdose reports (SBORs).

Psychotherapy visits were defined using *Current Procedural Terminology* codes, and medication management visits were defined as encounters with psychiatrists or other mental health clinicians qualified to prescribe medications (eTable 2, eTable 3, eTable 4, eTable 5, and eTable 6 in the Supplement). CSREs are standardized VA templates used for assessing suicide risk for high-risk veterans and include in-depth questions related to suicidal ideation, history of attempts, warning signs, risk factors, protective factors and reasons for living.^25,26^ SBORs are standardized VA templates that may be completed by the first clinical staff member who learns of a veteran’s suicide event or overdose.^19,26,27^

Visit data were obtained from the VA’s CDW. Video visits were identified using VA stop codes of 179, 648, and 679, which indicated clinic-to-home and clinic-to-other/non-VA settings video telehealth care.^22^ Data on CSREs and SBORs were obtained from the VA’s Program Evaluation Resource Center (PERC).^28^ Data on ED visits were obtained from the VA’s CDW. Suicide-related ED visits were identified using PERC’s list of suicide-related *International Statistical Classification of Diseases and Related Health Problems, Tenth Revision (ICD-10)* diagnosis codes (eTable 7 and eTable 8 in the Supplement). In sensitivity analyses, we used alternate suicide-related *ICD-10* codes from prior studies.^29,30^

### Covariates

All models were adjusted for veterans’ age, sex, race, number of physical and mental health chronic conditions (eTable 9 in the Supplement),^22,31,32,33^ indicators for diagnoses of substance use disorder (SUD), posttraumatic stress disorder (PTSD) and depression, VA-estimated 1-year probability of hospitalization or death called VA Care Assessment Needs (CAN) score,^34^ VA priority-based enrollment categories (based on veterans’ service-connected disabilities and other factors^22,35^), marital status, homelessness, high suicide risk indicator, and monthly COVID-19 cases in patients’ counties. To account for any remaining fixed difference between tablet recipients and nonrecipients, we included an indicator for being a tablet recipient. We included fixed effects for patients’ closest secondary care facility to control for any time-invariant facility characteristics. We included month fixed effects to control for shocks or events of each month. Month fixed effects, among other things, controlled for the pandemic in any given month.

Age, sex, race and ethnicity, diagnoses, CAN score, VA priority-based and marital status were obtained from the VA’s CDW electronic health record data. We adjusted for race and ethnicity in our models because these factors may influence outcomes. Distances to patients’ closest facilities were obtained from the VA’s Planning Systems Support Group. Homelessness was defined using outpatient stop codes indicating use of the VA’s homeless services and diagnosis codes.^22^ Data on county-level COVID-19 cases were obtained from *The New York Times*.^36^

### Statistical Analysis

We used event studies^37,38,39^ and difference-in-differences (DiD) estimation to compare outcomes for veterans who received VA-issued tablets and veterans who never received tablets, before and after tablet shipment. DiD estimation leverages a comparison group not exposed to tablets to adjust for temporal variation in outcomes that was not due to treatment exposure.^40^ DiD assumes treatment and control groups would have exhibited similar or parallel trends in the absence of treatment (tablets in our case). Recent developments in DiD methods show that with variation in treatment timing (ie, differential timing of tablet shipment across veterans), event studies improve on the usual DiD estimator.^41,42,43^ Event studies provide DiD estimates for each period (each month in our case) prior to and after treatment (ie, pretablet and posttablet shipment).^41,43^ Pretreatment (or pretablet) model-adjusted differences between the treatment group (vs control) are visually assessed for significance or trending that could obscure or mask true differences in the posttreatment period.^41,43,44^ Pretreatment trends provide important context for interpreting posttreatment differences.^44^ The absence of pretreatment associations or trends, followed by abruptly different and significant posttreatment associations strengthen attributability of associations to the treatment (tablets).^41,43,44^ In this study, the intervention variable for event studies was month-relative-to-tablet-shipment, where shipment month was relative month 0. We excluded relative months −1 and 0 to avoid attributing tablet assignment- and setup-related visits to tablet-associated outcomes, making relative month −2 (ie, 2 months prior to tablet shipment) the baseline month. For estimating usual DiD estimates, the intervention variable was the interaction between indicators for tablet recipient and posttablet (method details in eAppendix 1 in the Supplement). In sensitivity analyses, we conducted the same analyses with a subcohort of matched veterans, using 1:1 nearest neighbor matching.^45,46^

The statistical significance threshold was *P* < .05, and all testing was 2-sided. All statistical analyses were conducted in Stata version 15.1 (StataCorp) from November 2021 to February 2022.

## Results

Table 1 presents unadjusted baseline characteristics for 13 180 veterans living in rural areas who received tablets (11 617 [88%] men; 2161 [16%] Black or African American; 301 [2%] Hispanic; 10 644 [80%] White; mean [SD] age, 61.2 [13.4] years) and 458 611 veterans who did not receive tablets (406 545 [89%] men; 59 875 [13%] Black or African American; 16 778 [4%] Hispanic; 384 630 [83%] White; mean [SD] age, 58.0 [15.8] years). The follow-up period ranged from 1 to 10 months with a mean of 8.7 months and a median of 10 months.

**Table 1.** Unadjusted Baseline Characteristics for Tablet Recipients and Nonrecipients

Characteristic	Participants, No. (%)		*P* value^a^
	Rural tablet nonrecipients (n = 458 611)	Rural tablet recipients (n = 13 180)	
Outcomes			
Any psychotherapy visit	56 775 (12)	2534 (19)	<.001
Any video psychotherapy visit	640 (0.1)	673 (5)	<.001
Any video medication management visit	378 (0.1)	317 (2)	<.001
Any video visit for a Comprehensive Suicide Risk Evaluation	14 (0.003)	17 (0.013)	<.001
Any ED visit	25 050 (5)	775 (6)	<.001
Any suicide-related ED visit	850 (0.2)	68 (0.5)	<.001
Any VA suicide behavior or overdose report	576 (0.1)	64 (0.5)	<.001
Covariates			
Sex			
Male	406 545 (89)	11 617 (88)	.07
Female	52 066 (11)	1563 (12)	.07
Age, mean (SD)	58.0 (15.8)	61.2 (13.4)	<.001
Homeless	9005 (2)	924 (7)	<.001
Distance to closest VA primary care site, mean	25.5	23.8	<.001
No. of physical chronic conditions in 2019, mean	4.5	5.5	<.001
No. of mental chronic conditions, mean	1.8	2.3	<.001
Diagnosed with substance use disorder in 2019	76 779 (17)	3615 (27)	<.001
Diagnosed with PTSD in 2019	208 359 (45)	6687 (51)	<.001
Diagnosed with depression in 2019	222 653 (49)	7476 (57)	<.001
VA Care Assessment Needs score	0.1	0.2	<.001
VA classification of high-risk for suicide^b^			
Never classified as high-risk for suicide	443 106 (97)	12 023 (91)	<.001
Classified as high-risk for suicide (but < top 1% of risk)	14 551 (3)	1009 (8)	<.001
Classified as top 1% of suicide risk	954 (0.2)	148 (1)	<.001
VA priority-based enrollment categories			
1	270 364 (59)	7345 (56)	<.001
2	31 856 (7)	810 (6)	<.001
3	40 009 (9)	1138 (9)	.72
4	10 623 (2)	627 (5)	<.001
5	66 682 (15)	2639 (20)	<.001
6	8459 (2)	106 (1)	<.001
7	4504 (1)	90 (1)	<.001
8	26 114 (6)	425 (3)	<.001
Ethnicity			
Hispanic	16 778 (4)	301 (2)	<.001
Not Hispanic	435 235 (95)	12 705 (97)	
Unknown	6598 (1)	174 (1)	.26
Race			
American Indian or Alaska Native	8208 (2)	245 (2)	.56
Asian	2107 (0.5)	39 (0.3)	.006
Black or African American	59 875 (13)	2161 (16)	<.001
Native Hawaiian or other Pacific Islander	3791 (1)	91 (1)	.09
White	384 630 (83)	10 644 (80)	<.001
Marital status			
Divorced	108 606 (24)	3837 (29)	<.001
Married	253 143 (55)	6091 (46)	<.001
Separated	17 750 (4)	685 (5)	<.001
Widowed	15 296 (3)	565 (4)	<.001
Unknown	4539 (1)	65 (0.5)	<.001

Abbreviations: ED, emergency department; PTSD, posttraumatic stress disorder; VA, US Department of Veterans Affairs.

^a^ Differences in proportions of dichotomous variables were tested using the Pearson χ^2^ test. Differences in means of continuous variables were tested using the 2-sample *t* test.

^b^ The VA classifies veterans as high-risk for suicide using the VA’s model that analyzes existing data from veterans’ health records to identify statistically elevated risk for suicide, hospitalization, illness, or other adverse outcomes.

Table 1 shows that recipients and nonrecipients were similar with respect to their sex distribution, distance to closest primary care site, and priority-based enrollment categories. Compared with nonrecipients, tablet recipients had higher mean (SD) age (61.2 [13.4] years vs 58.0 [15.8] years; *P* < .001), were more likely to be homeless (7% [n = 924] vs 2% [n = 9005]; *P* < .001), had higher mean (SD) physical chronic conditions (5.5 [3.3] vs 4.5 [3.1]; *P* < .001) and mental chronic conditions (2.3 [1.4]vs 1.8 [1.2]; *P* < .001), were more likely to be diagnosed with SUD (27% [n = 3615] vs 17% [n = 76 779]; *P* < .001), PTSD (51% [n = 6687] vs 45% [n = 208 359]; *P* < .001), or depression (57% [n = 7476] vs 49% [n = 222 653]; *P* < .001), had higher clinical risk based on CAN score (0.2 [0.2] vs 0.1 [0.1]; *P* < .001), and were more likely to be classified as high-risk for suicide (8% [n = 1009] vs 3% [n = 14 551]; *P* < .001). Tablet recipients were more likely to be Black or African American (16% [n = 2161] vs 13% [n = 59 875]; *P* < .001) and were less likely to be married (46% [n = 6091] vs 55% [n = 253 143]; *P* < .001).

**Figure 1.**
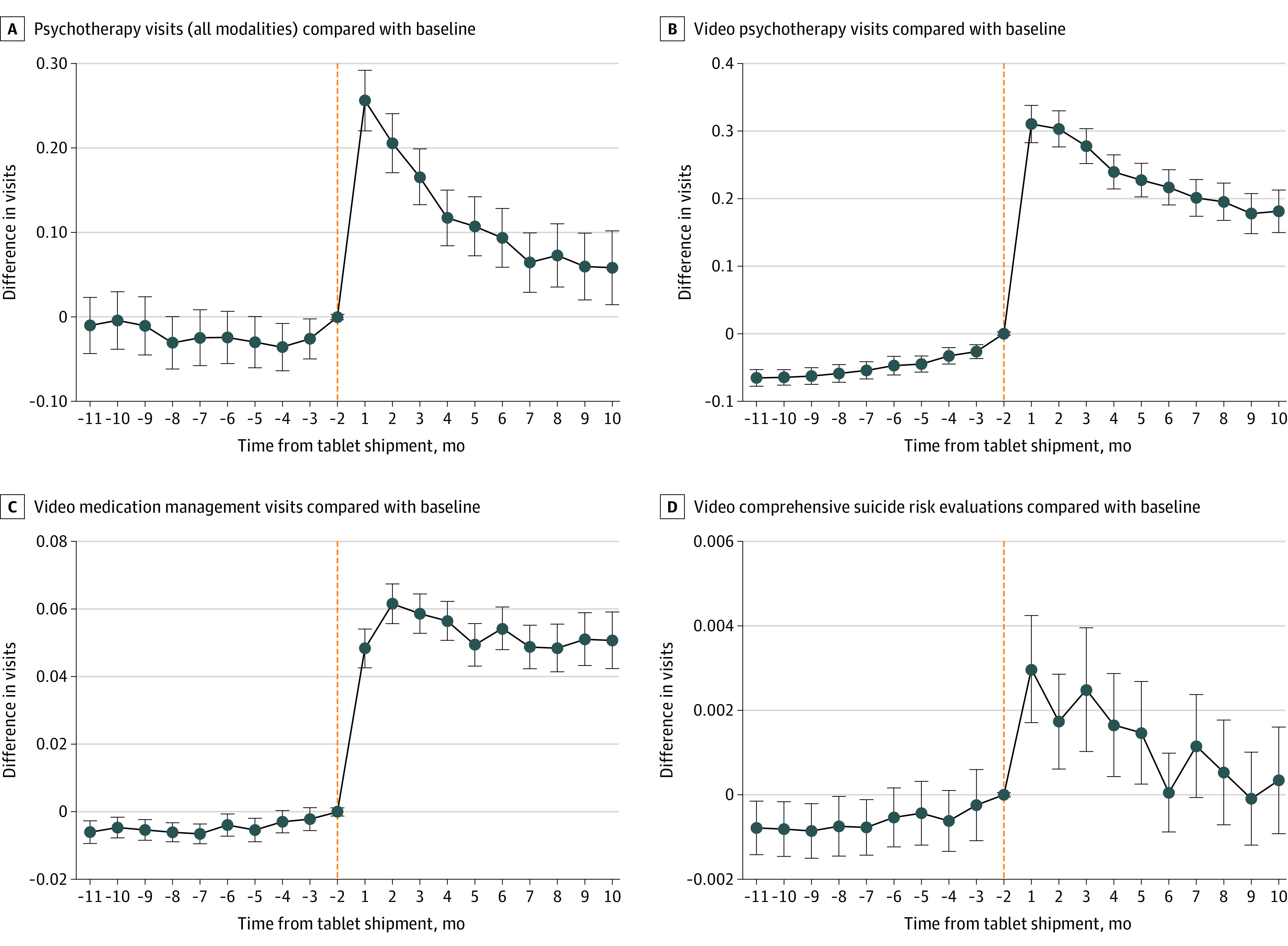
Event Study Estimates of Adjusted Differences in Mental Health Service Use for Tablet Recipients vs Recipients’ Baseline and Nonrecipients Month −1 and month 0 were excluded because treatment assignment (ie, tablet assignment) likely occurred in these months and we did not want to attribute tablet assignment-related visits to tablet-associated outcomes. All models adjusted for veterans' sociodemographic and clinical characteristics, county-level COVID-19 cases, and the fixed effects of being a tablet recipient, of each month, and of each facility.

**Figure 2.**
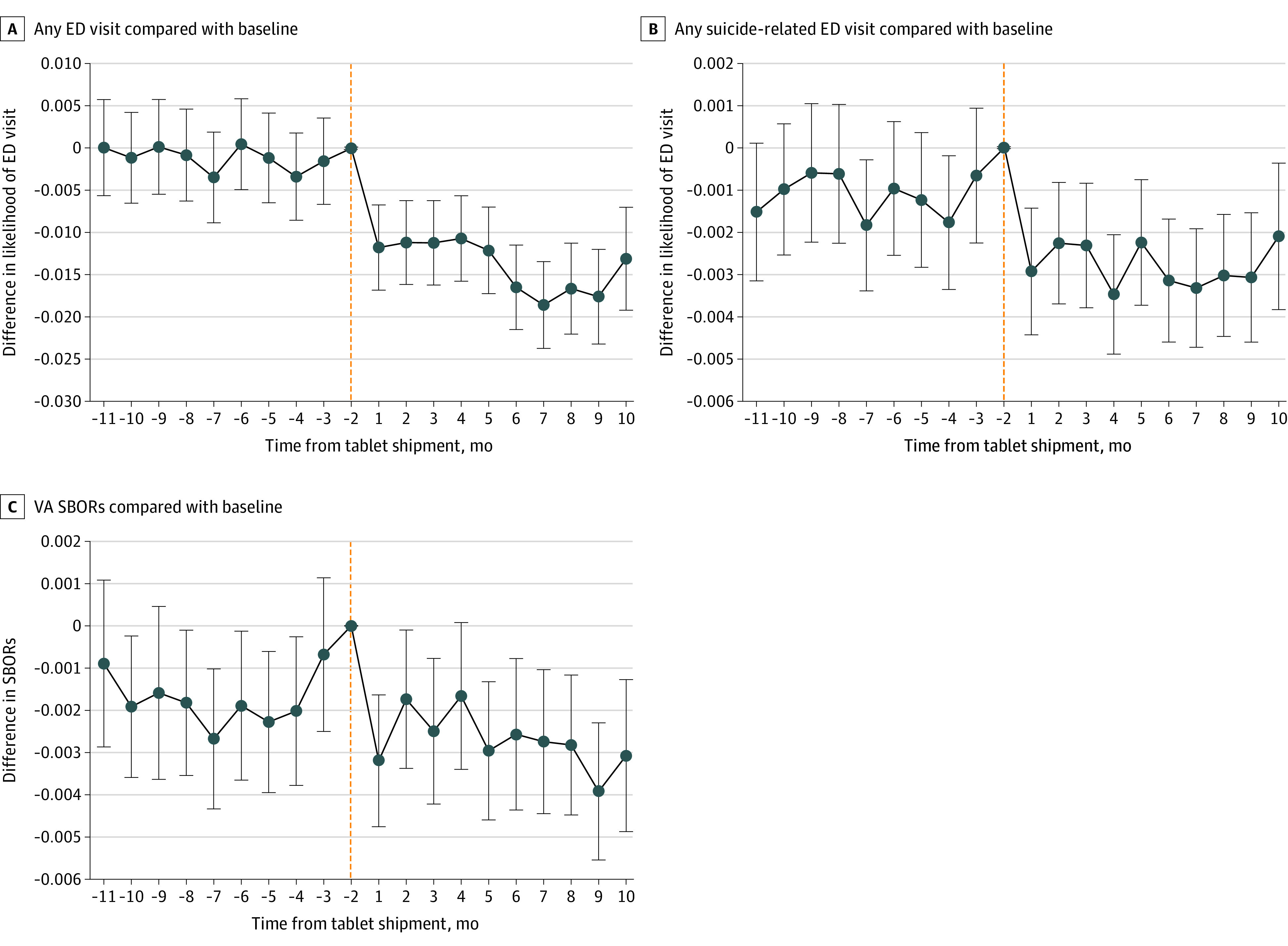
Event Study Estimates of Adjusted Differences in Emergency Department (ED) Visits and Suicide Behavior for Tablet Recipients vs Recipients’ Baseline and Nonrecipients Month −1 and month 0 were excluded because treatment assignment (ie, tablet assignment) likely occurred in these months and we did not want to attribute tablet assignment-related visits to tablet-associated outcomes. All models adjusted for veterans' sociodemographic and clinical characteristics, county-level COVID-19 cases, and fixed effect of being a tablet recipient, of each month, and of each facility. SBOR indicates suicide behavior and overdose report; VA indicates US Department of Veterans Affairs.

Figure 1 and Figure 2 present model-adjusted mean differences in frequency of visits between tablet recipients, compared with baseline or nonrecipients. Figures 1 and 2 broadly show that after adjusting for covariates, there were few significant differences in outcomes between recipients and nonrecipients and no meaningful trending in these differences in the months prior to tablet shipment, followed by significant associations posttablet shipment. Calendar-time graphs demonstrated that the pandemic did not differentially affect tablet recipients and nonrecipients such that nonrecipients provided a strong counterfactual trend for recipients even during the COVID-19 period (eFigure3 in Supplement). We did not find significant associations between tablets and visits for medication management visits or CSREs across all modalities (eFigure 2 in the Supplement). For video psychotherapy visits (Figure 1), there was slight upward trending prior to tablet shipment (ie, tablet recipients appeared to be increasing video psychotherapy visits slightly over time compared with their baseline and compared with tablet nonrecipients prior to tablet shipment). However, posttablet shipment associations were abruptly different than the pretablet trend such that associations did not seem mere continuation of the pretablet trend. For all outcomes, results were similar for the same analyses conducted using the matched subcohort of veterans.

For the full cohort, tablets were associated with additional yearly increases for psychotherapy visits across all modalities (1.8 visits per year [monthly coefficient, 0.15; 95% CI, 0.13-0.17]), video psychotherapy visits (3.5 visits per year [monthly coefficient, 0.29; 95% CI, 0.27-0.31]), video medication management visits (0.7 visits per year [monthly coefficient, 0.06; 95% CI, 0.055-0.062]), and video visits for CSREs (0.02 visits per year [monthly coefficient, 0.002; 95% CI, 0.002-0.002]) (Table 2). Given baseline frequency of psychotherapy visits for tablet recipients in our full cohort was 5.5 visits per year (0.46 per month) and in the high-risk sub-cohort was 13.7 visits per year (1.14 per month) (Table 2), tablet-associated increases of 1.8 visits per year 8 and 3.1 visits per year for the full cohort and high-risk subcohort translated to increases of 33% and 23%, respectively. Tablets were associated with a decrease in the likelihood of an ED visit of 20% (proportion change, −0.012; 95% CI, −0.014 to −0.010), decrease in the likelihood of suicide-related ED visit of 36% (proportion change, −0.0017; 95% CI, −0.0023 to −0.0013), and 22% decrease in SBORs (monthly coefficient, −0.0011; 95% CI, −0.0016 to −0.0005). For the full cohort of tablet recipients, the results translate to a tablet-associated decrease of 158 ED visits and 24 suicide-related ED visits during the 10-month period and with about 168 fewer suicide behavior reports per year (Table 2).

**Table 2.** Adjusted Differences in Outcomes for Tablet Recipients vs Recipients’ Baseline and Nonrecipients^a^

Characteristic	Difference-in-difference coefficients (95% CI)						
	Psychotherapy (all modalities)	Video psychotherapy	Video medication management	Video visits for CSREs	Any ED visit (Y/N)	Any suicide-related ED Visit (Y/N)	VA SBORs
Full cohort of rural veterans with ≥1 VA mental health visit in 2019^b^							
TabletRecipient^c^	0.213 (0.20 to 0.23)	0.013 (0.008 to 0.02)	−0.001 (−0.002 to 0.0004)	0.0001 (−0.000 05 to 0.0002)	−0.0013 (−0.0031 to 0.0004)	0.0005 (0.000 02 to 0.0009)	0.0017 (0.0012 to 0.0021)
TabletRecipient × Posttablet^d^	0.151 (0.13 to 0.17)	0.291 (0.27 to 0.31)	0.058 (0.055 to 0.062)	0.002 (0.002 to 0.002)	−0.012 (−0.014 to −0.010)	−0.0018 (−0.0023 to −0.0013)	−0.0011 (−0.0016 to −0.0005)
% Change from recipients’ baseline^e^	32.8	243	193	200	−20.3	−36.0	−22.0
Tablets-associated change in the population, visits/mo^f^	+1990	+3835	+764	+26	−158^g^	−24^g^	−14^h^
Subcohort of rural veterans the VA identified as high-risk for suicide^b^							
TabletRecipient^c^	0.333 (0.24 to 0.43)	0.030 (0.004 to 0.06)	−0.002 (−0.007 to 0.003)	0.0001 (−0.001 to 0.001)	0.0086 (−0.0013 to 0.184)	0.0051 (0.0004 to 0.0097)	0.0102 (0.0060 to 0.0144)
TabletRecipient × Posttablet^d^	0.263 (0.15 to 0.38)	0.485 (0.39 to 0.58)	0.071 (0.06 to 0.09)	0.007 (0.004 to 0.01)	−0.033 (−0.044 to −0.023)	−0.012 (−0.017 to −0.0065)	−0.0075 (−0.125 to −0.0026)
% Change from recipients’ baseline^e^	23.0	169.0	161.4	700.0	−18.9	−25.5	−22.1
Tablets-associated change in the subpopulation, visits/mo^f^	+304	+561	+82	+8	−38^g^	−13^g^	−8^h^

Abbreviations: CSRE, comprehensive suicide risk evaluation; ED, emergency department; SBORs, suicide behavior and overdose reports; VA, US Department of Veterans Affairs.

^a^ We excluded month −1 and month 0 because treatment assignment (ie, tablet assignment) likely occurred in these months and we did not want to attribute tablet assignment-related visits to the tablet-associated outcomes. All models adjusted for veterans’ age, sex, race, number of physical and mental health chronic conditions, diagnoses of substance use disorder, posttraumatic stress disorder and depression, VA-estimated 1-year probability of hospitalization or death, VA priority-based enrollment, marital status, homelessness indicator, high suicide risk indicator, cumulative monthly COVID-19 cases in the patient’s county. All models included indicators for calendar month to adjust for events occurring in each month and indicators for patients’ closest secondary care facility to control for any time-invariant facility characteristics. In all models, standard errors accounted for clustering at the patient-level.

^b^ The full-cohort analyses included 471 791 rural veterans (13 180 of whom were tablet recipients) and 17 794 410 veteran-monthly observations. The high-risk sub-cohort analyses included 16 662 rural veterans (1157 of whom were tablet recipients) and 591 066 veteran-monthly observations.

^c^ The coefficient on the variable TabletRecipient indicates the fixed difference between tablet recipients and nonrecipients.

^d^ The coefficient on TabletRecipient × Posttablet represents difference-in-differences estimate (ie, it averages the associations of tablets across all posttablet months). These coefficients represent monthly changes in visits or in likelihood of visits. The monthly change in likelihood of visits is the same as an estimated yearly change in likelihood of visits. For outcomes looking at the number of visits, we multiply coefficients by 12 to estimate yearly changes in the number of visits reported in the study.

^e^ Full-cohort tablet recipients’ baseline means used for calculating percentage change were: psychotherapy (all modalities), 0.46 per month; video psychotherapy, 0.12 per month; video medication management, 0.03 per month; video CSREs, 0.001 per month; any ED visit, 0.059 per month; any suicide-related ED visit, 0.005 per month; VA SBORs, 0.005 per month. Subcohort tablet recipients’ baseline means used for calculating percentage change were: psychotherapy (all modalities), 1.142 per month; video psychotherapy, 0.287 per month, video medication management, 0.044 per month; video CSREs, 0.001 per month; any ED visit, 0.175 per month; any suicide-related ED visit, 0.047 per month; VA SBORs, 0.034 per month.

^f^ To calculate the population-level and subpopulation-level estimates, we multiplied the difference-in-difference estimates in the previous rows by the size of the cohort, 13 180, and subcohort, 1157, respectively.

^g^ Measured as change in the population visits per 10-month period.

^h^ Measured as SBORs per month.

For the subcohort of rural veterans at high-risk for suicide, tablets were associated with an increase in the yearly psychotherapy visits across all modalities (3.1 visits per year [monthly coefficient, 0.26; 95% CI, 0.15-0.38]), an increase in video psychotherapy visits (5.9 visits per year [monthly coefficient, 0.49; 95% CI, 0.39-0.58), an increase in video medication management visits (0.8 visits per year [monthly coefficient, 0.07; 95% CI, 0.06-0.09]), and an increase in video visits for CSREs (0.1 visits per year [monthly coefficient, 0.007; 95% CI, 0.004-0.01]). For this subcohort, tablets were associated with a decrease in the likelihood of an ED visit of 19% (proportion change, −0.033; 95% CI, −0.044 to −0.023), a decrease in the likelihood of a suicide-related visit of 26% (proportion change, −0.012; 95% CI, −0.017 to −0.0065), and a decrease in SBORs of 22% (monthly coefficient, −0.0075; 95% CI, −0.125 to −0.0026). For the subcohort of tablet recipients considered high-risk for suicide, the results translate to a tablet-associated decrease of 38 ED visits and 13 suicide-related ED visits during the 10-month period and with about 96 fewer suicide behavior reports per year (Table 2).

## Discussion

To our knowledge, this study was the largest evaluation of a health system intervention to distribute video-enabled telehealth tablets to patients with access barriers and mental health needs. We examined associations between VA’s video-enabled tablets distributed during COVID-19 and veterans’ mental health care use, suicide behavior, and ED visits. We leveraged differential timing of tablet issuance across veterans and compared outcomes for rural tablet recipients and nonrecipients, 10 months before and after tablet-shipment, to isolate associations occurring posttablet issuance. Tablets were associated with additional 3.5 video psychotherapy visits per year for the full rural cohort and with 5.9 video visits per year for the subcohort at high-risk for suicide. The tablet-associated increase in psychotherapy visits across all modalities was smaller than the tablet-associated increase in video psychotherapy visits, at 1.8 and 3.1 psychotherapy visits per year for the full cohort and high-risk cohort, respectively, because video visits replaced phone or in-person visits in some cases whereas in other cases video visits were new or additional visits. These results reinforced a previous finding that tablets improved continuity of mental health care, and extended prior work by showing tablet-associated reductions in ED visits and suicide behavior.^16^

To contextualize these tablet associations, note that baseline frequency of psychotherapy visits for tablet recipients in our full cohort was 5.5 visits per year (0.46 per month) and in the high-risk subcohort was 13.7 visits per year (1.14 per month) (Table 2). Thus, tablet-associated increases of 1.8 visits per year and 3.1 visits per year for the full cohort and high-risk sub-cohort translated to increases of 33% and 23%, respectively. Clinical importance of these tablet-associations is supported by studies showing that each psychotherapy session leads to patient improvements (if total visits do not exceed 26)^47,48,49^; studies have shown that 1 to 2 psychotherapy sessions were associated with an additional 10% to 16% of patients improving,^50,51,52^ and that the session range required for patient improvement for low-risk patients was 2 to 13 and for high-risk patients was 2 to 25.^52^ Studies have shown that less or equal to 2 sessions per year reduced suicide attempts and adverse symptoms.^53^

Clinical importance is further emphasized by our complementary findings that tablets were associated with decreases in likelihood of ED visits and suicide-related ED visits, and decreases in suicide behavior and overdose reports. As these were infrequent outcomes (Table 1), program-level estimates are helpful. The VA’s tablet distribution during COVID-19 was associated with approximate decreases of 158 ED visits and 24 suicide-related ED visits during the 10-month period and with about 168 fewer suicide behavior reports per year (Table 2). Compared with the baseline number of tablet recipients with at least 1 ED visit (n = 775), and at least 1 suicide-related ED visit (n = 68) (Table 1), the tablet-associations translated to reductions of 36% and 22%, respectively. These tablet-associations were not readily comparable with prior studies that focused on specific suicide-related interventions whereas tablets likely allowed simultaneous access to many types of care or applications. Nevertheless, the magnitudes of these associations were consistent with prior work on suicide-related interventions showing reductions in suicide behavior of 30%^54^ and 45%.^27^

### Limitations

This study had some limitations. A natural limitation of evaluating a health system initiative like tablets was that tablet assignments were nonrandom. There were baseline differences between rural tablet recipients and rural nonrecipients; recipients were more likely to engage in mental health service use, ED use, and had more clinical indicators for poor health. To address these concerns, we leveraged the difference-in-differences approach which allowed for such level differences between tablet recipients and nonrecipients, but was sensitive to differences in unobserved trends that influence outcomes and tablet receipt. Difference-in-differences can handle the existence of unobserved drivers of outcomes so long as unobserved factors do not lead to differential trends for tablet recipients and nonrecipients in the posttablet period. Using the VA’s rich data, our models adjusted for several patient characteristics and leveraged the large cohort size and many periods to explicitly adjust for any remaining fixed difference between tablet recipients and nonrecipients (details in eAppendix 1 in the Supplement). As the assumption of similar trends for recipients and nonrecipients is key for assessing validity of difference-in-differences, we used event studies that improve upon the usual difference-in-differences by allowing direct empirical testing of adjusted pretablet trends. We also provided unadjusted monthly outcome graphs going back to 2018 (eFigure 3 in the Supplement) to show that tablet recipients and nonrecipients had similar trends. It is rare for difference-in-differences analyses to have so many periods of pre-treatment data for assessing the trends assumption. Thus, although there were unavoidable methodological constraints in using real-world data for evaluating tablets, our rigorous methods enhanced attributability of results to tablets.

Another limitation was the difficulty in disentangling the COVID-19 pandemic-related associations from tablet-related associations. To address this, we included in our models the monthly number of COVID-19 cases in each county and included month indicators to capture any remaining secular shocks occurring in each month (eAppendix 1 in the Supplement). Nonetheless, if unobserved factors during the pandemic led to differential post-pandemic trends such as tablet recipients (nonrecipients) reducing ED visits more than nonrecipients (tablet recipients), then ED reductions observed would be overstating (understating) true tablet associations. To address this, we provided calendar-time graphs showing that the pandemic did not differentially affect tablet recipients and nonrecipients such that nonrecipients provided a strong counterfactual trend for recipients even during the COVID-19 period (eFigure 3 in the Supplement). Calendar-time graphs (eFigure 3 in the Supplement) showed that post-pandemic declines in care utilization occurred similarly for both tablet recipients and nonrecipients, and only when adjusted outcomes were viewed in time-relative-to-tablet-shipment (Figure 1 and Figure 2), tablet-specific associations were isolated. The methods used enhanced confidence that observed associations were attributable to tablets.

The scope of this study was limited. We could not analyze all potential mechanisms through which tablets may reduce suicide behavior and ED visits (eg, other mental health services, physical health care services, care timeliness or convenience, and social and digital connectivity). Future studies should examine the range of mechanisms and outcomes tablets can influence, as well as tablet-associated program and utilization costs. As these results may not readily generalize to non-VA settings, studies examining device-enabled virtual care outside the VA are also needed.

## Conclusions

This cohort study of the VA’s distribution of video-enabled tablets to rural veterans during the COVID-19 pandemic suggests that tablet-receipt was associated with increased video mental health service use, increased psychotherapy visits, reduced suicide behavior, and reduced ED visits. These findings suggest that the VA and other health systems should consider leveraging video-enabled tablets for improving access to mental health care via telehealth and for preventing suicides among rural residents.

## Notes

Supplement. **eFigure 1.** Flow Chart Describing the Construction of the Study Cohort and Sub-Cohort **eTable 1.** Criteria for Identifying Psychotherapy Visits **eTable 2.** CPT Codes Used as Part of the Criteria for Identifying Psychotherapy Visits **eTable 3.** Criteria for Identifying Medication Management Visits **eTable 4.** Mental Health VA E&M Stop Codes, Used as Part of the Criteria for Identifying Medication Management Visits **eTable 5.** E&M CPT Codes, Used as Part of the Criteria for Identifying Medication Management Visits **eTable 6.** VA Codes for Providers and Qualified Prescribers, Used as Part of the Criteria for Identifying Medication Management Visits **eTable 7.** ICD-10 Codes for Identifying Mental Health Conditions, Used as Part of the Criteria for Identifying Psychotherapy and Medication Management Visits **eTable 8.** ICD-10 Codes Used for Identifying Suicide-Related ED Visits **eTable 9.** Physical and Mental Health Chronic Condition Included as Covariates **eAppendix 1.** Methods Details **eFigure 2.** Tablet Recipients’ Visits for Medication Management and CSREs Compared to the Baseline and Compared to Non-Recipients - Regression Coefficients From Event Study Specifications **eFigure 3.** Unadjusted Average Number of Monthly Visit Outcomes for Rural Tablet Recipients and Rural Tablet Non-Recipients by Calendar Month-Year
