# Supplementary material for: Mental Health Service Use, Suicide Behavior, and Emergency Department Visits Among Rural US Veterans Who Received Video-Enabled Tablets During the COVID-19 Pandemic
Source: JAMA Netw Open. 2022 Apr 6;5(4):e226250. doi: 10.1001/jamanetworkopen.2022.6250 (PMC8987904; doi:10.1001/jamanetworkopen.2022.6250)
Supplement: Supplement. — eFigure 1. Flow Chart Describing the Construction of the Study Cohort and Sub-Cohort eTable 1. Criteria for Identifying Psychotherapy Visits eTable 2. CPT Codes Used as Part of the Criteria for Identifying Psychotherapy Visits eTable 3. Criteria for Identifying Medication Management Visits eTable 4. Mental Health VA E&M Stop Codes, Used as Part of the Criteria for Identifying Medication Management Visits eTable 5. E&M CPT Codes, Used as Part of the Criteria for Identifying Medication Management Visits eTable 6. VA Codes for Providers and Qualified Prescribers, Used as Part of the Criteria for Identifying Medication Management Visits eTable 7. ICD-10 Codes for Identifying Mental Health Conditions, Used as Part of the Criteria for Identifying Psychotherapy and Medication Management Visits eTable 8. ICD-10 Codes Used for Identifying Suicide-Related ED Visits eTable 9. Physical and Mental Health Chronic Condition Included as Covariates eAppendix 1. Methods Details eFigure 2. Tablet Recipients’ Visits for Medication Management and CSREs Compared to the Baseline and Compared to Non-Recipients - Regression Coefficients From Event Study Specifications eFigure 3. Unadjusted Average Number of Monthly Visit Outcomes for Rural Tablet Recipients and Rural Tablet Non-Recipients by Calendar Month-Year [file jamanetwopen-e226250-s001.pdf]

Supplemental Online Content

Gujral K, Van Campen J, Jacobs J, Kimerling R, Blonigen D, Zulman DM. Mental health service use, suicide behavior, and emergency department visits among US rural veterans who received video-enabled tablets during the COVID-19 pandemic. *JAMA Netw Open.* 2022;5(4):e226250. doi:10.1001/jamanetworkopen.2022.6250

eFigure 1. Flow Chart Describing the Construction of Our Cohort and Sub-Cohort

eTable 1. Criteria for Identifying Psychotherapy Visits

eTable 2. CPT Codes Used as Part of the Criteria for Identifying Psychotherapy Visits

eTable 3. Criteria for Identifying Medication Management Visits

eTable 4. Mental Health VA E&M Stop Codes, Used as Part of the Criteria for Identifying Medication Management Visits

eTable 5. E&M CPT Codes, Used as Part of the Criteria for Identifying Medication Management Visits

eTable 6. VA Codes for Providers and Qualified Prescribers, Used as Part of the Criteria for Identifying Medication Management Visits

eTable 7. ICD-10 Codes for Identifying Mental Health Conditions, Used as Part of the Criteria for Identifying Psychotherapy and Medication Management Visits

eTable 8. ICD-10 Codes Used for Identifying Suicide-Related ED Visits

eTable 9. Physical and Mental Health Chronic Condition Included as Covariates

eAppendix 1. Methods Details

eFigure 2. Tablet Recipients' Visits for Medication Management and CSRES Compared to the Baseline and Compared to Non-Recipients - Regression Coefficients From Event Study Specifications

eFigure 3. Unadjusted Average Number of Monthly Visit Outcomes for Rural Tablet Recipients and Rural Tablet Non-Recipients by Calendar Month-Year

This supplemental material has been provided by the authors to give readers additional information about their work.

© 2022 Gujral K et al. *JAMA Network Open.*

eFigure 1: Flow chart describing the construction of our cohort and sub-cohort

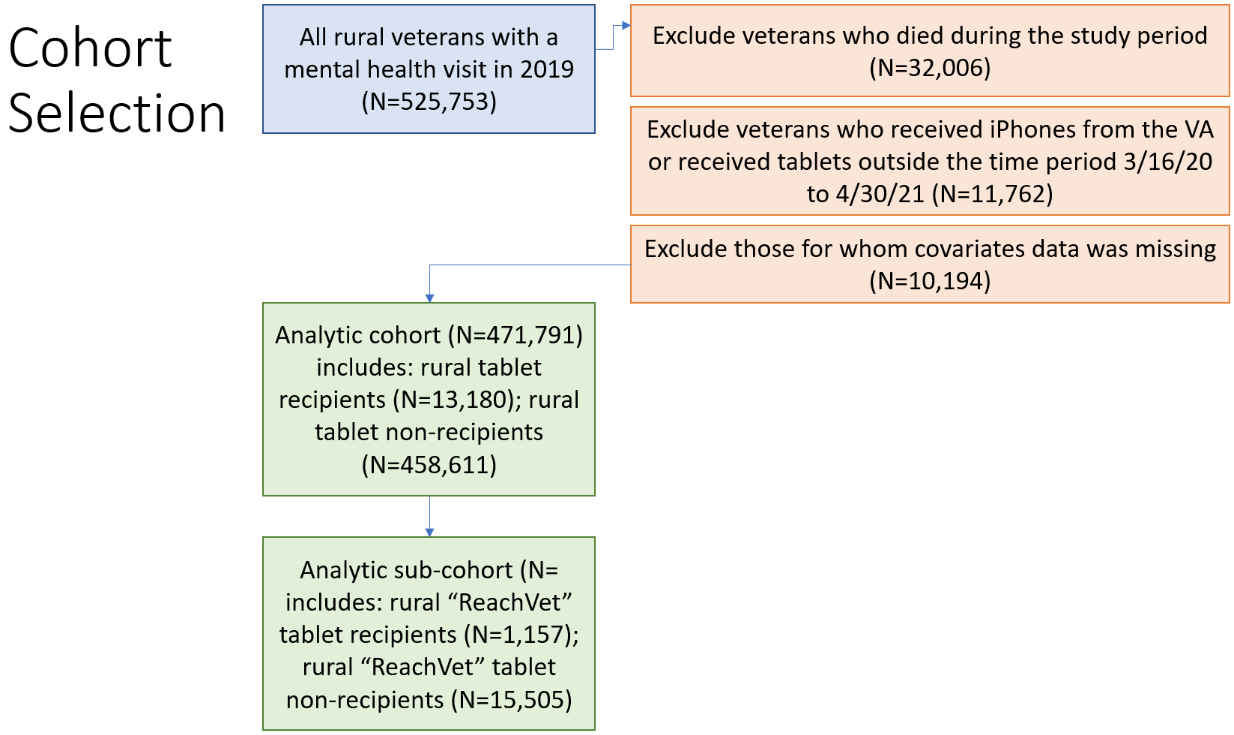

eTable 1: Criteria for identifying psychotherapy visits

**Psychotherapy Visit Criteria:**
Depression Diagnosis and depression current procedural terminology (CPT) code
or Serious Mental Illness (SMI) diagnosis and SMI CPT code
or SUD diagnosis and SUD CPT code
or Post-Traumatic Stress Disorder (PTSD) diagnosis and PTSD CPT code

eTable 2: CPT codes used as part of the criteria for identifying psychotherapy visits

**CPT Codes for Depression and PTSD**	
**CPT Code**	**CPT Description**
90791	PSYCHIATRIC DIAGNOSTIC EVALUATION
90832	PSYCHOTHERAPY, 30 MINUTES WITH PATIENT
90834	PSYCHOTHERAPY, 45 MINUTES WITH PATIENT
90837	PSYCHOTHERAPY, 60 MINUTES WITH PATIENT
90839	PSYCHOTHERAPY FOR CRISIS; FIRST 60 MINUTES
90846	FAMILY PSYCHOTHERAPY (WITHOUT THE PATIENT PRESENT), 50 MINUTES
90847	FAMILY PSYCHOTHERAPY (CONJOINT PSYCHOTHERAPY) (WITH PATIENT PRESENT), 50 MINUTES

© 2022 Gujral K et al. *JAMA Network Open.*

90849	MULTIPLE-FAMILY GROUP PSYCHOTHERAPY
90853	GROUP PSYCHOTHERAPY (OTHER THAN OF A MULTIPLE-FAMILY GROUP)
**CPT Codes for SMI**	
**CPT Code**	**CPT Description**
90791	PSYCHIATRIC DIAGNOSTIC EVALUATION
90832	PSYCHOTHERAPY, 30 MINUTES WITH PATIENT
90834	PSYCHOTHERAPY, 45 MINUTES WITH PATIENT
90837	PSYCHOTHERAPY, 60 MINUTES WITH PATIENT
90839	PSYCHOTHERAPY FOR CRISIS; FIRST 60 MINUTES
90846	FAMILY PSYCHOTHERAPY (WITHOUT THE PATIENT PRESENT), 50 MINUTES
90847	FAMILY PSYCHOTHERAPY (CONJOINT PSYCHOTHERAPY) (WITH PATIENT PRESENT), 50 MINUTES
90849	MULTIPLE-FAMILY GROUP PSYCHOTHERAPY
90853	GROUP PSYCHOTHERAPY (OTHER THAN OF A MULTIPLE-FAMILY GROUP)
98961	EDUCATION AND TRAINING FOR PATIENT SELF-MANAGEMENT BY A QUALIFIED, NONPHYSICIAN HEALTH CARE PROFESSIONAL USING A STANDARDIZED CURRICULUM, FACE-TO-FACE WITH THE PATIENT (COULD INCLUDE CAREGIVER/FAMILY) EACH 30 MINUTES; 2-4 PATIENTS
98962	EDUCATION AND TRAINING FOR PATIENT SELF-MANAGEMENT BY A QUALIFIED, NONPHYSICIAN HEALTH CARE PROFESSIONAL USING A STANDARDIZED CURRICULUM, FACE-TO-FACE WITH THE PATIENT (COULD INCLUDE CAREGIVER/FAMILY) EACH 30 MINUTES; 5-8 PATIENTS
99078	PHYSICIAN OR OTHER QUALIFIED HEALTH CARE PROFESSIONAL QUALIFIED BY EDUCATION, TRAINING, LICENSURE/REGULATION (WHEN APPLICABLE) EDUCATIONAL SERVICES RENDERED TO PATIENTS IN A GROUP SETTING (EG, PRENATAL, OBESITY, OR DIABETIC INSTRUCTIONS)
99509	HOME VISIT FOR ASSISTANCE WITH ACTIVITIES OF DAILY LIVING AND PERSONAL CARE
99510	HOME VISIT FOR INDIVIDUAL, FAMILY, OR MARRIAGE COUNSELING
**CPT Codes for SUD (PSY36)**	
**CP TCode**	**CPT Description**
90791	PSYCHIATRIC DIAGNOSTIC EVALUATION
90832	PSYCHOTHERAPY, 30 MINUTES WITH PATIENT
90834	PSYCHOTHERAPY, 45 MINUTES WITH PATIENT
90837	PSYCHOTHERAPY, 60 MINUTES WITH PATIENT
90839	PSYCHOTHERAPY FOR CRISIS; FIRST 60 MINUTES
90846	FAMILY PSYCHOTHERAPY (WITHOUT THE PATIENT PRESENT), 50 MINUTES
90847	FAMILY PSYCHOTHERAPY (CONJOINT PSYCHOTHERAPY) (WITH PATIENT PRESENT), 50 MINUTES
90849	MULTIPLE-FAMILY GROUP PSYCHOTHERAPY
90853	GROUP PSYCHOTHERAPY (OTHER THAN OF A MULTIPLE-FAMILY GROUP)
99078	PHYSICIAN OR OTHER QUALIFIED HEALTH CARE PROFESSIONAL QUALIFIED BY EDUCATION, TRAINING, LICENSURE/REGULATION (WHEN APPLICABLE) EDUCATIONAL SERVICES RENDERED TO PATIENTS IN A GROUP SETTING (EG, PRENATAL, OBESITY, OR DIABETIC INSTRUCTIONS)

© 2022 Gujral K et al. *JAMA Network Open.*

eTable 3: Criteria for identifying medication management visits

**Medication Management Visit Criteria:**
Provider is either:
a psychiatrist
or another qualified prescriber and the visit has a mental health evaluation and management (E&M) stop code in the primary or secondary position.
Visit has E&M CPT code
Mental health or Substance Use Disorder (SUD) diagnosis.

eTable 4: Mental health VA E&M stop codes, used as part of the criteria for identifying medication management visits

**Mental Health E&M Stop Codes**	
**VA 3-digit Stop Code**	**Stop Code Name**
156	HBPC - PSYCHOLOGIST
157	HBPC - PSYCHIATRIST
292	OBSERVATION PSYCHIATRY
502	MENTAL HEALTH CLINIC - INDIVIDUAL
503	MENTAL HEALTH RESIDENTIAL CARE - INDIVIDUAL
505	DAY TREATMENT - INDIVIDUAL
506	DAY HOSPITAL - INDIVIDUAL
509	PSYCHIATRY
510	PSYCHOLOGY
512	MENTAL HEALTH CONSULTATION
513	SUBSTANCE USE DISORDER - INDIVIDUAL
514	SUBSTANCE USE DISORDER - HOME VISIT
516	PTSD - GROUP
519	SUBSTANCE USE DISORDER/PTSD TEAMS
523	OPIOID TREATMENT PROGRAM
524	ACTIVE DUTY SEXUAL TRAUMA
525	WOMEN'S STRESS DISORDER TREATMENT TEAMS
527	TELEPHONE MENTAL HEALTH
531	MENTAL HEALTH PRIMARY CARE - INDIVIDUAL
533	MENTAL HEALTH INTERVENTION BIOMEDICAL CARE - INDIVIDUAL
534	MENTAL HEALTH INTEGRATED CARE - INDIVIDUAL
535	MENTAL HEALTH VOCATIONAL ASSISTANCE - INDIVIDUAL
536	TELEPHONE MENTAL HEALTH VOCATIONAL ASSISTANCE
538	PSYCHOLOGICAL TESTING
539	MENTAL HEALTH INTEGRATED CARE - GROUP
540	PTSD CLINICAL TEAM (PCT) POST-TRAUMATIC STRESS - INDVIDUAL
542	TELEPHONE/POST-TRAUMATIC STRESS DISORDER (PTSD)
545	TELEPHONE/SUBSTANCE USE DISORDER

© 2022 Gujral K et al. *JAMA Network Open.*

546	TELEPHONE INTENSIVE COMMUNITY MENTAL HEALTH RECOVERY SERVICES (ICMHR)
547	INTENSIVE SUBSTANCE USE DISORDER - GROUP
548	INTENSIVE SUBSTANCE USE DISORDER - INDIVIDUAL
550	MENTAL HEALTH CLINIC - GROUP
552	INTENSIVE COMMUNITY MENTAL HEALTH RECOVERY SERVICES (ICMHR)-INDIVIDUAL
553	DAY TREATMENT - GROUP
554	DAY HOSPITAL - GROUP
555	HOMELESS VETERAN COMMUNITY EMPLOYMENT SERVICES - INDIVIDUAL
556	HOMELESS VETERAN COMMUNITY EMPLOYMENT SERVICES - GROUP
557	PSYCHIATRY - GROUP
558	PSYCHOLOGY - GROUP
560	SUBSTANCE USE DISORDER - GROUP
561	PCT-POST TRAUMATIC STRESS - GROUP
562	PTSD - INDIVIDUAL
564	MENTAL HEALTH TEAM CASE MANAGEMENT
565	MENTAL HEALTH INTERVENTION BIOMEDICAL CARE - GROUP
566	MENTAL HEALTH RISK-FACTOR REDUCTION EDUCATIONAL GROUP
567	INTENSIVE COMMUNITY MENTAL HEALTH RECOVERY SERVICES (ICMHR)- GROUP
568	MENTAL HEALTH COMPENSATED WORK THERAPY/ SUPPORTED EMPLOYMENT (CWT/SE) FACE-TO-FACE
571	SERV-MH (SERVICES FOR RETURNING VETERANS-MENTAL HEALTH) - INDIVIDUAL
572	SERV-MH (SERVICES FOR RETURNING VETERANS-MENTAL HEALTH) - GROUP
573	MENTAL HEALTH INCENTIVE THERAPY FACE-TO-FACE
574	MENTAL HEALTH COMPENSATED WORK THERAPY/TRANSITIONAL WORK EXPERIENCE (CWT/TWE) FACE-TO-FACE
575	MENTAL HEALTH VOCATIONAL ASSISTANCE - GROUP
576	PSYCHOGERIATRIC CLINIC - INDIVIDUAL
577	PSYCHOGERIATRIC CLINIC - GROUP
579	TELEPHONE/PSYCHOGERIATRICS
580	PTSD DAY HOSPITAL
582	PSYCHOSOCIAL REHABILITATION RECOVERY CENTER (PRRC) - INDIVIDUAL
583	PSYCHOSOCIAL REHABILITATION RECOVERY CENTER (PRRC)- GROUP
584	TELEPHONE PSYCHOSOCIAL REHABILITATION RECOVERY CENTER (PRRC)
586	RESIDENTIAL REHABILITATION TREATMENT PROGRAM (RRTP) - INDIVIDUAL
587	RESIDENTIAL REHABILITATION TREATMENT PROGRAM (RRTP) - GROUP
588	RESIDENTIAL REHABILITATION TREATMENT PROGRAM (RRTP) AFTERCARE - INDIVIDUAL
593	RESIDENTIAL REHABILITATION TREATMENT PROGRAM (RRTP) OUTREACH SERVICES
595	RESIDENTIAL REHABILITATION TREATMENT PROGRAM (RRTP) AFTERCARE - GROUP
596	RESIDENTIAL REHABILITATION TREATMENT PROGRAM (RRTP) ADMISSION SCREENING SERVICES

© 2022 Gujral K et al. *JAMA Network Open.*

597	TELEPHONE/RESIDENTIAL REHABILITATION TREATMENT PROGRAM (RRTP)
598	RESIDENTIAL REHABILITATION TREATMENT PROGRAM (RRTP) OUTPATIENT – INDIVIDUAL
599	RESIDENTIAL REHABILITATION TREATMENT PROGRAM (RRTP) OUTPATIENT – GROUP
713	GAMBLING ADDICTION

eTable 5: E&M CPT codes, used as part of the criteria for identifying medication management visits

**E&M CPT Codes**	
**CPT Code**	**CPT Description**
90792	PSYCHIATRIC DIAGNOSTIC EVALUATION WITH MEDICAL SERVICES
90863	PHARMACOLOGIC MANAGEMENT, INCLUDING PRESCRIPTION AND REVIEW OF MEDICATION, WHEN PERFORMED WITH PSYCHOTHERAPY SERVICES (LIST SEPARATELY IN ADDITION TO THE CODE FOR PRIMARY PROCEDURE)
99202	OFFICE OR OTHER OUTPATIENT VISIT FOR THE EVALUATION AND MANAGEMENT OF A NEW PATIENT, WHICH REQUIRES A MEDICALLY APPROPRIATE HISTORY AND/OR EXAMINATION AND STRAIGHTFORWARD MEDICAL DECISION MAKING. WHEN USING TIME FOR CODE SELECTION, 15-29 MINUTES OF TOTAL TIME IS SPENT ON THE DATE OF THE ENCOUNTER.
99203	OFFICE OR OTHER OUTPATIENT VISIT FOR THE EVALUATION AND MANAGEMENT OF A NEW PATIENT, WHICH REQUIRES A MEDICALLY APPROPRIATE HISTORY AND/OR EXAMINATION AND LOW LEVEL OF MEDICAL DECISION MAKING. WHEN USING TIME FOR CODE SELECTION, 30-44 MINUTES OF TOTAL TIME IS SPENT ON THE DATE OF THE ENCOUNTER.
99204	OFFICE OR OTHER OUTPATIENT VISIT FOR THE EVALUATION AND MANAGEMENT OF A NEW PATIENT, WHICH REQUIRES A MEDICALLY APPROPRIATE HISTORY AND/OR EXAMINATION AND MODERATE LEVEL OF MEDICAL DECISION MAKING. WHEN USING TIME FOR CODE SELECTION, 45-59 MINUTES OF TOTAL TIME IS SPENT ON THE DATE OF THE ENCOUNTER.
99205	OFFICE OR OTHER OUTPATIENT VISIT FOR THE EVALUATION AND MANAGEMENT OF A NEW PATIENT, WHICH REQUIRES A MEDICALLY APPROPRIATE HISTORY AND/OR EXAMINATION AND HIGH LEVEL OF MEDICAL DECISION MAKING. WHEN USING TIME FOR CODE SELECTION, 60-74 MINUTES OF TOTAL TIME IS SPENT ON THE DATE OF THE ENCOUNTER.
99213	OFFICE OR OTHER OUTPATIENT VISIT FOR THE EVALUATION AND MANAGEMENT OF AN ESTABLISHED PATIENT, WHICH REQUIRES A MEDICALLY APPROPRIATE HISTORY AND/OR EXAMINATION AND LOW LEVEL OF MEDICAL DECISION MAKING. WHEN USING TIME FOR CODE SELECTION, 20-29 MINUTES OF TOTAL TIME IS SPENT ON THE DATE OF THE ENCOUNTER.
99214	OFFICE OR OTHER OUTPATIENT VISIT FOR THE EVALUATION AND MANAGEMENT OF AN ESTABLISHED PATIENT, WHICH REQUIRES A MEDICALLY APPROPRIATE HISTORY AND/OR EXAMINATION AND MODERATE LEVEL OF MEDICAL DECISION MAKING. WHEN USING TIME FOR CODE SELECTION, 30-39 MINUTES OF TOTAL TIME IS SPENT ON THE DATE OF THE ENCOUNTER.

© 2022 Gujral K et al. *JAMA Network Open.*

99215	OFFICE OR OTHER OUTPATIENT VISIT FOR THE EVALUATION AND MANAGEMENT OF AN ESTABLISHED PATIENT, WHICH REQUIRES A MEDICALLY APPROPRIATE HISTORY AND/OR EXAMINATION AND HIGH LEVEL OF MEDICAL DECISION MAKING. WHEN USING TIME FOR CODE SELECTION, 40-54 MINUTES OF TOTAL TIME IS SPENT ON THE DATE OF THE ENCOUNTER.
99342	HOME VISIT FOR THE EVALUATION AND MANAGEMENT OF A NEW PATIENT, WHICH REQUIRES THESE 3 KEY COMPONENTS: AN EXPANDED PROBLEM FOCUSED HISTORY; AN EXPANDED PROBLEM FOCUSED EXAMINATION; AND MEDICAL DECISION MAKING OF LOW COMPLEXITY. COUNSELING AND/OR COORDINATION OF CARE WITH OTHER PHYSICIANS, OTHER QUALIFIED HEALTH CARE PROFESSIONALS, OR AGENCIES ARE PROVIDED CONSISTENT WITH THE NATURE OF THE PROBLEM(S) AND THE PATIENT'S AND/OR FAMILY'S NEEDS. USUALLY, THE PRESENTING PROBLEM(S) ARE OF MODERATE SEVERITY. TYPICALLY, 30 MINUTES ARE SPENT FACE-TO-FACE WITH THE PATIENT AND/OR FAMILY.
99343	HOME VISIT FOR THE EVALUATION AND MANAGEMENT OF A NEW PATIENT, WHICH REQUIRES THESE 3 KEY COMPONENTS: A DETAILED HISTORY; A DETAILED EXAMINATION; AND MEDICAL DECISION MAKING OF MODERATE COMPLEXITY. COUNSELING AND/OR COORDINATION OF CARE WITH OTHER PHYSICIANS, OTHER QUALIFIED HEALTH CARE PROFESSIONALS, OR AGENCIES ARE PROVIDED CONSISTENT WITH THE NATURE OF THE PROBLEM(S) AND THE PATIENT'S AND/OR FAMILY'S NEEDS. USUALLY, THE PRESENTING PROBLEM(S) ARE OF MODERATE TO HIGH SEVERITY. TYPICALLY, 45 MINUTES ARE SPENT FACE-TO-FACE WITH THE PATIENT AND/OR FAMILY.
99344	HOME VISIT FOR THE EVALUATION AND MANAGEMENT OF A NEW PATIENT, WHICH REQUIRES THESE 3 KEY COMPONENTS: A COMPREHENSIVE HISTORY; A COMPREHENSIVE EXAMINATION; AND MEDICAL DECISION MAKING OF MODERATE COMPLEXITY. COUNSELING AND/OR COORDINATION OF CARE WITH OTHER PHYSICIANS, OTHER QUALIFIED HEALTH CARE PROFESSIONALS, OR AGENCIES ARE PROVIDED CONSISTENT WITH THE NATURE OF THE PROBLEM(S) AND THE PATIENT'S AND/OR FAMILY'S NEEDS. USUALLY, THE PRESENTING PROBLEM(S) ARE OF HIGH SEVERITY. TYPICALLY, 60 MINUTES ARE SPENT FACE-TO-FACE WITH THE PATIENT AND/OR FAMILY.
99345	HOME VISIT FOR THE EVALUATION AND MANAGEMENT OF A NEW PATIENT, WHICH REQUIRES THESE 3 KEY COMPONENTS: A COMPREHENSIVE HISTORY; A COMPREHENSIVE EXAMINATION; AND MEDICAL DECISION MAKING OF HIGH COMPLEXITY. COUNSELING AND/OR COORDINATION OF CARE WITH OTHER PHYSICIANS, OTHER QUALIFIED HEALTH CARE PROFESSIONALS, OR AGENCIES ARE PROVIDED CONSISTENT WITH THE NATURE OF THE PROBLEM(S) AND THE PATIENT'S AND/OR FAMILY'S NEEDS. USUALLY, THE PATIENT IS UNSTABLE OR HAS DEVELOPED A SIGNIFICANT NEW PROBLEM REQUIRING IMMEDIATE PHYSICIAN ATTENTION. TYPICALLY, 75 MINUTES ARE SPENT FACE-TO-FACE WITH THE PATIENT AND/OR FAMILY.

© 2022 Gujral K et al. *JAMA Network Open.*

99348	HOME VISIT FOR THE EVALUATION AND MANAGEMENT OF AN ESTABLISHED PATIENT, WHICH REQUIRES AT LEAST 2 OF THESE 3 KEY COMPONENTS: AN EXPANDED PROBLEM FOCUSED INTERVAL HISTORY; AN EXPANDED PROBLEM FOCUSED EXAMINATION; MEDICAL DECISION MAKING OF LOW COMPLEXITY. COUNSELING AND/OR COORDINATION OF CARE WITH OTHER PHYSICIANS, OTHER QUALIFIED HEALTH CARE PROFESSIONALS, OR AGENCIES ARE PROVIDED CONSISTENT WITH THE NATURE OF THE PROBLEM(S) AND THE PATIENT'S AND/OR FAMILY'S NEEDS. USUALLY, THE PRESENTING PROBLEM(S) ARE OF LOW TO MODERATE SEVERITY. TYPICALLY, 25 MINUTES ARE SPENT FACE-TO-FACE WITH THE PATIENT AND/OR FAMILY.
99349	HOME VISIT FOR THE EVALUATION AND MANAGEMENT OF AN ESTABLISHED PATIENT, WHICH REQUIRES AT LEAST 2 OF THESE 3 KEY COMPONENTS: A DETAILED INTERVAL HISTORY; A DETAILED EXAMINATION; MEDICAL DECISION MAKING OF MODERATE COMPLEXITY. COUNSELING AND/OR COORDINATION OF CARE WITH OTHER PHYSICIANS, OTHER QUALIFIED HEALTH CARE PROFESSIONALS, OR AGENCIES ARE PROVIDED CONSISTENT WITH THE NATURE OF THE PROBLEM(S) AND THE PATIENT'S AND/OR FAMILY'S NEEDS. USUALLY, THE PRESENTING PROBLEM(S) ARE MODERATE TO HIGH SEVERITY. TYPICALLY, 40 MINUTES ARE SPENT FACE-TO-FACE WITH THE PATIENT AND/OR FAMILY.
99350	HOME VISIT FOR THE EVALUATION AND MANAGEMENT OF AN ESTABLISHED PATIENT, WHICH REQUIRES AT LEAST 2 OF THESE 3 KEY COMPONENTS: A COMPREHENSIVE INTERVAL HISTORY; A COMPREHENSIVE EXAMINATION; MEDICAL DECISION MAKING OF MODERATE TO HIGH COMPLEXITY. COUNSELING AND/OR COORDINATION OF CARE WITH OTHER PHYSICIANS, OTHER QUALIFIED HEALTH CARE PROFESSIONALS, OR AGENCIES ARE PROVIDED CONSISTENT WITH THE NATURE OF THE PROBLEM(S) AND THE PATIENT'S AND/OR FAMILY'S NEEDS. USUALLY, THE PRESENTING PROBLEM(S) ARE OF MODERATE TO HIGH SEVERITY. THE PATIENT MAY BE UNSTABLE OR MAY HAVE DEVELOPED A SIGNIFICANT NEW PROBLEM REQUIRING IMMEDIATE PHYSICIAN ATTENTION. TYPICALLY, 60 MINUTES ARE SPENT FACE-TO-FACE WITH THE PATIENT AND/OR FAMILY.
99442	TELEPHONE EVALUATION AND MANAGEMENT SERVICE BY A PHYSICIAN OR OTHER QUALIFIED HEALTH CARE PROFESSIONAL WHO MAY REPORT EVALUATION AND MANAGEMENT SERVICES PROVIDED TO AN ESTABLISHED PATIENT, PARENT, OR GUARDIAN NOT ORIGINATING FROM A RELATED E/M SERVICE PROVIDED WITHIN THE PREVIOUS 7 DAYS NOR LEADING TO AN E/M SERVICE OR PROCEDURE WITHIN THE NEXT 24 HOURS OR SOONEST AVAILABLE APPOINTMENT; 11-20 MINUTES OF MEDICAL DISCUSSION
99443	TELEPHONE EVALUATION AND MANAGEMENT SERVICE BY A PHYSICIAN OR OTHER QUALIFIED HEALTH CARE PROFESSIONAL WHO MAY REPORT EVALUATION AND MANAGEMENT SERVICES PROVIDED TO AN ESTABLISHED PATIENT, PARENT, OR GUARDIAN NOT ORIGINATING FROM A RELATED E/M SERVICE PROVIDED WITHIN THE PREVIOUS 7 DAYS NOR LEADING TO AN E/M SERVICE OR PROCEDURE WITHIN THE NEXT 24 HOURS OR SOONEST AVAILABLE APPOINTMENT; 21-30 MINUTES OF MEDICAL DISCUSSION
99605	MEDICATION THERAPY MANAGEMENT SERVICE(S) PROVIDED BY A PHARMACIST, INDIVIDUAL, FACE-TO-FACE WITH PATIENT, WITH ASSESSMENT AND INTERVENTION IF PROVIDED; INITIAL 15 MINUTES, NEW PATIENT

© 2022 Gujral K et al. *JAMA Network Open.*

99606	MEDICATION THERAPY MANAGEMENT SERVICE(S) PROVIDED BY A PHARMACIST, INDIVIDUAL, FACE-TO-FACE WITH PATIENT, WITH ASSESSMENT AND INTERVENTION IF PROVIDED; INITIAL 15 MINUTES, ESTABLISHED PATIENT
99607	MEDICATION THERAPY MANAGEMENT SERVICE(S) PROVIDED BY A PHARMACIST, INDIVIDUAL, FACE-TO-FACE WITH PATIENT, WITH ASSESSMENT AND INTERVENTION IF PROVIDED; EACH ADDITIONAL 15 MINUTES (LIST SEPARATELY IN ADDITION TO CODE FOR PRIMARY SERVICE)

eTable 6: VA codes for providers and qualified prescribers, used as part of the criteria for identifying medication management visits

**VA Codes for Psychiatrists**		
**VA Code**	**Classification**	**Area of Specialization**
V180506	Pediatrics	Child Abuse Pediatrics
V180701	Family Medicine	Addiction Medicine
V181001	Internal Medicine	Addiction Medicine
V182901	Psychiatry & Neurology	Addiction Medicine
V182902	Psychiatry & Neurology	Addiction Psychiatry
V182903	Psychiatry & Neurology	Child & Adolescent Psychiatry
V182905	Psychiatry & Neurology	Forensic Psychiatry
V182906	Psychiatry & Neurology	Geriatric Psychiatry
V182911	Psychiatry & Neurology	Psychiatry
V182914	Psychiatry & Neurology	Behavioral Neurology & Neuropsychiatry
V182918	Psychiatry & Neurology	Hospice and Palliative Medicine
V182920	Psychiatry & Neurology	Psychosomatic Medicine
V182921	Psychiatry & Neurology	Sleep Medicine
**VA Codes Other Qualified Prescribers**		
**VA Code**	**Classification**	**Area of Specialization**
V070100	Clinical Specialist	NULL
V070102	Clinical Specialist	Child and Adolescent Psychiatric and Mental Health
V070103	Clinical Specialist	Community Health Nursing
V070104	Clinical Specialist	Gerontological Nursing
V070105	Clinical Specialist	Medical-Surgical Nursing
V070500	Nurse Practitioner	NULL
V070501	Nurse Practitioner	Adult Nurse Practitioner
V070502	Nurse Practitioner	Family Nurse Practitioner
V070503	Nurse Practitioner	Gerontological Nurse Practitioner
V070504	Nurse Practitioner	Neonatal Nurse Practitioner
V070505	Nurse Practitioner	Obstetrical/Gynecological Nurse Practitioner
V070506	Nurse Practitioner	Pediatric Nurse Practitioner (PNP)
V070507	Nurse Practitioner	School Nurse Practitioner
V090101	Pharmacist	General Practice
V090102	Pharmacist	Nuclear

© 2022 Gujral K et al. *JAMA Network Open.*

V090102	Pharmacist	Nuclear Pharmacy
V090103	Pharmacist	Nutrition Support
V090104	Pharmacist	Pharmacotherapy
V090105	Pharmacist	Psychiatric
V090105	Pharmacist	Psychopharmacy
V090106	Pharmacist	Pharmacist Clinician (PhC)/ Clinical Pharmacy Specialist
V090107	Pharmacist	Geriatric
V090108	Pharmacist	Oncology
V100000	Physician Assistant	NULL
V100100	Physician Assistant	Medical
V100200	Physician Assistant	Surgical
V100300	Clinical Nurse Specialist	NULL
V100301	Clinical Nurse Specialist	Acute Care
V100302	Clinical Nurse Specialist	Adult Health
V100303	Clinical Nurse Specialist	Chronic Care
V100304	Clinical Nurse Specialist	Community Health/Public Health
V100305	Clinical Nurse Specialist	Critical Care Medicine
V100306	Clinical Nurse Specialist	Emergency
V100307	Clinical Nurse Specialist	Ethics
V100308	Clinical Nurse Specialist	Family Health
V100309	Clinical Nurse Specialist	Gerontology
V100310	Clinical Nurse Specialist	Holistic
V100311	Clinical Nurse Specialist	Home Health
V100312	Clinical Nurse Specialist	Informatics
V100313	Clinical Nurse Specialist	Long-Term Care
V100314	Clinical Nurse Specialist	Medical-Surgical
V100315	Clinical Nurse Specialist	Neonatal
V100316	Clinical Nurse Specialist	Neonatal, High-Risk
V100317	Clinical Nurse Specialist	Neuroscience
V100318	Clinical Nurse Specialist	Occupational Health
V100319	Clinical Nurse Specialist	Oncology
V100320	Clinical Nurse Specialist	Oncology, Pediatrics
V100321	Clinical Nurse Specialist	Pediatrics
V100322	Clinical Nurse Specialist	Perinatal
V100323	Clinical Nurse Specialist	Perioperative
V100324	Clinical Nurse Specialist	Psychiatric/Mental Health
V100325	Clinical Nurse Specialist	Psychiatric/Mental Health, Adult
V100326	Clinical Nurse Specialist	Psychiatric/Mental Health, Child & Adolescent
V100327	Clinical Nurse Specialist	Psychiatric/Mental Health, Child & Family
V100328	Clinical Nurse Specialist	Psychiatric/Mental Health, Chronically Ill
V100329	Clinical Nurse Specialist	Psychiatric/Mental Health, Community

V100330	Clinical Nurse Specialist	Psychiatric/Mental Health, Geropsychiatric
V100331	Clinical Nurse Specialist	Rehabilitation
V100332	Clinical Nurse Specialist	Rural Health
V100333	Clinical Nurse Specialist	School
V100334	Clinical Nurse Specialist	Transplantation
V100335	Clinical Nurse Specialist	Women's Health
V100401	Advanced Practice Midwife	NULL
V100500	Nurse Anesthetist, Certified Registered	NULL
V100600	Nurse Practitioner	NULL
V100601	Nurse Practitioner	NULL
V100601	Nurse Practitioner	Acute Care
V100602	Nurse Practitioner	Adult Health
V100603	Nurse Practitioner	Community Health
V100604	Nurse Practitioner	Critical Care Medicine
V100605	Nurse Practitioner	Family
V100606	Nurse Practitioner	Gerontology
V100607	Nurse Practitioner	Neonatal
V100608	Nurse Practitioner	Neonatal, Critical Care
V100609	Nurse Practitioner	Obstetrics & Gynecology
V100610	Nurse Practitioner	Occupational Health
V100611	Nurse Practitioner	Pediatrics
V100612	Nurse Practitioner	Pediatrics: Acute Care
V100613	Nurse Practitioner	Pediatrics, Critical Care
V100614	Nurse Practitioner	Perinatal
V100615	Nurse Practitioner	Primary Care
V100616	Nurse Practitioner	Psychiatric/Mental Health
V100617	Nurse Practitioner	School
V100618	Nurse Practitioner	Adult Health
V100618	Nurse Practitioner	Women's Health
V100700	Anesthesiologist Assistant	NULL
V110000	Internal Medicine	NULL
V110000	Physician/Osteopath	NULL
V110100	Physician/Osteopath	Addiction Medicine
V110200	Physician/Osteopath	Allergy
V110300	Physician/Osteopath	Allergy & Immunology
V110301	Physician/Osteopath	Immunology, Clinical & Laboratory: Allergy & Immunology
V110400	Physician/Osteopath	Anesthesiology
V110401	Physician/Osteopath	Critical Care Medicine: Anesthesiology
V110402	Physician/Osteopath	Pain Management - Anesthesiology
V110403	Anesthesiology	Pediatric Anesthesiology

© 2022 Gujral K et al. *JAMA Network Open.*

V110403	Physician/Osteopath	Anesthesiology: Pediatric Anesthesiology
V110404	Physician/Osteopath	Pain Management
V110500	Physician/Osteopath	Body Imaging
V110600	Physician/Osteopath	Cardiology
V110700	Physician/Osteopath	Dermatology
V110701	Physician/Osteopath	Immunology, Clinical & Laboratory Dermatological
V110702	Physician/Osteopath	Dermatopathology: Dermatology
V110703	Physician/Osteopath	Dermatology: Pediatric Dermatology
V110800	Physician/Osteopath	Emergency Medicine
V110801	Physician/Osteopath	Toxicology, Medical: Emergency Medicine
V110802	Emergency Medicine	Pediatric Emergency Medicine
V110803	Physician/Osteopath	Sports Medicine: Emergency Medicine
V110804	Physician/Osteopath	Emergency Medicine: Undersea and Hyperbaric Medicine
V110900	Physician/Osteopath	Family Practice
V110901	Physician/Osteopath	Geriatric Medicine: Family Practice
V110902	Physician/Osteopath	Sports Medicine: Family Practice
V111000	Physician/Osteopath	General Practice
V111100	Physician/Osteopath	Geriatric Medicine: General Practice
V111200	Physician/Osteopath	Hematology & Oncology
V111500	Internal Medicine	Gastroenterology
V111500	Physician/Osteopath	Internal Medicine
V111501	Physician/Osteopath	Adolescent Medicine: Internal Medicine
V111502	Physician/Osteopath	Cardiac Electrophysiology
V111503	Physician/Osteopath	Cardiovascular Disease
V111504	Physician/Osteopath	Immunology, Clinical & Laboratory: Internal Medicine
V111505	Physician/Osteopath	Critical Care Medicine: Internal Medicine
V111506	Physician/Osteopath	Endocrinology, Diabetes & Metabolism
V111507	Internal Medicine	Gastroenterology
V111507	Physician/Osteopath	Gastroenterology
V111508	Physician/Osteopath	Geriatric Medicine: Internal Medicine
V111509	Physician/Osteopath	Hematology: Internal Medicine
V111510	Physician/Osteopath	Infectious Diseases
V111511	Physician/Osteopath	Oncology, Medical

© 2022 Gujral K et al. *JAMA Network Open.*

V111512	Physician/Osteopath	Nephrology
V111513	Physician/Osteopath	Pulmonary Diseases
V111514	Physician/Osteopath	Pulmonary Medicine
V111515	Physician/Osteopath	Rheumatology
V111516	Physician/Osteopath	Sports Medicine: Internal Medicine
V111517	Physician/Osteopath	Internal Medicine: Hepatology
V111518	Physician/Osteopath	Internal Medicine: Interventional Cardiology
V111519	Physician/Osteopath	Internal Medicine: Pediatrics
V111520	Physician/Osteopath	Internal Medicine: Peripheral Vascular Disease
V111521	Physician/Osteopath	Internal Medicine: Pulmonary Critical Care Medicine
V111600	Physician/Osteopath	Laboratory Medicine
V111610	Physician/Osteopath	Legal Medicine
V111700	Legal Medicine	NULL
V111800	Physician/Osteopath	Medical Diseases of the Chest
V111900	Physician/Osteopath	Genetics, Medical
V111901	Physician/Osteopath	Genetics, Clinical Biochemical
V111902	Physician/Osteopath	Genetics, Clinical Biochemical/ Molecular
V111903	Physician/Osteopath	Cytogenetics, Clinical
V111904	Physician/Osteopath	Genetics, Clinical (M.D.)
V111905	Physician/Osteopath	Genetics, Clinical Molecular
V111906	Physician/Osteopath	Medical Genetics: Molecular Genetic Pathology
V112000	Physician/Osteopath	Neopathology
V112100	Physician/Osteopath	Neurology
V112101	Physician/Osteopath	Neurological Surgery: Pediatric Neurological Surgery
V112200	Physician/Osteopath	Neurology, Child
V112300	Physician/Osteopath	Neuroradiology
V112400	Physician/Osteopath	Nuclear Cardiology
V112500	Physician/Osteopath	Nuclear Imaging & Therapy
V112600	Physician/Osteopath	Nuclear Medicine
V112601	Physician/Osteopath	Nuclear Medicine, In Vivo & In Vitro
V112800	Physician/Osteopath	Obstetrics
V112900	Physician/Osteopath	Obstetrics & Gynecology
V112901	Physician/Osteopath	Critical Care Medicine: OB/GYN
V112902	Physician/Osteopath	Oncology, Gynecologic
V112903	Physician/Osteopath	Maternal & Fetal Medicine
V112904	Physician/Osteopath	Endocrinology, Reproductive
V113000	Physician/Osteopath	Ophthalmology

V113001	Physician/Osteopath	Opthalmology: Pediatric Opthalmology
V113200	Physician/Osteopath	Osteopathic Manipulative Medicine, Special Proficiency
V113300	Physician/Osteopath	Otolaryngology
V113301	Physician/Osteopath	Otology & Neurotology
V113302	Physician/Osteopath	Pediatric Otolaryngology
V113400	Physician/Osteopath	Otology
V113500	Physician/Osteopath	Otorhinolaryngology
V113600	Physician/Osteopath	Surgery, Otorhinolaryngology & Facial Plastic Surgery
V113700	Physician/Osteopath	Pathology
V113701	Physician/Osteopath	Pathology, Anatomic & Clinical
V113702	Physician/Osteopath	Pathology, Anatomic
V113703	Physician/Osteopath	Pathology, Anatomic & Laboratory Medicine
V113704	Physician/Osteopath	Blood Banking & Transfusion Medicine
V113705	Physician/Osteopath	Pathology, Chemical
V113706	Physician/Osteopath	Pathology, Clinical
V113707	Physician/Osteopath	Cytopathology
V113708	Physician/Osteopath	Dermatopathology
V113709	Physician/Osteopath	Forensic Pathology
V113710	Physician/Osteopath	Hematology: Pathology
V113711	Physician/Osteopath	Immunopathology
V113712	Physician/Osteopath	Medical Microbiology
V113713	Physician/Osteopath	Neuropathology
V113714	Physician/Osteopath	Pediatric Pathology
V113715	Physician/Osteopath	Pathology: Selective Pathology
V113716	Physician/Osteopath	Pathology: Molecular Genetic Pathology
V113800	Physician/Osteopath	Pediatric Allergy & Immunology
V113900	Physician/Osteopath	Pediatric Intensive Care
V114000	Physician/Osteopath	Pediatric Neurology
V114001	Physician/Osteopath	Pediatrics: Neurodevelopmental Disabilities
V114002	Physician/Osteopath	Pediatrics: Developmental - Behavioral Pediatrics
V114100	Physician/Osteopath	Psychiatry, Pediatric
V114200	Physician/Osteopath	Pediatrics
V114201	Physician/Osteopath	Adolescent Medicine: Pediatrics
V114202	Physician/Osteopath	Immunology, Clinical & Laboratory: Pediatric
V114203	Physician/Osteopath	Pediatric Medical Toxocology
V114204	Physician/Osteopath	Neonatal-Perinatal Medicine
V114205	Physician/Osteopath	Pediatric Cardiology

© 2022 Gujral K et al. *JAMA Network Open.*

V114206	Physician/Osteopath	Pediatric Critical Care Medicine
V114207	Physician/Osteopath	Pediatric Emergency Medicine
V114208	Physician/Osteopath	Pediatric Endocrinology
V114209	Physician/Osteopath	Pediatric Gastroenterology
V114210	Physician/Osteopath	Pediatric Hematology Oncology
V114211	Physician/Osteopath	Pediatric Infectious Diseases
V114212	Physician/Osteopath	Pediatric Nephrology
V114213	Physician/Osteopath	Pediatric Pulmonology
V114215	Physician/Osteopath	Pediatric Rheumatology
V114216	Physician/Osteopath	Sports Medicine: Pediatrics
V114300	Physician/Osteopath	Pediatric Radiology
V114400	Physician/Osteopath	Pharmacology, Clinical
V114500	Physician/Osteopath	Physical Medicine & Rehabilitation
V114501	Physician/Osteopath	Physical Medicine & Rehabilitation: Pain Management
V114502	Physician/Osteopath	Physical Medicine & Rehabilitation: Pediatric Rehabilitation Medicine
V114503	Physician/Osteopath	Physical Medicine & Rehabilitation: Sports Medicine
V114504	Physician/Osteopath	Physical Medicine and Rehabilitation: Spinal Cord Injury
V114600	Physician/Osteopath	Preventive Medicine, General
V114601	Physician/Osteopath	Aerospace Medicine: Preventive Medicine
V114602	Physician/Osteopath	Toxicology, Medical: Preventive Medicine
V114603	Physician/Osteopath	Occupational Medicine: Preventive Medicine
V114604	Physician/Osteopath	Occupational-Environmental Medicine: Preventive Medicine
V114605	Physician/Osteopath	Public Health & General Preventive Medicine
V114606	Physician/Osteopath	Underseas Medicine: Preventive Medicine
V114607	Physician/Osteopath	Preventive Medicine: Sports Medicine
V114608	Physician/Osteopath	Preventive Medicine: Undersea and Hyperbaric Medicine
V114700	Physician/Osteopath	Proctology
V114800	Physician/Osteopath	Psychiatry & Neurology
V114801	Physician/Osteopath	Psychiatry, Addiction
V114802	Physician/Osteopath	Psychiatry, Child & Adolescent
V114803	Physician/Osteopath	Neurophysiology, Clinical
V114804	Physician/Osteopath	Forensic Psychiatry
V114805	Physician/Osteopath	Psychiatry, Geriatric

© 2022 Gujral K et al. *JAMA Network Open.*

V114806	Psychiatry and Neurology	Neurology
V114807	Psychiatry and Neurology	Neurology, Child
V114808	Physician/Osteopath	Psychiatry
V114809	Physician/Osteopath	Psychiatry & Neurology: Neurodevelopmental Disabilities
V114810	Physician/Osteopath	Psychiatry & Neurology: Pain Management
V114900	Physician/Osteopath	Psychiatry, Child
V115000	Psychoanalysis	NULL
V115100	Physician/Osteopath	Radiation Therapy
V115200	Physician/Osteopath	Pathology, Radioisotopic
V115300	Physician/Osteopath	Radiology
V115301	Physician/Osteopath	Radiology, Diagnostic
V115302	Physician/Osteopath	Nuclear Radiology
V115303	Radiology	Pediatric Radiology
V115304	Physician/Osteopath	Radiation Oncology
V115305	Physician/Osteopath	Radiological Physics
V115306	Radiology	Radiology
V115307	Physician/Osteopath	Radiology, Vascular & Interventional
V115308	Physician/Osteopath	Radiology: Abdominal Radiology
V115309	Physician/Osteopath	Radiology: Musculoskeletal Radiology
V115400	Physician/Osteopath	Rehabilitation Medicine
V115701	Physician/Osteopath	Surgery, General Vascular
V115703	Physician/Osteopath	Surgery, Pediatric
V115704	Physician/Osteopath	Surgery, Hand
V115705	Physician/Osteopath	Surgical Critical Care: Surgery
V115800	Physician/Osteopath	Surgery, Cardiovascular
V115900	Physician/Osteopath	Surgery, Colon & Rectal Surgery
V116000	Physician/Osteopath	Dermatology Micrographic Surgery
V116100	Physician/Osteopath	Surgery, General
V116200	Physician/Osteopath	Surgery, Head & Neck
V116400	Physician/Osteopath	Surgery, Neurological
V116500	Physician/Osteopath	Surgery, Obstetric & Gynecologic
V116600	Physician/Osteopath	Surgery, Orthopedic
V116700	Physician/Osteopath	Surgery, Orthopedic, Adult Reconstructive
V116800	Physician/Osteopath	Surgery, Hand: Orthopedic Surgery
V116801	Physician/Osteopath	Surgery: Micrographic Surgery
V116900	Physician/Osteopath	Surgery, Orthopedic, Musculoskeletal Oncology
V117000	Physician/Osteopath	Surgery, Orthopedic, Pediatric
V117100	Physician/Osteopath	Surgery, Orthopedic, Spine

© 2022 Gujral K et al. *JAMA Network Open.*

V117300	Physician/Osteopath	Surgery, Orthopedic, Trauma
V117400	Physician/Osteopath	Surgery, Plastic
V117401	Physician/Osteopath	Surgery, Hand: Plastic Surgery
V117402	Physician/Osteopath	Plastic Surgery: Craniofacial Surgery
V117403	Physician/Osteopath	Plastic Surgery: Plastic Surgery Within the Head and Neck
V117500	Physician/Osteopath	Surgery, Plastic & Reconstructive
V117700	Physician/Osteopath	Surgery, Thoracic
V117800	Physician/Osteopath	Surgery, Thoracic Cardiovascular
V117900	Physician/Osteopath	Surgery, Traumatic
V118000	Physician/Osteopath	Surgery, Urological
V118100	Physician/Osteopath	Ultrasound, Diagnostic
V118200	Physician/Osteopath	Urology
V118201	Physician/Osteopath	Urology, Pediatric Urology
V118301	Physician/Osteopath	Adolescent Medicine
V118302	Physician/Osteopath	Adolescent Medicine: Family Practice
V118303	Physician/Osteopath	Aerospace Medicine
V118304	Physician/Osteopath	Allergy & Immunology: Internal Medicine
V118305	Physician/Osteopath	Adolescent Only, Under 16
V118306	Physician/Osteopath	Adolescent Only, Under 21
V118307	Physician/Osteopath	Age Specific, Greater than 1 Year Old
V118308	Physician/Osteopath	Age Specific, Newborns Only
V118309	Physician/Osteopath	Blood Banking
V118310	Physician/Osteopath	Cardiac Electrophysiology, Clinical
V118311	Physician/Osteopath	Critical Care Medicine
V118312	Physician/Osteopath	Diabetes
V118313	Physician/Osteopath	Endocrinology
V118314	Physician/Osteopath	Geriatric Medicine
V118315	Physician/Osteopath	Gynecology
V118316	Physician/Osteopath	Hematology
V118317	Physician/Osteopath	Immunology, Clinical & Laboratory
V118318	Physician/Osteopath	Immunology, Dermatological
V118319	Physician/Osteopath	Immunology: Laboratory, Diagnostic
V118320	Physician/Osteopath	Infertility
V118321	Physician/Osteopath	Neonatology
V118322	Physician/Osteopath	Public Health: Preventive Medicine
V118323	Physician/Osteopath	Pharmacotherapy
V118324	Physician/Osteopath	Psychopharmacy

© 2022 Gujral K et al. *JAMA Network Open.*

V118325	Physician/Osteopath	Pain Medicine
V118326	Physician/Osteopath	Radiology, Angiography & Interventional
V118327	Physician/Osteopath	Radiology, Therapeutic
V118328	Physician/Osteopath	Radium Therapy
V118329	Physician/Osteopath	Rhinology
V118330	Physician/Osteopath	Roentgenology
V118331	Physician/Osteopath	Roentgenology, Diagnostic
V118332	Physician/Osteopath	Sports Medicine
V118333	Physician/Osteopath	Surgery, Abdominal
V118334	Physician/Osteopath	Surgery, Facial Plastic
V118335	Physician/Osteopath	Toxicology, Medical
V118336	Physician/Osteopath	Thermography
V118337	Physician/Osteopath	Occupational Medicine
V118338	Physician/Osteopath	Oncology
V118339	Physician/Osteopath	Otorhinolaryngology & Head-Neck
V118340	Physician/Osteopath	Orthopedic
V118341	Physician/Osteopath	Orthopedic Surgery: Foot and Ankle Orthopedics
V118342	Physician/Osteopath	Orthopedic Surgery: Sports Medicine
V118343	Physician/Osteopath	Sports Medicine - OMM
V160100	Physician/Osteopath (Other Roles)	NULL
V160101	Physician/Osteopath	Laboratory Service Provider
V160102	Physician/Osteopath	Supplier
V170500	Nursing Home Administrator	NULL
V170900	Day Training/Habilitation Specialist	NULL
V180300	Clinical Pharmacology	NULL
V180600	Emergency Medicine	NULL
V180601	Emergency Medicine	Emergency Medical Services
V180602	Emergency Medicine	Medical Toxicology
V180603	Emergency Medicine	Pediatric Emergency Medicine
V180604	Emergency Medicine	Sports Medicine
V180605	Emergency Medicine	Undersea and Hyperbaric Medicine
V180700	Family Medicine	NULL
V180702	Family Medicine	Adolescent Medicine
V180703	Family Medicine	Adult Medicine
V180704	Family Medicine	Geriatric Medicine
V180705	Family Medicine	Sports Medicine
V180707	Family Medicine	Sleep Medicine
V180800	General Practice	NULL

© 2022 Gujral K et al. *JAMA Network Open.*

V181000	Internal Medicine	NULL
V181002	Internal Medicine	Adolescent Medicine
V181003	Internal Medicine	Allergy & Immunology
V181004	Internal Medicine	Cardiovascular Disease
V181005	Internal Medicine	Clinical & Laboratory Immunology
V181006	Internal Medicine	Clinical Cardiac Electrophysiology
V181007	Internal Medicine	Critical Care Medicine
V181008	Internal Medicine	Endocrinology, Diabetes & Metabolism
V181009	Internal Medicine	Gastroenterology
V181010	Internal Medicine	Geriatric Medicine
V181011	Internal Medicine	Hematology
V181012	Internal Medicine	Hematology & Oncology
V181013	Internal Medicine	Hepatology
V181014	Internal Medicine	Infectious Disease
V181015	Internal Medicine	Interventional Cardiology
V181016	Internal Medicine	Magnetic Resonance Imaging (MRI)
V181017	Internal Medicine	Medical Oncology
V181018	Internal Medicine	Nephrology
V181019	Internal Medicine	Pulmonary Disease
V181020	Internal Medicine	Rheumatology
V181021	Internal Medicine	Sports Medicine
V181100	Legal Medicine	NULL
V181550	Electrodiagnostic Medicine	NULL
V182301	Pain Medicine	Interventional Pain Medicine
V182302	Pain Medicine	Pain Medicine
V182401	Pathology	Anatomic Pathology
V182402	Pathology	Anatomic Pathology & Clinical Pathology
V182403	Pathology	Blood Banking & Transfusion Medicine
V182404	Pathology	Chemical Pathology
V182405	Pathology	Clinical Pathology/Laboratory Medicine
V182406	Pathology	Cytopathology
V182407	Pathology	Dermatopathology
V182408	Pathology	Forensic Pathology
V182409	Pathology	Hematology
V182410	Pathology	Immunopathology
V182411	Pathology	Medical Microbiology
V182412	Pathology	Molecular Genetic Pathology
V182413	Pathology	Neuropathology
V182414	Pathology	Pediatric Pathology

© 2022 Gujral K et al. *JAMA Network Open.*

V182600	Physical Medicine & Rehabilitation	NULL
V182601	Physical Medicine & Rehabilitation	Pain Medicine
V182602	Physical Medicine & Rehabilitation	Pediatric Rehabilitation Medicine
V182603	Physical Medicine & Rehabilitation	Spinal Cord Injury Medicine
V182604	Physical Medicine & Rehabilitation	Sports Medicine
V182605	Internal Medicine	Hypertension Specialist
V182605	Physical Medicine & Rehabilitation	Hospice and Palliative Medicine
V182605	Physical Medicine & Rehabilitation	Neuromuscular Medicine
V182801	Preventive Medicine	Aerospace Medicine
V182802	Preventive Medicine	Medical Toxicology
V182803	Preventive Medicine	Occupational Medicine
V182804	Preventive Medicine	Preventive Medicine/Occupational Environmental Medicine
V182805	Preventive Medicine	Public Health & General Preventive Medicine
V182806	Preventive Medicine	Sports Medicine
V182807	Preventive Medicine	Undersea and Hyperbaric Medicine
V182904	Psychiatry & Neurology	Clinical Neurophysiology
V182907	Psychiatry & Neurology	Neurodevelopmental Disabilities
V182908	Psychiatry & Neurology	Neurology
V182909	Psychiatry & Neurology	Neurology with Special Qualifications in Child Neurology
V182910	Psychiatry & Neurology	Pain Medicine
V182912	Psychiatry & Neurology	Sports Medicine
V182913	Psychiatry & Neurology	Vascular Neurology
V183001	Radiology	Body Imaging
V183001	Radiology	Diagnostic Radiology
V183002	Radiology	Diagnostic Radiology
V183003	Radiology	Diagnostic Ultrasound
V183004	Radiology	Diagnostic Radiology
V183004	Radiology	Neuroradiology
V183005	Radiology	Nuclear Radiology
V183006	Radiology	Pediatric Radiology
V183007	Radiology	Radiation Oncology
V183008	Radiology	Radiological Physics
V183009	Radiology	Radiation Oncology
V183009	Radiology	Therapeutic Radiology

© 2022 Gujral K et al. *JAMA Network Open.*

V183010	Radiology	Vascular & Interventional Radiology
V183011	Radiology	Diagnostic Neuroimaging
V183012	Radiology	Hospice and Palliative Medicine
V183100	Surgery	NULL
V183101	Surgery	Pediatric Surgery
V183102	Surgery	Plastic and Reconstructive Surgery
V183103	Surgery	Surgery of the Hand
V183104	Surgery	Surgical Critical Care
V183105	Surgery	Surgical Oncology
V183106	Surgery	Trauma Surgery
V183107	Surgery	Vascular Surgery
V183108	Surgery	Hospice and Palliative Medicine
V183200	Thoracic Surgery (Cardiothoracic Vascular Surgery)	NULL
V183300	Transplant Surgery	NULL
V183400	Urology	NULL
V183401	Urology	Female Pelvic Medicine and Reconstructive Surgery
V183402	Urology	Pediatric Urology

eTable 7: ICD-10 codes for identifying mental health conditions, used as part of the criteria for identifying psychotherapy and medication management visits

**ICD-10 Diagnosis Codes for Mental Health Conditions**					
**ICD10Code**	**ICD10Description**	**Depression**	**PTSD**	**SMI**	**SUD**
F06.31	Mood disorder due to known physiological condition with depressive features	X			
F06.32	Mood disorder due to known physiological condition with major depressive-like episode	X			
F32.	Major depressive disorder, single episode	X			
F32.0	Major depressive disorder, single episode, mild	X			
F32.1	Major depressive disorder, single episode, moderate	X			
F32.2	Major depressive disorder, single episode, severe without psychotic features	X			
F32.3	Major depressive disorder, single episode, severe with psychotic features	X			
F32.4	Major depressive disorder, single episode, in partial remission	X			
F32.5	Major depressive disorder, single episode, in full remission	X			
F32.8	Other Depressive Episodes	X			
F32.81	Premenstrual dysphoric disorder	X			

© 2022 Gujral K et al. *JAMA Network Open.*

F32.89	Other specified depressive episodes	X			
F32.9	Major depressive disorder, single episode, unspecified	X			
F33.	Major depressive disorder, recurrent	X			
F33.0	Major depressive disorder, recurrent, mild	X			
F33.1	Major depressive disorder, recurrent, moderate	X			
F33.2	Major depressive disorder, recurrent severe without psychotic features	X			
F33.3	Major depressive disorder, recurrent, severe with psychotic symptoms	X			
F33.4	Major depressive disorder, recurrent, in remission	X			
F33.40	Major depressive disorder, recurrent, in remission, unspecified	X			
F33.41	Major depressive disorder, recurrent, in partial remission	X			
F33.42	Major depressive disorder, recurrent, in full remission	X			
F33.8	Other recurrent depressive disorders	X			
F33.9	Major depressive disorder, recurrent, unspecified	X			
F34.1	Dysthymic disorder	X			
F43.1	Post-traumatic stress disorder (PTSD)		X		
F43.10	Post-traumatic stress disorder, unspecified		X		
F43.11	Post-traumatic stress disorder, acute		X		
F43.12	Post-traumatic stress disorder, chronic		X		
F06.0	Psychotic disorder with hallucinations due to known physiological condition			X	
F06.2	Psychotic disorder with delusions due to known physiological condition			X	
F20.	Schizophrenia			X	
F20.0	Paranoid schizophrenia			X	
F20.1	Disorganized schizophrenia			X	
F20.2	Catatonic schizophrenia			X	
F20.3	Undifferentiated schizophrenia			X	
F20.5	Residual schizophrenia			X	
F20.8	Other schizophrenia			X	
F20.81	Schizophreniform disorder			X	
F20.89	Other schizophrenia			X	
F20.9	Schizophrenia, unspecified			X	
F22.	Delusional disorders			X	
F23.	Brief psychotic disorder			X	
F24.	Shared psychotic disorder			X	
F25.	Schizoaffective disorders			X	

© 2022 Gujral K et al. *JAMA Network Open.*

F25.0	Schizoaffective disorder, bipolar type			X	
F25.1	Schizoaffective disorder, depressive type			X	
F25.8	Other schizoaffective disorders			X	
F25.9	Schizoaffective disorder, unspecified			X	
F28.	Other psychotic disorder not due to a substance or known physiological condition			X	
F29.	Unspecified psychosis not due to a substance or known physiological condition			X	
F30.	Manic episode			X	
F30.1	Manic episode without psychotic symptoms			X	
F30.10	Manic episode without psychotic symptoms, unspecified			X	
F30.11	Manic episode without psychotic symptoms, mild			X	
F30.12	Manic episode without psychotic symptoms, moderate			X	
F30.13	Manic episode, severe, without psychotic symptoms			X	
F30.2	Manic episode, severe with psychotic symptoms			X	
F30.3	Manic episode in partial remission			X	
F30.4	Manic episode in full remission			X	
F30.8	Other manic episodes			X	
F30.9	Manic episode, unspecified			X	
F31.	Bipolar disorder			X	
F31.0	Bipolar disorder, current episode hypomanic			X	
F31.1	Bipolar disorder, current episode manic without psychotic features			X	
F31.10	Bipolar disorder, current episode manic without psychotic features, unspecified			X	
F31.11	Bipolar disorder, current episode manic without psychotic features, mild			X	
F31.12	Bipolar disorder, current episode manic without psychotic features, moderate			X	
F31.13	Bipolar disorder, current episode manic without psychotic features, severe			X	
F31.2	Bipolar disorder, current episode manic severe with psychotic features			X	
F31.3	Bipolar disorder, current episode depressed, mild or moderate severity			X	
F31.30	Bipolar disorder, current episode depressed, mild or moderate severity, unspecified			X	
F31.31	Bipolar disorder, current episode depressed, mild			X	
F31.32	Bipolar disorder, current episode depressed, moderate			X	

© 2022 Gujral K et al. *JAMA Network Open.*

F31.4	Bipolar disorder, current episode depressed, severe, without psychotic features			X	
F31.5	Bipolar disorder, current episode depressed, severe, with psychotic features			X	
F31.6	Bipolar disorder, current episode mixed			X	
F31.60	Bipolar disorder, current episode mixed, unspecified			X	
F31.61	Bipolar disorder, current episode mixed, mild			X	
F31.62	Bipolar disorder, current episode mixed, moderate			X	
F31.63	Bipolar disorder, current episode mixed, severe, without psychotic features			X	
F31.64	Bipolar disorder, current episode mixed, severe, with psychotic features			X	
F31.7	Bipolar disorder, currently in remission			X	
F31.70	Bipolar disorder, currently in remission, most recent episode unspecified			X	
F31.71	Bipolar disorder, in partial remission, most recent episode hypomanic			X	
F31.72	Bipolar disorder, in full remission, most recent episode hypomanic			X	
F31.73	Bipolar disorder, in partial remission, most recent episode manic			X	
F31.74	Bipolar disorder, in full remission, most recent episode manic			X	
F31.75	Bipolar disorder, in partial remission, most recent episode depressed			X	
F31.76	Bipolar disorder, in full remission, most recent episode depressed			X	
F31.77	Bipolar disorder, in partial remission, most recent episode mixed			X	
F31.78	Bipolar disorder, in full remission, most recent episode mixed			X	
F31.8	Other bipolar disorders			X	
F31.81	Bipolar II disorder			X	
F31.89	Other bipolar disorder			X	
F31.9	Bipolar disorder, unspecified			X	
F53.	Mental and behavioral disorders associated with the puerperium, not elsewhere classified			X	
F53.1	Puerperal psychosis			X	
F10.	Alcohol related disorders				X
F10.1	Alcohol abuse				X
F10.10	Alcohol abuse, uncomplicated				X
F10.11	Alcohol abuse, in remission				X
F10.12	Alcohol abuse with intoxication				X

© 2022 Gujral K et al. *JAMA Network Open.*

F10.120	Alcohol abuse with intoxication, uncomplicated				X
F10.121	Alcohol abuse with intoxication delirium				X
F10.129	Alcohol abuse with intoxication, unspecified				X
F10.13	Alcohol abuse, with withdrawal				X
F10.130	Alcohol abuse with withdrawal, uncomplicated				X
F10.131	Alcohol abuse with withdrawal delirium				X
F10.132	Alcohol abuse with withdrawal with perceptual disturbance				X
F10.139	Alcohol abuse with withdrawal, unspecified				X
F10.14	Alcohol abuse with alcohol-induced mood disorder				X
F10.15	Alcohol abuse with alcohol-induced psychotic disorder				X
F10.150	Alcohol abuse with alcohol-induced psychotic disorder with delusions				X
F10.151	Alcohol abuse with alcohol-induced psychotic disorder with hallucinations				X
F10.159	Alcohol abuse with alcohol-induced psychotic disorder, unspecified				X
F10.18	Alcohol abuse with other alcohol-induced disorders				X
F10.180	Alcohol abuse with alcohol-induced anxiety disorder				X
F10.181	Alcohol abuse with alcohol-induced sexual dysfunction				X
F10.182	Alcohol abuse with alcohol-induced sleep disorder				X
F10.188	Alcohol abuse with other alcohol-induced disorder				X
F10.19	Alcohol abuse with unspecified alcohol-induced disorder				X
F10.2	Alcohol dependence				X
F10.20	Alcohol dependence, uncomplicated				X
F10.21	Alcohol dependence, in remission				X
F10.22	Alcohol dependence with intoxication				X
F10.220	Alcohol dependence with intoxication, uncomplicated				X
F10.221	Alcohol dependence with intoxication delirium				X
F10.229	Alcohol dependence with intoxication, unspecified				X
F10.23	Alcohol dependence with withdrawal				X
F10.230	Alcohol dependence with withdrawal, uncomplicated				X
F10.231	Alcohol dependence with withdrawal delirium				X

© 2022 Gujral K et al. *JAMA Network Open.*

F10.232	Alcohol dependence with withdrawal with perceptual disturbance				X
F10.239	Alcohol dependence with withdrawal, unspecified				X
F10.24	Alcohol dependence with alcohol-induced mood disorder				X
F10.25	Alcohol dependence with alcohol-induced psychotic disorder				X
F10.250	Alcohol dependence with alcohol-induced psychotic disorder with delusions				X
F10.251	Alcohol dependence with alcohol-induced psychotic disorder with hallucinations				X
F10.259	Alcohol dependence with alcohol-induced psychotic disorder, unspecified				X
F10.26	Alcohol dependence with alcohol-induced persisting amnestic disorder				X
F10.27	Alcohol dependence with alcohol-induced persisting dementia				X
F10.28	Alcohol dependence with other alcohol-induced disorders				X
F10.280	Alcohol dependence with alcohol-induced anxiety disorder				X
F10.281	Alcohol dependence with alcohol-induced sexual dysfunction				X
F10.282	Alcohol dependence with alcohol-induced sleep disorder				X
F10.288	Alcohol dependence with other alcohol-induced disorder				X
F10.29	Alcohol dependence with unspecified alcohol-induced disorder				X
F10.9	Alcohol use, unspecified				X
F10.92	Alcohol use, unspecified with intoxication				X
F10.920	Alcohol use, unspecified with intoxication, uncomplicated				X
F10.921	Alcohol use, unspecified with intoxication delirium				X
F10.929	Alcohol use, unspecified with intoxication, unspecified				X
F10.93	Alcohol use, unspecified with withdrawal				X
F10.930	Alcohol use, unspecified with withdrawal, uncomplicated				X
F10.931	Alcohol use, unspecified with withdrawal delirium				X
F10.932	Alcohol use, unspecified with withdrawal with perceptual disturbance				X
F10.939	Alcohol use, unspecified with withdrawal, unspecified				X

© 2022 Gujral K et al. *JAMA Network Open.*

F10.94	Alcohol use, unspecified with alcohol-induced mood disorder				X
F10.95	Alcohol use, unspecified with alcohol-induced psychotic disorder				X
F10.950	Alcohol use, unspecified with alcohol-induced psychotic disorder with delusions				X
F10.951	Alcohol use, unspecified with alcohol-induced psychotic disorder with hallucinations				X
F10.959	Alcohol use, unspecified with alcohol-induced psychotic disorder, unspecified				X
F10.96	Alcohol use, unspecified with alcohol-induced persisting amnestic disorder				X
F10.97	Alcohol use, unspecified with alcohol-induced persisting dementia				X
F10.98	Alcohol use, unspecified with other alcohol-induced disorders				X
F10.980	Alcohol use, unspecified with alcohol-induced anxiety disorder				X
F10.981	Alcohol use, unspecified with alcohol-induced sexual dysfunction				X
F10.982	Alcohol use, unspecified with alcohol-induced sleep disorder				X
F10.988	Alcohol use, unspecified with other alcohol-induced disorder				X
F10.99	Alcohol use, unspecified with unspecified alcohol-induced disorder				X
F11.	Opioid related disorders				X
F11.1	Opioid abuse				X
F11.10	Opioid abuse, uncomplicated				X
F11.11	Opioid abuse, in remission				X
F11.12	Opioid abuse with intoxication				X
F11.120	Opioid abuse with intoxication, uncomplicated				X
F11.121	Opioid abuse with intoxication delirium				X
F11.122	Opioid abuse with intoxication with perceptual disturbance				X
F11.129	Opioid abuse with intoxication, unspecified				X
F11.13	Opioid abuse with withdrawal				X
F11.14	Opioid abuse with opioid-induced mood disorder				X
F11.15	Opioid abuse with opioid-induced psychotic disorder				X
F11.150	Opioid abuse with opioid-induced psychotic disorder with delusions				X
F11.151	Opioid abuse with opioid-induced psychotic disorder with hallucinations				X

© 2022 Gujral K et al. *JAMA Network Open.*

F11.159	Opioid abuse with opioid-induced psychotic disorder, unspecified				X
F11.18	Opioid abuse with other opioid-induced disorder				X
F11.181	Opioid abuse with opioid-induced sexual dysfunction				X
F11.182	Opioid abuse with opioid-induced sleep disorder				X
F11.188	Opioid abuse with other opioid-induced disorder				X
F11.19	Opioid abuse with unspecified opioid-induced disorder				X
F11.2	Opioid dependence				X
F11.20	Opioid dependence, uncomplicated				X
F11.21	Opioid dependence, in remission				X
F11.22	Opioid dependence with intoxication				X
F11.220	Opioid dependence with intoxication, uncomplicated				X
F11.221	Opioid dependence with intoxication delirium				X
F11.222	Opioid dependence with intoxication with perceptual disturbance				X
F11.229	Opioid dependence with intoxication, unspecified				X
F11.23	Opioid dependence with withdrawal				X
F11.24	Opioid dependence with opioid-induced mood disorder				X
F11.25	Opioid dependence with opioid-induced psychotic disorder				X
F11.250	Opioid dependence with opioid-induced psychotic disorder with delusions				X
F11.251	Opioid dependence with opioid-induced psychotic disorder with hallucinations				X
F11.259	Opioid dependence with opioid-induced psychotic disorder, unspecified				X
F11.28	Opioid dependence with other opioid-induced disorder				X
F11.281	Opioid dependence with opioid-induced sexual dysfunction				X
F11.282	Opioid dependence with opioid-induced sleep disorder				X
F11.288	Opioid dependence with other opioid-induced disorder				X
F11.29	Opioid dependence with unspecified opioid-induced disorder				X
F11.9	Opioid use, unspecified				X
F11.90	Opioid use, unspecified, uncomplicated				X

© 2022 Gujral K et al. *JAMA Network Open.*

F11.92	Opioid use, unspecified with intoxication				X
F11.920	Opioid use, unspecified with intoxication, uncomplicated				X
F11.921	Opioid use, unspecified with intoxication delirium				X
F11.922	Opioid use, unspecified with intoxication with perceptual disturbance				X
F11.929	Opioid use, unspecified with intoxication, unspecified				X
F11.93	Opioid use, unspecified with withdrawal				X
F11.94	Opioid use, unspecified with opioid-induced mood disorder				X
F11.95	Opioid use, unspecified with opioid-induced psychotic disorder				X
F11.950	Opioid use, unspecified with opioid-induced psychotic disorder with delusions				X
F11.951	Opioid use, unspecified with opioid-induced psychotic disorder with hallucinations				X
F11.959	Opioid use, unspecified with opioid-induced psychotic disorder, unspecified				X
F11.98	Opioid use, unspecified with other specified opioid-induced disorder				X
F11.981	Opioid use, unspecified with opioid-induced sexual dysfunction				X
F11.982	Opioid use, unspecified with opioid-induced sleep disorder				X
F11.988	Opioid use, unspecified with other opioid-induced disorder				X
F11.99	Opioid use, unspecified with unspecified opioid-induced disorder				X
F12.	Cannabis related disorders				X
F12.1	Cannabis abuse				X
F12.10	Cannabis abuse, uncomplicated				X
F12.11	Cannabis abuse, in remission				X
F12.12	Cannabis abuse with intoxication				X
F12.120	Cannabis abuse with intoxication, uncomplicated				X
F12.121	Cannabis abuse with intoxication delirium				X
F12.122	Cannabis abuse with intoxication with perceptual disturbance				X
F12.129	Cannabis abuse with intoxication, unspecified				X
F12.13	Cannabis abuse with withdrawal				X
F12.15	Cannabis abuse with psychotic disorder				X
F12.150	Cannabis abuse with psychotic disorder with delusions				X

© 2022 Gujral K et al. *JAMA Network Open.*

F12.151	Cannabis abuse with psychotic disorder with hallucinations				X
F12.159	Cannabis abuse with psychotic disorder, unspecified				X
F12.18	Cannabis abuse with other cannabis-induced disorder				X
F12.180	Cannabis abuse with cannabis-induced anxiety disorder				X
F12.188	Cannabis abuse with other cannabis-induced disorder				X
F12.19	Cannabis abuse with unspecified cannabis-induced disorder				X
F12.2	Cannabis dependence				X
F12.20	Cannabis dependence, uncomplicated				X
F12.21	Cannabis dependence, in remission				X
F12.22	Cannabis dependence with intoxication				X
F12.220	Cannabis dependence with intoxication, uncomplicated				X
F12.221	Cannabis dependence with intoxication delirium				X
F12.222	Cannabis dependence with intoxication with perceptual disturbance				X
F12.229	Cannabis dependence with intoxication, unspecified				X
F12.23	Cannabis dependence with withdrawal				X
F12.25	Cannabis dependence with psychotic disorder				X
F12.250	Cannabis dependence with psychotic disorder with delusions				X
F12.251	Cannabis dependence with psychotic disorder with hallucinations				X
F12.259	Cannabis dependence with psychotic disorder, unspecified				X
F12.28	Cannabis dependence with other cannabis-induced disorder				X
F12.280	Cannabis dependence with cannabis-induced anxiety disorder				X
F12.288	Cannabis dependence with other cannabis-induced disorder				X
F12.29	Cannabis dependence with unspecified cannabis-induced disorder				X
F12.9	Cannabis use, unspecified				X
F12.90	Cannabis use, unspecified, uncomplicated				X
F12.92	Cannabis use, unspecified with intoxication				X
F12.920	Cannabis use, unspecified with intoxication, uncomplicated				X

© 2022 Gujral K et al. *JAMA Network Open.*

F12.921	Cannabis use, unspecified with intoxication delirium				X
F12.922	Cannabis use, unspecified with intoxication with perceptual disturbance				X
F12.929	Cannabis use, unspecified with intoxication, unspecified				X
F12.93	Cannabis use, unspecified with withdrawal				X
F12.95	Cannabis use, unspecified with psychotic disorder				X
F12.950	Cannabis use, unspecified with psychotic disorder with delusions				X
F12.951	Cannabis use, unspecified with psychotic disorder with hallucinations				X
F12.959	Cannabis use, unspecified with psychotic disorder, unspecified				X
F12.98	Cannabis use, unspecified with other cannabis-induced disorder				X
F12.980	Cannabis use, unspecified with anxiety disorder				X
F12.988	Cannabis use, unspecified with other cannabis-induced disorder				X
F12.99	Cannabis use, unspecified with unspecified cannabis-induced disorder				X
F13.	Sedative, hypnotic, or anxiolytic related disorders				X
F13.1	Sedative, hypnotic or anxiolytic-related abuse				X
F13.10	Sedative, hypnotic or anxiolytic abuse, uncomplicated				X
F13.11	Sedative, hypnotic or anxiolytic abuse, in remission				X
F13.12	Sedative, hypnotic or anxiolytic abuse with intoxication				X
F13.120	Sedative, hypnotic or anxiolytic abuse with intoxication, uncomplicated				X
F13.121	Sedative, hypnotic or anxiolytic abuse with intoxication delirium				X
F13.129	Sedative, hypnotic or anxiolytic abuse with intoxication, unspecified				X
F13.13	Sedative, hypnotic or anxiolytic abuse with withdrawal				X
F13.130	Sedative, hypnotic or anxiolytic abuse with withdrawal, uncomplicated				X
F13.131	Sedative, hypnotic or anxiolytic abuse with withdrawal delirium				X
F13.132	Sedative, hypnotic or anxiolytic abuse with withdrawal with perceptual disturbance				X

© 2022 Gujral K et al. *JAMA Network Open.*

F13.139	Sedative, hypnotic or anxiolytic abuse with withdrawal, unspecified				X
F13.14	Sedative, hypnotic or anxiolytic abuse with sedative, hypnotic or anxiolytic-induced mood disorder				X
F13.15	Sedative, hypnotic or anxiolytic abuse with sedative, hypnotic or anxiolytic-induced psychotic disorder				X
F13.150	Sedative, hypnotic or anxiolytic abuse with sedative, hypnotic or anxiolytic-induced psychotic disorder with delusions				X
F13.151	Sedative, hypnotic or anxiolytic abuse with sedative, hypnotic or anxiolytic-induced psychotic disorder with hallucinations				X
F13.159	Sedative, hypnotic or anxiolytic abuse with sedative, hypnotic or anxiolytic-induced psychotic disorder, unspecified				X
F13.18	Sedative, hypnotic or anxiolytic abuse with other sedative, hypnotic or anxiolytic-induced disorders				X
F13.180	Sedative, hypnotic or anxiolytic abuse with sedative, hypnotic or anxiolytic-induced anxiety disorder				X
F13.181	Sedative, hypnotic or anxiolytic abuse with sedative, hypnotic or anxiolytic-induced sexual dysfunction				X
F13.182	Sedative, hypnotic or anxiolytic abuse with sedative, hypnotic or anxiolytic-induced sleep disorder				X
F13.188	Sedative, hypnotic or anxiolytic abuse with other sedative, hypnotic or anxiolytic-induced disorder				X
F13.19	Sedative, hypnotic or anxiolytic abuse with unspecified sedative, hypnotic or anxiolytic-induced disorder				X
F13.2	Sedative, hypnotic or anxiolytic-related dependence				X
F13.20	Sedative, hypnotic or anxiolytic dependence, uncomplicated				X
F13.21	Sedative, hypnotic or anxiolytic dependence, in remission				X
F13.22	Sedative, hypnotic or anxiolytic dependence with intoxication				X
F13.220	Sedative, hypnotic or anxiolytic dependence with intoxication, uncomplicated				X
F13.221	Sedative, hypnotic or anxiolytic dependence with intoxication delirium				X

© 2022 Gujral K et al. *JAMA Network Open.*

F13.229	Sedative, hypnotic or anxiolytic dependence with intoxication, unspecified				X
F13.23	Sedative, hypnotic or anxiolytic dependence with withdrawal				X
F13.230	Sedative, hypnotic or anxiolytic dependence with withdrawal, uncomplicated				X
F13.231	Sedative, hypnotic or anxiolytic dependence with withdrawal delirium				X
F13.232	Sedative, hypnotic or anxiolytic dependence with withdrawal with perceptual disturbance				X
F13.239	Sedative, hypnotic or anxiolytic dependence with withdrawal, unspecified				X
F13.24	Sedative, hypnotic or anxiolytic dependence with sedative, hypnotic or anxiolytic-induced mood disorder				X
F13.25	Sedative, hypnotic or anxiolytic dependence with sedative, hypnotic or anxiolytic-induced psychotic disorder				X
F13.250	Sedative, hypnotic or anxiolytic dependence with sedative, hypnotic or anxiolytic-induced psychotic disorder with delusions				X
F13.251	Sedative, hypnotic or anxiolytic dependence with sedative, hypnotic or anxiolytic-induced psychotic disorder with hallucinations				X
F13.259	Sedative, hypnotic or anxiolytic dependence with sedative, hypnotic or anxiolytic-induced psychotic disorder, unspecified				X
F13.26	Sedative, hypnotic or anxiolytic dependence with sedative, hypnotic or anxiolytic-induced persisting amnestic disorder				X
F13.27	Sedative, hypnotic or anxiolytic dependence with sedative, hypnotic or anxiolytic-induced persisting dementia				X
F13.28	Sedative, hypnotic or anxiolytic dependence with other sedative, hypnotic or anxiolytic-induced disorders				X
F13.280	Sedative, hypnotic or anxiolytic dependence with sedative, hypnotic or anxiolytic-induced anxiety disorder				X
F13.281	Sedative, hypnotic or anxiolytic dependence with sedative, hypnotic or anxiolytic-induced sexual dysfunction				X
F13.282	Sedative, hypnotic or anxiolytic dependence with sedative, hypnotic or anxiolytic-induced sleep disorder				X

© 2022 Gujral K et al. *JAMA Network Open.*

F13.288	Sedative, hypnotic or anxiolytic dependence with other sedative, hypnotic or anxiolytic-induced disorder				X
F13.29	Sedative, hypnotic or anxiolytic dependence with unspecified sedative, hypnotic or anxiolytic-induced disorder				X
F13.9	Sedative, hypnotic or anxiolytic-related use, unspecified				X
F13.90	Sedative, hypnotic, or anxiolytic use, unspecified, uncomplicated				X
F13.92	Sedative, hypnotic or anxiolytic use, unspecified with intoxication				X
F13.920	Sedative, hypnotic or anxiolytic use, unspecified with intoxication, uncomplicated				X
F13.921	Sedative, hypnotic or anxiolytic use, unspecified with intoxication delirium				X
F13.929	Sedative, hypnotic or anxiolytic use, unspecified with intoxication, unspecified				X
F13.93	Sedative, hypnotic or anxiolytic use, unspecified with withdrawal				X
F13.930	Sedative, hypnotic or anxiolytic use, unspecified with withdrawal, uncomplicated				X
F13.931	Sedative, hypnotic or anxiolytic use, unspecified with withdrawal delirium				X
F13.932	Sedative, hypnotic or anxiolytic use, unspecified with withdrawal with perceptual disturbances				X
F13.939	Sedative, hypnotic or anxiolytic use, unspecified with withdrawal, unspecified				X
F13.94	Sedative, hypnotic or anxiolytic use, unspecified with sedative, hypnotic or anxiolytic-induced mood disorder				X
F13.95	Sedative, hypnotic or anxiolytic use, unspecified with sedative, hypnotic or anxiolytic-induced psychotic disorder				X
F13.950	Sedative, hypnotic or anxiolytic use, unspecified with sedative, hypnotic or anxiolytic-induced psychotic disorder with delusions				X
F13.951	Sedative, hypnotic or anxiolytic use, unspecified with sedative, hypnotic or anxiolytic-induced psychotic disorder with hallucinations				X
F13.959	Sedative, hypnotic or anxiolytic use, unspecified with sedative, hypnotic or anxiolytic-induced psychotic disorder, unspecified				X

© 2022 Gujral K et al. *JAMA Network Open.*

F13.96	Sedative, hypnotic or anxiolytic use, unspecified with sedative, hypnotic or anxiolytic-induced persisting amnestic disorder				X
F13.97	Sedative, hypnotic or anxiolytic use, unspecified with sedative, hypnotic or anxiolytic-induced persisting dementia				X
F13.98	Sedative, hypnotic or anxiolytic use, unspecified with other sedative, hypnotic or anxiolytic-induced disorders				X
F13.980	Sedative, hypnotic or anxiolytic use, unspecified with sedative, hypnotic or anxiolytic-induced anxiety disorder				X
F13.981	Sedative, hypnotic or anxiolytic use, unspecified with sedative, hypnotic or anxiolytic-induced sexual dysfunction				X
F13.982	Sedative, hypnotic or anxiolytic use, unspecified with sedative, hypnotic or anxiolytic-induced sleep disorder				X
F13.988	Sedative, hypnotic or anxiolytic use, unspecified with other sedative, hypnotic or anxiolytic-induced disorder				X
F13.99	Sedative, hypnotic or anxiolytic use, unspecified with unspecified sedative, hypnotic or anxiolytic-induced disorder				X
F14.	Cocaine related disorders				X
F14.1	Cocaine abuse				X
F14.10	Cocaine abuse, uncomplicated				X
F14.11	Cocaine abuse, in remission				X
F14.12	Cocaine abuse with intoxication				X
F14.120	Cocaine abuse with intoxication, uncomplicated				X
F14.121	Cocaine abuse with intoxication with delirium				X
F14.122	Cocaine abuse with intoxication with perceptual disturbance				X
F14.129	Cocaine abuse with intoxication, unspecified				X
F14.13	Cocaine abuse, unspecified with withdrawal				X
F14.14	Cocaine abuse with cocaine-induced mood disorder				X
F14.15	Cocaine abuse with cocaine-induced psychotic disorder				X
F14.150	Cocaine abuse with cocaine-induced psychotic disorder with delusions				X
F14.151	Cocaine abuse with cocaine-induced psychotic disorder with hallucinations				X
F14.159	Cocaine abuse with cocaine-induced psychotic disorder, unspecified				X

© 2022 Gujral K et al. *JAMA Network Open.*

F14.18	Cocaine abuse with other cocaine-induced disorder				X
F14.180	Cocaine abuse with cocaine-induced anxiety disorder				X
F14.181	Cocaine abuse with cocaine-induced sexual dysfunction				X
F14.182	Cocaine abuse with cocaine-induced sleep disorder				X
F14.188	Cocaine abuse with other cocaine-induced disorder				X
F14.19	Cocaine abuse with unspecified cocaine-induced disorder				X
F14.2	Cocaine dependence				X
F14.20	Cocaine dependence, uncomplicated				X
F14.21	Cocaine dependence, in remission				X
F14.22	Cocaine dependence with intoxication				X
F14.220	Cocaine dependence with intoxication, uncomplicated				X
F14.221	Cocaine dependence with intoxication delirium				X
F14.222	Cocaine dependence with intoxication with perceptual disturbance				X
F14.229	Cocaine dependence with intoxication, unspecified				X
F14.23	Cocaine dependence with withdrawal				X
F14.24	Cocaine dependence with cocaine-induced mood disorder				X
F14.25	Cocaine dependence with cocaine-induced psychotic disorder				X
F14.250	Cocaine dependence with cocaine-induced psychotic disorder with delusions				X
F14.251	Cocaine dependence with cocaine-induced psychotic disorder with hallucinations				X
F14.259	Cocaine dependence with cocaine-induced psychotic disorder, unspecified				X
F14.28	Cocaine dependence with other cocaine-induced disorder				X
F14.280	Cocaine dependence with cocaine-induced anxiety disorder				X
F14.281	Cocaine dependence with cocaine-induced sexual dysfunction				X
F14.282	Cocaine dependence with cocaine-induced sleep disorder				X
F14.288	Cocaine dependence with other cocaine-induced disorder				X
F14.29	Cocaine dependence with unspecified cocaine-induced disorder				X

© 2022 Gujral K et al. *JAMA Network Open.*

F14.9	Cocaine use, unspecified				X
F14.90	Cocaine use, unspecified, uncomplicated				X
F14.92	Cocaine use, unspecified with intoxication				X
F14.920	Cocaine use, unspecified with intoxication, uncomplicated				X
F14.921	Cocaine use, unspecified with intoxication delirium				X
F14.922	Cocaine use, unspecified with intoxication with perceptual disturbance				X
F14.929	Cocaine use, unspecified with intoxication, unspecified				X
F14.93	Cocaine use, unspecified with withdrawal				X
F14.94	Cocaine use, unspecified with cocaine-induced mood disorder				X
F14.95	Cocaine use, unspecified with cocaine-induced psychotic disorder				X
F14.950	Cocaine use, unspecified with cocaine-induced psychotic disorder with delusions				X
F14.951	Cocaine use, unspecified with cocaine-induced psychotic disorder with hallucinations				X
F14.959	Cocaine use, unspecified with cocaine-induced psychotic disorder, unspecified				X
F14.98	Cocaine use, unspecified with other specified cocaine-induced disorder				X
F14.980	Cocaine use, unspecified with cocaine-induced anxiety disorder				X
F14.981	Cocaine use, unspecified with cocaine-induced sexual dysfunction				X
F14.982	Cocaine use, unspecified with cocaine-induced sleep disorder				X
F14.988	Cocaine use, unspecified with other cocaine-induced disorder				X
F14.99	Cocaine use, unspecified with unspecified cocaine-induced disorder				X
F15.	Other stimulant related disorders				X
F15.1	Other stimulant abuse				X
F15.10	Other stimulant abuse, uncomplicated				X
F15.11	Other stimulant abuse, in remission				X
F15.12	Other stimulant abuse with intoxication				X
F15.120	Other stimulant abuse with intoxication, uncomplicated				X
F15.121	Other stimulant abuse with intoxication delirium				X
F15.122	Other stimulant abuse with intoxication with perceptual disturbance				X

© 2022 Gujral K et al. *JAMA Network Open.*

F15.129	Other stimulant abuse with intoxication, unspecified				X
F15.13	Other stimulant abuse with withdrawal				X
F15.14	Other stimulant abuse with stimulant-induced mood disorder				X
F15.15	Other stimulant abuse with stimulant-induced psychotic disorder				X
F15.150	Other stimulant abuse with stimulant-induced psychotic disorder with delusions				X
F15.151	Other stimulant abuse with stimulant-induced psychotic disorder with hallucinations				X
F15.159	Other stimulant abuse with stimulant-induced psychotic disorder, unspecified				X
F15.18	Other stimulant abuse with other stimulant-induced disorder				X
F15.180	Other stimulant abuse with stimulant-induced anxiety disorder				X
F15.181	Other stimulant abuse with stimulant-induced sexual dysfunction				X
F15.182	Other stimulant abuse with stimulant-induced sleep disorder				X
F15.188	Other stimulant abuse with other stimulant-induced disorder				X
F15.19	Other stimulant abuse with unspecified stimulant-induced disorder				X
F15.2	Other stimulant dependence				X
F15.20	Other stimulant dependence, uncomplicated				X
F15.21	Other stimulant dependence, in remission				X
F15.22	Other stimulant dependence with intoxication				X
F15.220	Other stimulant dependence with intoxication, uncomplicated				X
F15.221	Other stimulant dependence with intoxication delirium				X
F15.222	Other stimulant dependence with intoxication with perceptual disturbance				X
F15.229	Other stimulant dependence with intoxication, unspecified				X
F15.23	Other stimulant dependence with withdrawal				X
F15.24	Other stimulant dependence with stimulant-induced mood disorder				X
F15.25	Other stimulant dependence with stimulant-induced psychotic disorder				X
F15.250	Other stimulant dependence with stimulant-induced psychotic disorder with delusions				X
F15.251	Other stimulant dependence with stimulant-induced psychotic disorder with hallucinations				X

© 2022 Gujral K et al. *JAMA Network Open.*

F15.259	Other stimulant dependence with stimulant-induced psychotic disorder, unspecified				X
F15.28	Other stimulant dependence with other stimulant-induced disorder				X
F15.280	Other stimulant dependence with stimulant-induced anxiety disorder				X
F15.281	Other stimulant dependence with stimulant-induced sexual dysfunction				X
F15.282	Other stimulant dependence with stimulant-induced sleep disorder				X
F15.288	Other stimulant dependence with other stimulant-induced disorder				X
F15.29	Other stimulant dependence with unspecified stimulant-induced disorder				X
F15.9	Other stimulant use, unspecified				X
F15.90	Other stimulant use, unspecified, uncomplicated				X
F15.92	Other stimulant use, unspecified with intoxication				X
F15.920	Other stimulant use, unspecified with intoxication, uncomplicated				X
F15.921	Other stimulant use, unspecified with intoxication delirium				X
F15.922	Other stimulant use, unspecified with intoxication with perceptual disturbance				X
F15.929	Other stimulant use, unspecified with intoxication, unspecified				X
F15.93	Other stimulant use, unspecified with withdrawal				X
F15.94	Other stimulant use, unspecified with stimulant-induced mood disorder				X
F15.95	Other stimulant use, unspecified with stimulant-induced psychotic disorder				X
F15.950	Other stimulant use, unspecified with stimulant-induced psychotic disorder with delusions				X
F15.951	Other stimulant use, unspecified with stimulant-induced psychotic disorder with hallucinations				X
F15.959	Other stimulant use, unspecified with stimulant-induced psychotic disorder, unspecified				X
F15.98	Other stimulant use, unspecified with other stimulant-induced disorder				X
F15.980	Other stimulant use, unspecified with stimulant-induced anxiety disorder				X

© 2022 Gujral K et al. *JAMA Network Open.*

F15.981	Other stimulant use, unspecified with stimulant-induced sexual dysfunction				X
F15.982	Other stimulant use, unspecified with stimulant-induced sleep disorder				X
F15.988	Other stimulant use, unspecified with other stimulant-induced disorder				X
F15.99	Other stimulant use, unspecified with unspecified stimulant-induced disorder				X
F16.	Hallucinogen related disorders				X
F16.1	Hallucinogen abuse				X
F16.10	Hallucinogen abuse, uncomplicated				X
F16.11	Hallucinogen abuse, in remission				X
F16.12	Hallucinogen abuse with intoxication				X
F16.120	Hallucinogen abuse with intoxication, uncomplicated				X
F16.121	Hallucinogen abuse with intoxication with delirium				X
F16.122	Hallucinogen abuse with intoxication with perceptual disturbance				X
F16.129	Hallucinogen abuse with intoxication, unspecified				X
F16.14	Hallucinogen abuse with hallucinogen-induced mood disorder				X
F16.15	Hallucinogen abuse with hallucinogen-induced psychotic disorder				X
F16.150	Hallucinogen abuse with hallucinogen-induced psychotic disorder with delusions				X
F16.151	Hallucinogen abuse with hallucinogen-induced psychotic disorder with hallucinations				X
F16.159	Hallucinogen abuse with hallucinogen-induced psychotic disorder, unspecified				X
F16.18	Hallucinogen abuse with other hallucinogen-induced disorder				X
F16.180	Hallucinogen abuse with hallucinogen-induced anxiety disorder				X
F16.183	Hallucinogen abuse with hallucinogen persisting perception disorder (flashbacks)				X
F16.188	Hallucinogen abuse with other hallucinogen-induced disorder				X
F16.19	Hallucinogen abuse with unspecified hallucinogen-induced disorder				X
F16.2	Hallucinogen dependence				X
F16.20	Hallucinogen dependence, uncomplicated				X
F16.21	Hallucinogen dependence, in remission				X
F16.22	Hallucinogen dependence with intoxication				X

© 2022 Gujral K et al. *JAMA Network Open.*

F16.220	Hallucinogen dependence with intoxication, uncomplicated				X
F16.221	Hallucinogen dependence with intoxication with delirium				X
F16.229	Hallucinogen dependence with intoxication, unspecified				X
F16.24	Hallucinogen dependence with hallucinogen-induced mood disorder				X
F16.25	Hallucinogen dependence with hallucinogen-induced psychotic disorder				X
F16.250	Hallucinogen dependence with hallucinogen-induced psychotic disorder with delusions				X
F16.251	Hallucinogen dependence with hallucinogen-induced psychotic disorder with hallucinations				X
F16.259	Hallucinogen dependence with hallucinogen-induced psychotic disorder, unspecified				X
F16.28	Hallucinogen dependence with other hallucinogen-induced disorder				X
F16.280	Hallucinogen dependence with hallucinogen-induced anxiety disorder				X
F16.283	Hallucinogen dependence with hallucinogen persisting perception disorder (flashbacks)				X
F16.288	Hallucinogen dependence with other hallucinogen-induced disorder				X
F16.29	Hallucinogen dependence with unspecified hallucinogen-induced disorder				X
F16.9	Hallucinogen use, unspecified				X
F16.90	Hallucinogen use, unspecified, uncomplicated				X
F16.92	Hallucinogen use, unspecified with intoxication				X
F16.920	Hallucinogen use, unspecified with intoxication, uncomplicated				X
F16.921	Hallucinogen use, unspecified with intoxication with delirium				X
F16.929	Hallucinogen use, unspecified with intoxication, unspecified				X
F16.94	Hallucinogen use, unspecified with hallucinogen-induced mood disorder				X
F16.95	Hallucinogen use, unspecified with hallucinogen-induced psychotic disorder				X
F16.950	Hallucinogen use, unspecified with hallucinogen-induced psychotic disorder with delusions				X
F16.951	Hallucinogen use, unspecified with hallucinogen-induced psychotic disorder with hallucinations				X

© 2022 Gujral K et al. *JAMA Network Open.*

F16.959	Hallucinogen use, unspecified with hallucinogen-induced psychotic disorder, unspecified				X
F16.98	Hallucinogen use, unspecified with other specified hallucinogen-induced disorder				X
F16.980	Hallucinogen use, unspecified with hallucinogen-induced anxiety disorder				X
F16.983	Hallucinogen use, unspecified with hallucinogen persisting perception disorder (flashbacks)				X
F16.988	Hallucinogen use, unspecified with other hallucinogen-induced disorder				X
F16.99	Hallucinogen use, unspecified with unspecified hallucinogen-induced disorder				X
F18.	Inhalant related disorders				X
F18.1	Inhalant abuse				X
F18.10	Inhalant abuse, uncomplicated				X
F18.11	Inhalant abuse, in remission				X
F18.12	Inhalant abuse with intoxication				X
F18.120	Inhalant abuse with intoxication, uncomplicated				X
F18.121	Inhalant abuse with intoxication delirium				X
F18.129	Inhalant abuse with intoxication, unspecified				X
F18.14	Inhalant abuse with inhalant-induced mood disorder				X
F18.15	Inhalant abuse with inhalant-induced psychotic disorder				X
F18.150	Inhalant abuse with inhalant-induced psychotic disorder with delusions				X
F18.151	Inhalant abuse with inhalant-induced psychotic disorder with hallucinations				X
F18.159	Inhalant abuse with inhalant-induced psychotic disorder, unspecified				X
F18.17	Inhalant abuse with inhalant-induced dementia				X
F18.18	Inhalant abuse with other inhalant-induced disorders				X
F18.180	Inhalant abuse with inhalant-induced anxiety disorder				X
F18.188	Inhalant abuse with other inhalant-induced disorder				X
F18.19	Inhalant abuse with unspecified inhalant-induced disorder				X
F18.2	Inhalant dependence				X
F18.20	Inhalant dependence, uncomplicated				X
F18.21	Inhalant dependence, in remission				X

© 2022 Gujral K et al. *JAMA Network Open.*

F18.22	Inhalant dependence with intoxication				X
F18.220	Inhalant dependence with intoxication, uncomplicated				X
F18.221	Inhalant dependence with intoxication delirium				X
F18.229	Inhalant dependence with intoxication, unspecified				X
F18.24	Inhalant dependence with inhalant-induced mood disorder				X
F18.25	Inhalant dependence with inhalant-induced psychotic disorder				X
F18.250	Inhalant dependence with inhalant-induced psychotic disorder with delusions				X
F18.251	Inhalant dependence with inhalant-induced psychotic disorder with hallucinations				X
F18.259	Inhalant dependence with inhalant-induced psychotic disorder, unspecified				X
F18.27	Inhalant dependence with inhalant-induced dementia				X
F18.28	Inhalant dependence with other inhalant-induced disorders				X
F18.280	Inhalant dependence with inhalant-induced anxiety disorder				X
F18.288	Inhalant dependence with other inhalant-induced disorder				X
F18.29	Inhalant dependence with unspecified inhalant-induced disorder				X
F18.9	Inhalant use, unspecified				X
F18.90	Inhalant use, unspecified, uncomplicated				X
F18.92	Inhalant use, unspecified with intoxication				X
F18.920	Inhalant use, unspecified with intoxication, uncomplicated				X
F18.921	Inhalant use, unspecified with intoxication with delirium				X
F18.929	Inhalant use, unspecified with intoxication, unspecified				X
F18.94	Inhalant use, unspecified with inhalant-induced mood disorder				X
F18.95	Inhalant use, unspecified with inhalant-induced psychotic disorder				X
F18.950	Inhalant use, unspecified with inhalant-induced psychotic disorder with delusions				X
F18.951	Inhalant use, unspecified with inhalant-induced psychotic disorder with hallucinations				X
F18.959	Inhalant use, unspecified with inhalant-induced psychotic disorder, unspecified				X

© 2022 Gujral K et al. *JAMA Network Open.*

F18.97	Inhalant use, unspecified with inhalant-induced persisting dementia				X
F18.98	Inhalant use, unspecified with other inhalant-induced disorders				X
F18.980	Inhalant use, unspecified with inhalant-induced anxiety disorder				X
F18.988	Inhalant use, unspecified with other inhalant-induced disorder				X
F18.99	Inhalant use, unspecified with unspecified inhalant-induced disorder				X
F19.	Other psychoactive substance related disorders				X
F19.1	Other psychoactive substance abuse				X
F19.10	Other psychoactive substance abuse, uncomplicated				X
F19.11	Other psychoactive substance abuse, in remission				X
F19.12	Other psychoactive substance abuse with intoxication				X
F19.120	Other psychoactive substance abuse with intoxication, uncomplicated				X
F19.121	Other psychoactive substance abuse with intoxication delirium				X
F19.122	Other psychoactive substance abuse with intoxication with perceptual disturbances				X
F19.129	Other psychoactive substance abuse with intoxication, unspecified				X
F19.13	Other psychoactive substance abuse with withdrawal				X
F19.130	Other psychoactive substance abuse with withdrawal, uncomplicated				X
F19.131	Other psychoactive substance abuse with withdrawal delirium				X
F19.132	Other psychoactive substance abuse with withdrawal with perceptual disturbance				X
F19.139	Other psychoactive substance abuse with withdrawal, unspecified				X
F19.14	Other psychoactive substance abuse with psychoactive substance-induced mood disorder				X
F19.15	Other psychoactive substance abuse with psychoactive substance-induced psychotic disorder				X
F19.150	Other psychoactive substance abuse with psychoactive substance-induced psychotic disorder with delusions				X

© 2022 Gujral K et al. *JAMA Network Open.*

F19.151	Other psychoactive substance abuse with psychoactive substance-induced psychotic disorder with hallucinations				X
F19.159	Other psychoactive substance abuse with psychoactive substance-induced psychotic disorder, unspecified				X
F19.16	Other psychoactive substance abuse with psychoactive substance-induced persisting amnestic disorder				X
F19.17	Other psychoactive substance abuse with psychoactive substance-induced persisting dementia				X
F19.18	Other psychoactive substance abuse with other psychoactive substance-induced disorders				X
F19.180	Other psychoactive substance abuse with psychoactive substance-induced anxiety disorder				X
F19.181	Other psychoactive substance abuse with psychoactive substance-induced sexual dysfunction				X
F19.182	Other psychoactive substance abuse with psychoactive substance-induced sleep disorder				X
F19.188	Other psychoactive substance abuse with other psychoactive substance-induced disorder				X
F19.19	Other psychoactive substance abuse with unspecified psychoactive substance-induced disorder				X
F19.2	Other psychoactive substance dependence				X
F19.20	Other psychoactive substance dependence, uncomplicated				X
F19.21	Other psychoactive substance dependence, in remission				X
F19.22	Other psychoactive substance dependence with intoxication				X
F19.220	Other psychoactive substance dependence with intoxication, uncomplicated				X
F19.221	Other psychoactive substance dependence with intoxication delirium				X
F19.222	Other psychoactive substance dependence with intoxication with perceptual disturbance				X
F19.229	Other psychoactive substance dependence with intoxication, unspecified				X
F19.23	Other psychoactive substance dependence with withdrawal				X
F19.230	Other psychoactive substance dependence with withdrawal, uncomplicated				X

© 2022 Gujral K et al. *JAMA Network Open.*

F19.231	Other psychoactive substance dependence with withdrawal delirium				X
F19.232	Other psychoactive substance dependence with withdrawal with perceptual disturbance				X
F19.239	Other psychoactive substance dependence with withdrawal, unspecified				X
F19.24	Other psychoactive substance dependence with psychoactive substance-induced mood disorder				X
F19.25	Other psychoactive substance dependence with psychoactive substance-induced psychotic disorder				X
F19.250	Other psychoactive substance dependence with psychoactive substance-induced psychotic disorder with delusions				X
F19.251	Other psychoactive substance dependence with psychoactive substance-induced psychotic disorder with hallucinations				X
F19.259	Other psychoactive substance dependence with psychoactive substance-induced psychotic disorder, unspecified				X
F19.26	Other psychoactive substance dependence with psychoactive substance-induced persisting amnestic disorder				X
F19.27	Other psychoactive substance dependence with psychoactive substance-induced persisting dementia				X
F19.28	Other psychoactive substance dependence with other psychoactive substance-induced disorders				X
F19.280	Other psychoactive substance dependence with psychoactive substance-induced anxiety disorder				X
F19.281	Other psychoactive substance dependence with psychoactive substance-induced sexual dysfunction				X
F19.282	Other psychoactive substance dependence with psychoactive substance-induced sleep disorder				X
F19.288	Other psychoactive substance dependence with other psychoactive substance-induced disorder				X
F19.29	Other psychoactive substance dependence with unspecified psychoactive substance-induced disorder				X
F19.9	Other psychoactive substance use, unspecified				X

© 2022 Gujral K et al. *JAMA Network Open.*

F19.90	Other psychoactive substance use, unspecified, uncomplicated				X
F19.92	Other psychoactive substance use, unspecified with intoxication				X
F19.920	Other psychoactive substance use, unspecified with intoxication, uncomplicated				X
F19.921	Other psychoactive substance use, unspecified with intoxication with delirium				X
F19.922	Other psychoactive substance use, unspecified with intoxication with perceptual disturbance				X
F19.929	Other psychoactive substance use, unspecified with intoxication, unspecified				X
F19.93	Other psychoactive substance use, unspecified with withdrawal				X
F19.930	Other psychoactive substance use, unspecified with withdrawal, uncomplicated				X
F19.931	Other psychoactive substance use, unspecified with withdrawal delirium				X
F19.932	Other psychoactive substance use, unspecified with withdrawal with perceptual disturbance				X
F19.939	Other psychoactive substance use, unspecified with withdrawal, unspecified				X
F19.94	Other psychoactive substance use, unspecified with psychoactive substance-induced mood disorder				X
F19.95	Other psychoactive substance use, unspecified with psychoactive substance-induced psychotic disorder				X
F19.950	Other psychoactive substance use, unspecified with psychoactive substance-induced psychotic disorder with delusions				X
F19.951	Other psychoactive substance use, unspecified with psychoactive substance-induced psychotic disorder with hallucinations				X
F19.959	Other psychoactive substance use, unspecified with psychoactive substance-induced psychotic disorder, unspecified				X
F19.96	Other psychoactive substance use, unspecified with psychoactive substance-induced persisting amnestic disorder				X
F19.97	Other psychoactive substance use, unspecified with psychoactive substance-induced persisting dementia				X
F19.98	Other psychoactive substance use, unspecified with other psychoactive substance-induced disorders				X

© 2022 Gujral K et al. *JAMA Network Open.*

F19.980	Other psychoactive substance use, unspecified with psychoactive substance-induced anxiety disorder				X
F19.981	Other psychoactive substance use, unspecified with psychoactive substance-induced sexual dysfunction				X
F19.982	Other psychoactive substance use, unspecified with psychoactive substance-induced sleep disorder				X
F19.988	Other psychoactive substance use, unspecified with other psychoactive substance-induced disorder				X
F19.99	Other psychoactive substance use, unspecified with unspecified psychoactive substance-induced disorder				X
F06.30	Mood disorder due to known physiological condition, unspecified				
F06.33	Mood disorder due to known physiological condition with manic features				
F06.34	Mood disorder due to known physiological condition with mixed features				
F06.4	Anxiety disorder due to known physiological condition				
F21.	Schizotypal disorder				
F34.	Persistent mood [affective] disorders				
F34.0	Cyclothymic disorder				
F34.8	Other Persistent Mood [Affective] Disorders				
F34.81	Disruptive mood dysregulation disorder				
F34.89	Other specified persistent mood disorders				
F34.9	Persistent mood [affective] disorder, unspecified				
F39.	Unspecified mood [affective] disorder				
F40.	Phobic anxiety disorders				
F40.0	Agoraphobia				
F40.00	Agoraphobia, unspecified				
F40.01	Agoraphobia with panic disorder				
F40.02	Agoraphobia without panic disorder				
F40.1	Social phobias				
F40.10	Social phobia, unspecified				
F40.11	Social phobia, generalized				
F40.2	Specific (isolated) phobias				
F40.21	Animal type phobia				
F40.210	Arachnophobia				
F40.218	Other animal type phobia				
F40.22	Natural environment type phobia				

F40.220	Fear of thunderstorms				
F40.228	Other natural environment type phobia				
F40.23	Blood, injection, injury type phobia				
F40.230	Fear of blood				
F40.231	Fear of injections and transfusions				
F40.232	Fear of other medical care				
F40.233	Fear of injury				
F40.24	Situational type phobia				
F40.240	Claustrophobia				
F40.241	Acrophobia				
F40.242	Fear of bridges				
F40.243	Fear of flying				
F40.248	Other situational type phobia				
F40.29	Other specified phobia				
F40.290	Androphobia				
F40.291	Gynephobia				
F40.298	Other specified phobia				
F40.8	Other phobic anxiety disorders				
F40.9	Phobic anxiety disorder, unspecified				
F41.0	Panic disorder [episodic paroxysmal anxiety]				
F41.1	Generalized anxiety disorder				
F41.3	Other mixed anxiety disorders				
F41.8	Other specified anxiety disorders				
F41.9	Anxiety disorder, unspecified				
F42.	Obsessive-Compulsive Disorder				
F42.2	Mixed obsessional thoughts and acts				
F42.3	Hoarding disorder				
F42.4	Excoriation (skin-picking) disorder				
F42.8	Other obsessive-compulsive disorder				
F42.9	Obsessive-compulsive disorder, unspecified				
F43.0	Acute stress reaction				
F43.2	Adjustment disorders				
F43.20	Adjustment disorder, unspecified				
F43.21	Adjustment disorder with depressed mood				
F43.22	Adjustment disorder with anxiety				
F43.23	Adjustment disorder with mixed anxiety and depressed mood				
F43.24	Adjustment disorder with disturbance of conduct				
F43.25	Adjustment disorder with mixed disturbance of emotions and conduct				
F43.29	Adjustment disorder with other symptoms				
F43.8	Other reactions to severe stress				

© 2022 Gujral K et al. *JAMA Network Open.*

F43.9	Reaction to severe stress, unspecified				
F44.	Dissociative and conversion disorders				
F44.0	Dissociative amnesia				
F44.1	Dissociative fugue				
F44.2	Dissociative stupor				
F44.4	Conversion disorder with motor symptom or deficit				
F44.5	Conversion disorder with seizures or convulsions				
F44.6	Conversion disorder with sensory symptom or deficit				
F44.7	Conversion disorder with mixed symptom presentation				
F44.8	Other dissociative and conversion disorders				
F44.81	Dissociative identity disorder				
F44.89	Other dissociative and conversion disorders				
F44.9	Dissociative and conversion disorder, unspecified				
F45.0	Somatization disorder				
F45.1	Undifferentiated somatoform disorder				
F45.20	Hypochondriacal disorder, unspecified				
F45.21	Hypochondriasis				
F45.22	Body dysmorphic disorder				
F45.29	Other hypochondriacal disorders				
F45.8	Other somatoform disorders				
F45.9	Somatoform disorder, unspecified				
F48.1	Depersonalization-derealization syndrome				
F50.	Eating disorders				
F50.0	Anorexia nervosa				
F50.00	Anorexia nervosa, unspecified				
F50.01	Anorexia nervosa, restricting type				
F50.02	Anorexia nervosa, binge eating/purging type				
F50.2	Bulimia nervosa				
F50.8	Other Eating Disorders				
F50.81	Binge eating disorder				
F50.82	Avoidant/restrictive food intake disorder				
F50.89	Other specified eating disorder				
F50.9	Eating disorder, unspecified				
F53.0	Postpartum depression				
F60.	Specific personality disorders				
F60.0	Paranoid personality disorder				
F60.1	Schizoid personality disorder				
F60.2	Antisocial personality disorder				
F60.3	Borderline personality disorder				

© 2022 Gujral K et al. *JAMA Network Open.*

F60.4	Histrionic personality disorder				
F60.5	Obsessive-compulsive personality disorder				
F60.6	Avoidant personality disorder				
F60.7	Dependent personality disorder				
F60.8	Other specific personality disorders				
F60.81	Narcissistic personality disorder				
F60.89	Other specific personality disorders				
F60.9	Personality disorder, unspecified				
F63.	Impulse disorders				
F63.0	Pathological gambling				
F63.1	Pyromania				
F63.2	Kleptomania				
F63.3	Trichotillomania				
F63.8	Other impulse disorders				
F63.81	Intermittent explosive disorder				
F63.89	Other impulse disorders				
F63.9	Impulse disorder, unspecified				
F68.1	Factitious disorder				
F68.10	Factitious disorder imposed on self, unspecified				
F68.11	Factitious disorder imposed on self, with predominantly psychological signs and symptoms				
F68.12	Factitious disorder imposed on self, with predominantly physical signs and symptoms				
F68.13	Factitious disorder imposed on self, with combined psychological and physical signs and symptoms				
F68.8	Other specified disorders of adult personality and behavior				
F69.	Unspecified disorder of adult personality and behavior				
F90.	Attention-deficit hyperactivity disorders				
F90.0	Attention-deficit hyperactivity disorder, predominantly inattentive type				
F90.1	Attention-deficit hyperactivity disorder, predominantly hyperactive type				
F90.2	Attention-deficit hyperactivity disorder, combined type				
F90.8	Attention-deficit hyperactivity disorder, other type				
F90.9	Attention-deficit hyperactivity disorder, unspecified type				
F91.	Conduct disorders				
F91.0	Conduct disorder confined to family context				

© 2022 Gujral K et al. *JAMA Network Open.*

F91.1	Conduct disorder, childhood-onset type				
F91.2	Conduct disorder, adolescent-onset type				
F91.3	Oppositional defiant disorder				
F91.8	Other conduct disorders				
F91.9	Conduct disorder, unspecified				
R45.7	State of emotional shock and stress, unspecified				

eTable 8: ICD-10 codes used for identifying suicide-related ED visits

ICD-10 Code	ICD-10 Code Description
T36.0X2A	Poisoning by penicillins, intentional self-harm, initial encounter
T36.0X2D	Poisoning by penicillins, intentional self-harm, subsequent encounter
T36.0X2S	Poisoning by penicillins, intentional self-harm, sequela
T36.1X2A	Poisoning by cephalosporins and other beta-lactam antibiotics, intentional self-harm, initial encounter
T36.1X2D	Poisoning by cephalosporins and other beta-lactam antibiotics, intentional self-harm, subsequent encounter
T36.1X2S	Poisoning by cephalosporins and other beta-lactam antibiotics, intentional self-harm, sequela
T36.2X2A	Poisoning by chloramphenicol group, intentional self-harm, initial encounter
T36.2X2D	Poisoning by chloramphenicol group, intentional self-harm, subsequent encounter
T36.2X2S	Poisoning by chloramphenicol group, intentional self-harm, sequela
T36.3X2A	Poisoning by macrolides, intentional self-harm, initial encounter
T36.3X2D	Poisoning by macrolides, intentional self-harm, subsequent encounter
T36.3X2S	Poisoning by macrolides, intentional self-harm, sequela
T36.4X2A	Poisoning by tetracyclines, intentional self-harm, initial encounter
T36.4X2D	Poisoning by tetracyclines, intentional self-harm, subsequent encounter
T36.4X2S	Poisoning by tetracyclines, intentional self-harm, sequela
T36.5X2A	Poisoning by aminoglycosides, intentional self-harm, initial encounter
T36.5X2D	Poisoning by aminoglycosides, intentional self-harm, subsequent encounter
T36.5X2S	Poisoning by aminoglycosides, intentional self-harm, sequela
T36.6X2A	Poisoning by rifampicins, intentional self-harm, initial encounter
T36.6X2D	Poisoning by rifampicins, intentional self-harm, subsequent encounter
T36.6X2S	Poisoning by rifampicins, intentional self-harm, sequela
T36.7X2A	Poisoning by antifungal antibiotics, systemically used, intentional self-harm, initial encounter
T36.7X2D	Poisoning by antifungal antibiotics, systemically used, intentional self-harm, subsequent encounter
T36.7X2S	Poisoning by antifungal antibiotics, systemically used, intentional self-harm, sequela
T36.8X2A	Poisoning by other systemic antibiotics, intentional self-harm, initial encounter
T36.8X2D	Poisoning by other systemic antibiotics, intentional self-harm, subsequent encounter
T36.8X2S	Poisoning by other systemic antibiotics, intentional self-harm, sequela

© 2022 Gujral K et al. *JAMA Network Open.*

T36.92XA	Poisoning by unspecified systemic antibiotic, intentional self-harm, initial encounter
T36.92XD	Poisoning by unspecified systemic antibiotic, intentional self-harm, subsequent encounter
T36.92XS	Poisoning by unspecified systemic antibiotic, intentional self-harm, sequela
T37.0X2A	Poisoning by sulfonamides, intentional self-harm, initial encounter
T37.0X2D	Poisoning by sulfonamides, intentional self-harm, subsequent encounter
T37.0X2S	Poisoning by sulfonamides, intentional self-harm, sequela
T37.1X2A	Poisoning by antimycobacterial drugs, intentional self-harm, initial encounter
T37.1X2D	Poisoning by antimycobacterial drugs, intentional self-harm, subsequent encounter
T37.1X2S	Poisoning by antimycobacterial drugs, intentional self-harm, sequela
T37.2X2A	Poisoning by antimalarials and drugs acting on other blood protozoa, intentional self-harm, initial encounter
T37.2X2D	Poisoning by antimalarials and drugs acting on other blood protozoa, intentional self-harm, subsequent encounter
T37.2X2S	Poisoning by antimalarials and drugs acting on other blood protozoa, intentional self-harm, sequela
T37.3X2A	Poisoning by other antiprotozoal drugs, intentional self-harm, initial encounter
T37.3X2D	Poisoning by other antiprotozoal drugs, intentional self-harm, subsequent encounter
T37.3X2S	Poisoning by other antiprotozoal drugs, intentional self-harm, sequela
T37.4X2A	Poisoning by anthelminthics, intentional self-harm, initial encounter
T37.4X2D	Poisoning by anthelminthics, intentional self-harm, subsequent encounter
T37.4X2S	Poisoning by anthelminthics, intentional self-harm, sequela
T37.5X2A	Poisoning by antiviral drugs, intentional self-harm, initial encounter
T37.5X2D	Poisoning by antiviral drugs, intentional self-harm, subsequent encounter
T37.5X2S	Poisoning by antiviral drugs, intentional self-harm, sequela
T37.8X2A	Poisoning by other specified systemic anti-infectives and antiparasitics, intentional self-harm, initial encounter
T37.8X2D	Poisoning by other specified systemic anti-infectives and antiparasitics, intentional self-harm, subsequent encounter
T37.8X2S	Poisoning by other specified systemic anti-infectives and antiparasitics, intentional self-harm, sequela
T37.92XA	Poisoning by unspecified systemic anti-infective and antiparasitics, intentional self-harm, initial encounter
T37.92XD	Poisoning by unspecified systemic anti-infective and antiparasitics, intentional self-harm, subsequent encounter
T37.92XS	Poisoning by unspecified systemic anti-infective and antiparasitics, intentional self-harm, sequela
T38.0X2A	Poisoning by glucocorticoids and synthetic analogues, intentional self-harm, initial encounter
T38.0X2D	Poisoning by glucocorticoids and synthetic analogues, intentional self-harm, subsequent encounter
T38.0X2S	Poisoning by glucocorticoids and synthetic analogues, intentional self-harm, sequela
T38.1X2A	Poisoning by thyroid hormones and substitutes, intentional self-harm, initial encounter
T38.1X2D	Poisoning by thyroid hormones and substitutes, intentional self-harm, subsequent encounter
T38.1X2S	Poisoning by thyroid hormones and substitutes, intentional self-harm, sequela

T38.2X2A	Poisoning by antithyroid drugs, intentional self-harm, initial encounter
T38.2X2D	Poisoning by antithyroid drugs, intentional self-harm, subsequent encounter
T38.2X2S	Poisoning by antithyroid drugs, intentional self-harm, sequela
T38.3X2A	Poisoning by insulin and oral hypoglycemic [antidiabetic] drugs, intentional self-harm, initial encounter
T38.3X2D	Poisoning by insulin and oral hypoglycemic [antidiabetic] drugs, intentional self-harm, subsequent encounter
T38.3X2S	Poisoning by insulin and oral hypoglycemic [antidiabetic] drugs, intentional self-harm, sequela
T38.4X2A	Poisoning by oral contraceptives, intentional self-harm, initial encounter
T38.4X2D	Poisoning by oral contraceptives, intentional self-harm, subsequent encounter
T38.4X2S	Poisoning by oral contraceptives, intentional self-harm, sequela
T38.5X2A	Poisoning by other estrogens and progestogens, intentional self-harm, initial encounter
T38.5X2D	Poisoning by other estrogens and progestogens, intentional self-harm, subsequent encounter
T38.5X2S	Poisoning by other estrogens and progestogens, intentional self-harm, sequela
T38.6X2A	Poisoning by antigonadotrophins, antiestrogens, antiandrogens, not elsewhere classified, intentional self-harm, initial encounter
T38.6X2D	Poisoning by antigonadotrophins, antiestrogens, antiandrogens, not elsewhere classified, intentional self-harm, subsequent encounter
T38.6X2S	Poisoning by antigonadotrophins, antiestrogens, antiandrogens, not elsewhere classified, intentional self-harm, sequela
T38.7X2A	Poisoning by androgens and anabolic congeners, intentional self-harm, initial encounter
T38.7X2D	Poisoning by androgens and anabolic congeners, intentional self-harm, subsequent encounter
T38.7X2S	Poisoning by androgens and anabolic congeners, intentional self-harm, sequela
T38.802A	Poisoning by unspecified hormones and synthetic substitutes, intentional self-harm, initial encounter
T38.802D	Poisoning by unspecified hormones and synthetic substitutes, intentional self-harm, subsequent encounter
T38.802S	Poisoning by unspecified hormones and synthetic substitutes, intentional self-harm, sequela
T38.812A	Poisoning by anterior pituitary [adenohypophyseal] hormones, intentional self-harm, initial encounter
T38.812D	Poisoning by anterior pituitary [adenohypophyseal] hormones, intentional self-harm, subsequent encounter
T38.812S	Poisoning by anterior pituitary [adenohypophyseal] hormones, intentional self-harm, sequela
T38.892A	Poisoning by other hormones and synthetic substitutes, intentional self-harm, initial encounter
T38.892D	Poisoning by other hormones and synthetic substitutes, intentional self-harm, subsequent encounter
T38.892S	Poisoning by other hormones and synthetic substitutes, intentional self-harm, sequela
T38.902A	Poisoning by unspecified hormone antagonists, intentional self-harm, initial encounter
T38.902D	Poisoning by unspecified hormone antagonists, intentional self-harm, subsequent encounter

© 2022 Gujral K et al. *JAMA Network Open.*

T38.902S	Poisoning by unspecified hormone antagonists, intentional self-harm, sequela
T38.992A	Poisoning by other hormone antagonists, intentional self-harm, initial encounter
T38.992D	Poisoning by other hormone antagonists, intentional self-harm, subsequent encounter
T38.992S	Poisoning by other hormone antagonists, intentional self-harm, sequela
T39.012A	Poisoning by aspirin, intentional self-harm, initial encounter
T39.012D	Poisoning by aspirin, intentional self-harm, subsequent encounter
T39.012S	Poisoning by aspirin, intentional self-harm, sequela
T39.092A	Poisoning by salicylates, intentional self-harm, initial encounter
T39.092D	Poisoning by salicylates, intentional self-harm, subsequent encounter
T39.092S	Poisoning by salicylates, intentional self-harm, sequela
T39.1X2A	Poisoning by 4-Aminophenol derivatives, intentional self-harm, initial encounter
T39.1X2D	Poisoning by 4-Aminophenol derivatives, intentional self-harm, subsequent encounter
T39.1X2S	Poisoning by 4-Aminophenol derivatives, intentional self-harm, sequela
T39.2X2A	Poisoning by pyrazolone derivatives, intentional self-harm, initial encounter
T39.2X2D	Poisoning by pyrazolone derivatives, intentional self-harm, subsequent encounter
T39.2X2S	Poisoning by pyrazolone derivatives, intentional self-harm, sequela
T39.312A	Poisoning by propionic acid derivatives, intentional self-harm, initial encounter
T39.312D	Poisoning by propionic acid derivatives, intentional self-harm, subsequent encounter
T39.312S	Poisoning by propionic acid derivatives, intentional self-harm, sequela
T39.392A	Poisoning by other nonsteroidal anti-inflammatory drugs [NSAID], intentional self-harm, initial encounter
T39.392D	Poisoning by other nonsteroidal anti-inflammatory drugs [NSAID], intentional self-harm, subsequent encounter
T39.392S	Poisoning by other nonsteroidal anti-inflammatory drugs [NSAID], intentional self-harm, sequela
T39.4X2A	Poisoning by antirheumatics, not elsewhere classified, intentional self-harm, initial encounter
T39.4X2D	Poisoning by antirheumatics, not elsewhere classified, intentional self-harm, subsequent encounter
T39.4X2S	Poisoning by antirheumatics, not elsewhere classified, intentional self-harm, sequela
T39.8X2A	Poisoning by other nonopioid analgesics and antipyretics, not elsewhere classified, intentional self-harm, initial encounter
T39.8X2D	Poisoning by other nonopioid analgesics and antipyretics, not elsewhere classified, intentional self-harm, subsequent encounter
T39.8X2S	Poisoning by other nonopioid analgesics and antipyretics, not elsewhere classified, intentional self-harm, sequela
T39.92XA	Poisoning by unspecified nonopioid analgesic, antipyretic and antirheumatic, intentional self-harm, initial encounter
T39.92XD	Poisoning by unspecified nonopioid analgesic, antipyretic and antirheumatic, intentional self-harm, subsequent encounter
T39.92XS	Poisoning by unspecified nonopioid analgesic, antipyretic and antirheumatic, intentional self-harm, sequela
T40.0X2A	Poisoning by opium, intentional self-harm, initial encounter
T40.0X2D	Poisoning by opium, intentional self-harm, subsequent encounter
T40.0X2S	Poisoning by opium, intentional self-harm, sequela

© 2022 Gujral K et al. *JAMA Network Open.*

T40.1X2A	Poisoning by heroin, intentional self-harm, initial encounter
T40.1X2D	Poisoning by heroin, intentional self-harm, subsequent encounter
T40.1X2S	Poisoning by heroin, intentional self-harm, sequela
T40.2X2A	Poisoning by other opioids, intentional self-harm, initial encounter
T40.2X2D	Poisoning by other opioids, intentional self-harm, subsequent encounter
T40.2X2S	Poisoning by other opioids, intentional self-harm, sequela
T40.3X2A	Poisoning by methadone, intentional self-harm, initial encounter
T40.3X2D	Poisoning by methadone, intentional self-harm, subsequent encounter
T40.3X2S	Poisoning by methadone, intentional self-harm, sequela
T40.412A	Poisoning by fentanyl or fentanyl analogs, intentional self-harm, initial encounter
T40.412D	Poisoning by fentanyl or fentanyl analogs, intentional self-harm, subsequent encounter
T40.412S	Poisoning by fentanyl or fentanyl analogs, intentional self-harm, sequela
T40.422A	Poisoning by tramadol, intentional self-harm, initial encounter
T40.422D	Poisoning by tramadol, intentional self-harm, subsequent encounter
T40.422S	Poisoning by tramadol, intentional self-harm, sequela
T40.492A	Poisoning by other synthetic narcotics, intentional self-harm, initial encounter
T40.492D	Poisoning by other synthetic narcotics, intentional self-harm, subsequent encounter
T40.492S	Poisoning by other synthetic narcotics, intentional self-harm, sequela
T40.4X2A	Poisoning by other synthetic narcotics, intentional self-harm, initial encounter
T40.4X2D	Poisoning by other synthetic narcotics, intentional self-harm, subsequent encounter
T40.4X2S	Poisoning by other synthetic narcotics, intentional self-harm, sequela
T40.5X2A	Poisoning by cocaine, intentional self-harm, initial encounter
T40.5X2D	Poisoning by cocaine, intentional self-harm, subsequent encounter
T40.5X2S	Poisoning by cocaine, intentional self-harm, sequela
T40.602A	Poisoning by unspecified narcotics, intentional self-harm, initial encounter
T40.602D	Poisoning by unspecified narcotics, intentional self-harm, subsequent encounter
T40.602S	Poisoning by unspecified narcotics, intentional self-harm, sequela
T40.692A	Poisoning by other narcotics, intentional self-harm, initial encounter
T40.692D	Poisoning by other narcotics, intentional self-harm, subsequent encounter
T40.692S	Poisoning by other narcotics, intentional self-harm, sequela
T40.7X2A	Poisoning by cannabis (derivatives), intentional self-harm, initial encounter
T40.7X2D	Poisoning by cannabis (derivatives), intentional self-harm, subsequent encounter
T40.7X2S	Poisoning by cannabis (derivatives), intentional self-harm, sequela
T40.8X2A	Poisoning by lysergide [LSD], intentional self-harm, initial encounter
T40.8X2D	Poisoning by lysergide [LSD], intentional self-harm, subsequent encounter
T40.8X2S	Poisoning by lysergide [LSD], intentional self-harm, sequela
T40.902A	Poisoning by unspecified psychodysleptics [hallucinogens], intentional self-harm, initial encounter
T40.902D	Poisoning by unspecified psychodysleptics [hallucinogens], intentional self-harm, subsequent encounter
T40.902S	Poisoning by unspecified psychodysleptics [hallucinogens], intentional self-harm, sequela
T40.992A	Poisoning by other psychodysleptics [hallucinogens], intentional self-harm, initial encounter

© 2022 Gujral K et al. *JAMA Network Open.*

T40.992D	Poisoning by other psychodysleptics [hallucinogens], intentional self-harm, subsequent encounter
T40.992S	Poisoning by other psychodysleptics [hallucinogens], intentional self-harm, sequela
T41.0X2A	Poisoning by inhaled anesthetics, intentional self-harm, initial encounter
T41.0X2D	Poisoning by inhaled anesthetics, intentional self-harm, subsequent encounter
T41.0X2S	Poisoning by inhaled anesthetics, intentional self-harm, sequela
T41.1X2A	Poisoning by intravenous anesthetics, intentional self-harm, initial encounter
T41.1X2D	Poisoning by intravenous anesthetics, intentional self-harm, subsequent encounter
T41.1X2S	Poisoning by intravenous anesthetics, intentional self-harm, sequela
T41.202A	Poisoning by unspecified general anesthetics, intentional self-harm, initial encounter
T41.202D	Poisoning by unspecified general anesthetics, intentional self-harm, subsequent encounter
T41.202S	Poisoning by unspecified general anesthetics, intentional self-harm, sequela
T41.292A	Poisoning by other general anesthetics, intentional self-harm, initial encounter
T41.292D	Poisoning by other general anesthetics, intentional self-harm, subsequent encounter
T41.292S	Poisoning by other general anesthetics, intentional self-harm, sequela
T41.3X2A	Poisoning by local anesthetics, intentional self-harm, initial encounter
T41.3X2D	Poisoning by local anesthetics, intentional self-harm, subsequent encounter
T41.3X2S	Poisoning by local anesthetics, intentional self-harm, sequela
T41.42XA	Poisoning by unspecified anesthetic, intentional self-harm, initial encounter
T41.42XD	Poisoning by unspecified anesthetic, intentional self-harm, subsequent encounter
T41.42XS	Poisoning by unspecified anesthetic, intentional self-harm, sequela
T41.5X2A	Poisoning by therapeutic gases, intentional self-harm, initial encounter
T41.5X2D	Poisoning by therapeutic gases, intentional self-harm, subsequent encounter
T41.5X2S	Poisoning by therapeutic gases, intentional self-harm, sequela
T42.0X2A	Poisoning by hydantoin derivatives, intentional self-harm, initial encounter
T42.0X2D	Poisoning by hydantoin derivatives, intentional self-harm, subsequent encounter
T42.0X2S	Poisoning by hydantoin derivatives, intentional self-harm, sequela
T42.1X2A	Poisoning by iminostilbenes, intentional self-harm, initial encounter
T42.1X2D	Poisoning by iminostilbenes, intentional self-harm, subsequent encounter
T42.1X2S	Poisoning by iminostilbenes, intentional self-harm, sequela
T42.2X2A	Poisoning by succinimides and oxazolidinediones, intentional self-harm, initial encounter
T42.2X2D	Poisoning by succinimides and oxazolidinediones, intentional self-harm, subsequent encounter
T42.2X2S	Poisoning by succinimides and oxazolidinediones, intentional self-harm, sequela
T42.3X2A	Poisoning by barbiturates, intentional self-harm, initial encounter
T42.3X2D	Poisoning by barbiturates, intentional self-harm, subsequent encounter
T42.3X2S	Poisoning by barbiturates, intentional self-harm, sequela
T42.4X2A	Poisoning by benzodiazepines, intentional self-harm, initial encounter
T42.4X2D	Poisoning by benzodiazepines, intentional self-harm, subsequent encounter
T42.4X2S	Poisoning by benzodiazepines, intentional self-harm, sequela
T42.5X2A	Poisoning by mixed antiepileptics, intentional self-harm, initial encounter
T42.5X2D	Poisoning by mixed antiepileptics, intentional self-harm, subsequent encounter
T42.5X2S	Poisoning by mixed antiepileptics, intentional self-harm, sequela

© 2022 Gujral K et al. *JAMA Network Open.*

T42.6X2A	Poisoning by other antiepileptic and sedative-hypnotic drugs, intentional self-harm, initial encounter
T42.6X2D	Poisoning by other antiepileptic and sedative-hypnotic drugs, intentional self-harm, subsequent encounter
T42.6X2S	Poisoning by other antiepileptic and sedative-hypnotic drugs, intentional self-harm, sequela
T42.72XA	Poisoning by unspecified antiepileptic and sedative-hypnotic drugs, intentional self-harm, initial encounter
T42.72XD	Poisoning by unspecified antiepileptic and sedative-hypnotic drugs, intentional self-harm, subsequent encounter
T42.72XS	Poisoning by unspecified antiepileptic and sedative-hypnotic drugs, intentional self-harm, sequela
T42.8X2A	Poisoning by antiparkinsonism drugs and other central muscle-tone depressants, intentional self-harm, initial encounter
T42.8X2D	Poisoning by antiparkinsonism drugs and other central muscle-tone depressants, intentional self-harm, subsequent encounter
T42.8X2S	Poisoning by antiparkinsonism drugs and other central muscle-tone depressants, intentional self-harm, sequela
T43.012A	Poisoning by tricyclic antidepressants, intentional self-harm, initial encounter
T43.012D	Poisoning by tricyclic antidepressants, intentional self-harm, subsequent encounter
T43.012S	Poisoning by tricyclic antidepressants, intentional self-harm, sequela
T43.022A	Poisoning by tetracyclic antidepressants, intentional self-harm, initial encounter
T43.022D	Poisoning by tetracyclic antidepressants, intentional self-harm, subsequent encounter
T43.022S	Poisoning by tetracyclic antidepressants, intentional self-harm, sequela
T43.1X2A	Poisoning by monoamine-oxidase-inhibitor antidepressants, intentional self-harm, initial encounter
T43.1X2D	Poisoning by monoamine-oxidase-inhibitor antidepressants, intentional self-harm, subsequent encounter
T43.1X2S	Poisoning by monoamine-oxidase-inhibitor antidepressants, intentional self-harm, sequela
T43.202A	Poisoning by unspecified antidepressants, intentional self-harm, initial encounter
T43.202D	Poisoning by unspecified antidepressants, intentional self-harm, subsequent encounter
T43.202S	Poisoning by unspecified antidepressants, intentional self-harm, sequela
T43.212A	Poisoning by selective serotonin and norepinephrine reuptake inhibitors, intentional self-harm, initial encounter
T43.212D	Poisoning by selective serotonin and norepinephrine reuptake inhibitors, intentional self-harm, subsequent encounter
T43.212S	Poisoning by selective serotonin and norepinephrine reuptake inhibitors, intentional self-harm, sequela
T43.222A	Poisoning by selective serotonin reuptake inhibitors, intentional self-harm, initial encounter
T43.222D	Poisoning by selective serotonin reuptake inhibitors, intentional self-harm, subsequent encounter
T43.222S	Poisoning by selective serotonin reuptake inhibitors, intentional self-harm, sequela
T43.292A	Poisoning by other antidepressants, intentional self-harm, initial encounter
T43.292D	Poisoning by other antidepressants, intentional self-harm, subsequent encounter

© 2022 Gujral K et al. *JAMA Network Open.*

T43.292S	Poisoning by other antidepressants, intentional self-harm, sequela
T43.3X2A	Poisoning by phenothiazine antipsychotics and neuroleptics, intentional self-harm, initial encounter
T43.3X2D	Poisoning by phenothiazine antipsychotics and neuroleptics, intentional self-harm, subsequent encounter
T43.3X2S	Poisoning by phenothiazine antipsychotics and neuroleptics, intentional self-harm, sequela
T43.4X2A	Poisoning by butyrophenone and thiothixene neuroleptics, intentional self-harm, initial encounter
T43.4X2D	Poisoning by butyrophenone and thiothixene neuroleptics, intentional self-harm, subsequent encounter
T43.4X2S	Poisoning by butyrophenone and thiothixene neuroleptics, intentional self-harm, sequela
T43.502A	Poisoning by unspecified antipsychotics and neuroleptics, intentional self-harm, initial encounter
T43.502D	Poisoning by unspecified antipsychotics and neuroleptics, intentional self-harm, subsequent encounter
T43.502S	Poisoning by unspecified antipsychotics and neuroleptics, intentional self-harm, sequela
T43.592A	Poisoning by other antipsychotics and neuroleptics, intentional self-harm, initial encounter
T43.592D	Poisoning by other antipsychotics and neuroleptics, intentional self-harm, subsequent encounter
T43.592S	Poisoning by other antipsychotics and neuroleptics, intentional self-harm, sequela
T43.602A	Poisoning by unspecified psychostimulants, intentional self-harm, initial encounter
T43.602D	Poisoning by unspecified psychostimulants, intentional self-harm, subsequent encounter
T43.602S	Poisoning by unspecified psychostimulants, intentional self-harm, sequela
T43.612A	Poisoning by caffeine, intentional self-harm, initial encounter
T43.612D	Poisoning by caffeine, intentional self-harm, subsequent encounter
T43.612S	Poisoning by caffeine, intentional self-harm, sequela
T43.622A	Poisoning by amphetamines, intentional self-harm, initial encounter
T43.622D	Poisoning by amphetamines, intentional self-harm, subsequent encounter
T43.622S	Poisoning by amphetamines, intentional self-harm, sequela
T43.632A	Poisoning by methylphenidate, intentional self-harm, initial encounter
T43.632D	Poisoning by methylphenidate, intentional self-harm, subsequent encounter
T43.632S	Poisoning by methylphenidate, intentional self-harm, sequela
T43.642A	Poisoning by ecstasy, intentional self-harm, initial encounter
T43.642D	Poisoning by ecstasy, intentional self-harm, subsequent encounter
T43.642S	Poisoning by ecstasy, intentional self-harm, sequela
T43.692A	Poisoning by other psychostimulants, intentional self-harm, initial encounter
T43.692D	Poisoning by other psychostimulants, intentional self-harm, subsequent encounter
T43.692S	Poisoning by other psychostimulants, intentional self-harm, sequela
T43.8X2A	Poisoning by other psychotropic drugs, intentional self-harm, initial encounter
T43.8X2D	Poisoning by other psychotropic drugs, intentional self-harm, subsequent encounter
T43.8X2S	Poisoning by other psychotropic drugs, intentional self-harm, sequela
T43.92XA	Poisoning by unspecified psychotropic drug, intentional self-harm, initial encounter
T43.92XD	Poisoning by unspecified psychotropic drug, intentional self-harm, subsequent encounter
T43.92XS	Poisoning by unspecified psychotropic drug, intentional self-harm, sequela

© 2022 Gujral K et al. *JAMA Network Open.*

T44.0X2A	Poisoning by anticholinesterase agents, intentional self-harm, initial encounter
T44.0X2D	Poisoning by anticholinesterase agents, intentional self-harm, subsequent encounter
T44.0X2S	Poisoning by anticholinesterase agents, intentional self-harm, sequela
T44.1X2A	Poisoning by other parasympathomimetics [cholinergics], intentional self-harm, initial encounter
T44.1X2D	Poisoning by other parasympathomimetics [cholinergics], intentional self-harm, subsequent encounter
T44.1X2S	Poisoning by other parasympathomimetics [cholinergics], intentional self-harm, sequela
T44.2X2A	Poisoning by ganglionic blocking drugs, intentional self-harm, initial encounter
T44.2X2D	Poisoning by ganglionic blocking drugs, intentional self-harm, subsequent encounter
T44.2X2S	Poisoning by ganglionic blocking drugs, intentional self-harm, sequela
T44.3X2A	Poisoning by other parasympatholytics [anticholinergics and antimuscarinics] and spasmolytics, intentional self-harm, initial encounter
T44.3X2D	Poisoning by other parasympatholytics [anticholinergics and antimuscarinics] and spasmolytics, intentional self-harm, subsequent encounter
T44.3X2S	Poisoning by other parasympatholytics [anticholinergics and antimuscarinics] and spasmolytics, intentional self-harm, sequela
T44.4X2A	Poisoning by predominantly alpha-adrenoreceptor agonists, intentional self-harm, initial encounter
T44.4X2D	Poisoning by predominantly alpha-adrenoreceptor agonists, intentional self-harm, subsequent encounter
T44.4X2S	Poisoning by predominantly alpha-adrenoreceptor agonists, intentional self-harm, sequela
T44.5X2A	Poisoning by predominantly beta-adrenoreceptor agonists, intentional self-harm, initial encounter
T44.5X2D	Poisoning by predominantly beta-adrenoreceptor agonists, intentional self-harm, subsequent encounter
T44.5X2S	Poisoning by predominantly beta-adrenoreceptor agonists, intentional self-harm, sequela
T44.6X2A	Poisoning by alpha-adrenoreceptor antagonists, intentional self-harm, initial encounter
T44.6X2D	Poisoning by alpha-adrenoreceptor antagonists, intentional self-harm, subsequent encounter
T44.6X2S	Poisoning by alpha-adrenoreceptor antagonists, intentional self-harm, sequela
T44.7X2A	Poisoning by beta-adrenoreceptor antagonists, intentional self-harm, initial encounter
T44.7X2D	Poisoning by beta-adrenoreceptor antagonists, intentional self-harm, subsequent encounter
T44.7X2S	Poisoning by beta-adrenoreceptor antagonists, intentional self-harm, sequela
T44.8X2A	Poisoning by centrally-acting and adrenergic-neuron-blocking agents, intentional self-harm, initial encounter
T44.8X2D	Poisoning by centrally-acting and adrenergic-neuron-blocking agents, intentional self-harm, subsequent encounter
T44.8X2S	Poisoning by centrally-acting and adrenergic-neuron-blocking agents, intentional self-harm, sequela
T44.902A	Poisoning by unspecified drugs primarily affecting the autonomic nervous system, intentional self-harm, initial encounter
T44.902D	Poisoning by unspecified drugs primarily affecting the autonomic nervous system, intentional self-harm, subsequent encounter

© 2022 Gujral K et al. *JAMA Network Open.*

T44.902S	Poisoning by unspecified drugs primarily affecting the autonomic nervous system, intentional self-harm, sequela
T44.992A	Poisoning by other drug primarily affecting the autonomic nervous system, intentional self-harm, initial encounter
T44.992D	Poisoning by other drug primarily affecting the autonomic nervous system, intentional self-harm, subsequent encounter
T44.992S	Poisoning by other drug primarily affecting the autonomic nervous system, intentional self-harm, sequela
T45.0X2A	Poisoning by antiallergic and antiemetic drugs, intentional self-harm, initial encounter
T45.0X2D	Poisoning by antiallergic and antiemetic drugs, intentional self-harm, subsequent encounter
T45.0X2S	Poisoning by antiallergic and antiemetic drugs, intentional self-harm, sequela
T45.1X2A	Poisoning by antineoplastic and immunosuppressive drugs, intentional self-harm, initial encounter
T45.1X2D	Poisoning by antineoplastic and immunosuppressive drugs, intentional self-harm, subsequent encounter
T45.1X2S	Poisoning by antineoplastic and immunosuppressive drugs, intentional self-harm, sequela
T45.2X2A	Poisoning by vitamins, intentional self-harm, initial encounter
T45.2X2D	Poisoning by vitamins, intentional self-harm, subsequent encounter
T45.2X2S	Poisoning by vitamins, intentional self-harm, sequela
T45.3X2A	Poisoning by enzymes, intentional self-harm, initial encounter
T45.3X2D	Poisoning by enzymes, intentional self-harm, subsequent encounter
T45.3X2S	Poisoning by enzymes, intentional self-harm, sequela
T45.4X2A	Poisoning by iron and its compounds, intentional self-harm, initial encounter
T45.4X2D	Poisoning by iron and its compounds, intentional self-harm, subsequent encounter
T45.4X2S	Poisoning by iron and its compounds, intentional self-harm, sequela
T45.512A	Poisoning by anticoagulants, intentional self-harm, initial encounter
T45.512D	Poisoning by anticoagulants, intentional self-harm, subsequent encounter
T45.512S	Poisoning by anticoagulants, intentional self-harm, sequela
T45.522A	Poisoning by antithrombotic drugs, intentional self-harm, initial encounter
T45.522D	Poisoning by antithrombotic drugs, intentional self-harm, subsequent encounter
T45.522S	Poisoning by antithrombotic drugs, intentional self-harm, sequela
T45.602A	Poisoning by unspecified fibrinolysis-affecting drugs, intentional self-harm, initial encounter
T45.602D	Poisoning by unspecified fibrinolysis-affecting drugs, intentional self-harm, subsequent encounter
T45.602S	Poisoning by unspecified fibrinolysis-affecting drugs, intentional self-harm, sequela
T45.612A	Poisoning by thrombolytic drug, intentional self-harm, initial encounter
T45.612D	Poisoning by thrombolytic drug, intentional self-harm, subsequent encounter
T45.612S	Poisoning by thrombolytic drug, intentional self-harm, sequela
T45.622A	Poisoning by hemostatic drug, intentional self-harm, initial encounter
T45.622D	Poisoning by hemostatic drug, intentional self-harm, subsequent encounter
T45.622S	Poisoning by hemostatic drug, intentional self-harm, sequela
T45.692A	Poisoning by other fibrinolysis-affecting drugs, intentional self-harm, initial encounter

© 2022 Gujral K et al. *JAMA Network Open.*

T45.692D	Poisoning by other fibrinolysis-affecting drugs, intentional self-harm, subsequent encounter
T45.692S	Poisoning by other fibrinolysis-affecting drugs, intentional self-harm, sequela
T45.7X2A	Poisoning by anticoagulant antagonists, vitamin K and other coagulants, intentional self-harm, initial encounter
T45.7X2D	Poisoning by anticoagulant antagonists, vitamin K and other coagulants, intentional self-harm, subsequent encounter
T45.7X2S	Poisoning by anticoagulant antagonists, vitamin K and other coagulants, intentional self-harm, sequela
T45.8X2A	Poisoning by other primarily systemic and hematological agents, intentional self-harm, initial encounter
T45.8X2D	Poisoning by other primarily systemic and hematological agents, intentional self-harm, subsequent encounter
T45.8X2S	Poisoning by other primarily systemic and hematological agents, intentional self-harm, sequela
T45.92XA	Poisoning by unspecified primarily systemic and hematological agent, intentional self-harm, initial encounter
T45.92XD	Poisoning by unspecified primarily systemic and hematological agent, intentional self-harm, subsequent encounter
T45.92XS	Poisoning by unspecified primarily systemic and hematological agent, intentional self-harm, sequela
T46.0X2A	Poisoning by cardiac-stimulant glycosides and drugs of similar action, intentional self-harm, initial encounter
T46.0X2D	Poisoning by cardiac-stimulant glycosides and drugs of similar action, intentional self-harm, subsequent encounter
T46.0X2S	Poisoning by cardiac-stimulant glycosides and drugs of similar action, intentional self-harm, sequela
T46.1X2A	Poisoning by calcium-channel blockers, intentional self-harm, initial encounter
T46.1X2D	Poisoning by calcium-channel blockers, intentional self-harm, subsequent encounter
T46.1X2S	Poisoning by calcium-channel blockers, intentional self-harm, sequela
T46.2X2A	Poisoning by other antidysrhythmic drugs, intentional self-harm, initial encounter
T46.2X2D	Poisoning by other antidysrhythmic drugs, intentional self-harm, subsequent encounter
T46.2X2S	Poisoning by other antidysrhythmic drugs, intentional self-harm, sequela
T46.3X2A	Poisoning by coronary vasodilators, intentional self-harm, initial encounter
T46.3X2D	Poisoning by coronary vasodilators, intentional self-harm, subsequent encounter
T46.3X2S	Poisoning by coronary vasodilators, intentional self-harm, sequela
T46.4X2A	Poisoning by angiotensin-converting-enzyme inhibitors, intentional self-harm, initial encounter
T46.4X2D	Poisoning by angiotensin-converting-enzyme inhibitors, intentional self-harm, subsequent encounter
T46.4X2S	Poisoning by angiotensin-converting-enzyme inhibitors, intentional self-harm, sequela
T46.5X2A	Poisoning by other antihypertensive drugs, intentional self-harm, initial encounter
T46.5X2D	Poisoning by other antihypertensive drugs, intentional self-harm, subsequent encounter
T46.5X2S	Poisoning by other antihypertensive drugs, intentional self-harm, sequela

© 2022 Gujral K et al. *JAMA Network Open.*

T46.6X2A	Poisoning by antihyperlipidemic and antiarteriosclerotic drugs, intentional self-harm, initial encounter
T46.6X2D	Poisoning by antihyperlipidemic and antiarteriosclerotic drugs, intentional self-harm, subsequent encounter
T46.6X2S	Poisoning by antihyperlipidemic and antiarteriosclerotic drugs, intentional self-harm, sequela
T46.7X2A	Poisoning by peripheral vasodilators, intentional self-harm, initial encounter
T46.7X2D	Poisoning by peripheral vasodilators, intentional self-harm, subsequent encounter
T46.7X2S	Poisoning by peripheral vasodilators, intentional self-harm, sequela
T46.8X2A	Poisoning by antivaricose drugs, including sclerosing agents, intentional self-harm, initial encounter
T46.8X2D	Poisoning by antivaricose drugs, including sclerosing agents, intentional self-harm, subsequent encounter
T46.8X2S	Poisoning by antivaricose drugs, including sclerosing agents, intentional self-harm, sequela
T46.902A	Poisoning by unspecified agents primarily affecting the cardiovascular system, intentional self-harm, initial encounter
T46.902D	Poisoning by unspecified agents primarily affecting the cardiovascular system, intentional self-harm, subsequent encounter
T46.902S	Poisoning by unspecified agents primarily affecting the cardiovascular system, intentional self-harm, sequela
T46.992A	Poisoning by other agents primarily affecting the cardiovascular system, intentional self-harm, initial encounter
T46.992D	Poisoning by other agents primarily affecting the cardiovascular system, intentional self-harm, subsequent encounter
T46.992S	Poisoning by other agents primarily affecting the cardiovascular system, intentional self-harm, sequela
T47.0X2A	Poisoning by histamine H2-receptor blockers, intentional self-harm, initial encounter
T47.0X2D	Poisoning by histamine H2-receptor blockers, intentional self-harm, subsequent encounter
T47.0X2S	Poisoning by histamine H2-receptor blockers, intentional self-harm, sequela
T47.1X2A	Poisoning by other antacids and anti-gastric-secretion drugs, intentional self-harm, initial encounter
T47.1X2D	Poisoning by other antacids and anti-gastric-secretion drugs, intentional self-harm, subsequent encounter
T47.1X2S	Poisoning by other antacids and anti-gastric-secretion drugs, intentional self-harm, sequela
T47.2X2A	Poisoning by stimulant laxatives, intentional self-harm, initial encounter
T47.2X2D	Poisoning by stimulant laxatives, intentional self-harm, subsequent encounter
T47.2X2S	Poisoning by stimulant laxatives, intentional self-harm, sequela
T47.3X2A	Poisoning by saline and osmotic laxatives, intentional self-harm, initial encounter
T47.3X2D	Poisoning by saline and osmotic laxatives, intentional self-harm, subsequent encounter
T47.3X2S	Poisoning by saline and osmotic laxatives, intentional self-harm, sequela
T47.4X2A	Poisoning by other laxatives, intentional self-harm, initial encounter
T47.4X2D	Poisoning by other laxatives, intentional self-harm, subsequent encounter
T47.4X2S	Poisoning by other laxatives, intentional self-harm, sequela
T47.5X2A	Poisoning by digestants, intentional self-harm, initial encounter
T47.5X2D	Poisoning by digestants, intentional self-harm, subsequent encounter

© 2022 Gujral K et al. *JAMA Network Open.*

T47.5X2S	Poisoning by digestants, intentional self-harm, sequela
T47.6X2A	Poisoning by antidiarrheal drugs, intentional self-harm, initial encounter
T47.6X2D	Poisoning by antidiarrheal drugs, intentional self-harm, subsequent encounter
T47.6X2S	Poisoning by antidiarrheal drugs, intentional self-harm, sequela
T47.7X2A	Poisoning by emetics, intentional self-harm, initial encounter
T47.7X2D	Poisoning by emetics, intentional self-harm, subsequent encounter
T47.7X2S	Poisoning by emetics, intentional self-harm, sequela
T47.8X2A	Poisoning by other agents primarily affecting gastrointestinal system, intentional self-harm, initial encounter
T47.8X2D	Poisoning by other agents primarily affecting gastrointestinal system, intentional self-harm, subsequent encounter
T47.8X2S	Poisoning by other agents primarily affecting gastrointestinal system, intentional self-harm, sequela
T47.92XA	Poisoning by unspecified agents primarily affecting the gastrointestinal system, intentional self-harm, initial encounter
T47.92XD	Poisoning by unspecified agents primarily affecting the gastrointestinal system, intentional self-harm, subsequent encounter
T47.92XS	Poisoning by unspecified agents primarily affecting the gastrointestinal system, intentional self-harm, sequela
T48.0X2A	Poisoning by oxytocic drugs, intentional self-harm, initial encounter
T48.0X2D	Poisoning by oxytocic drugs, intentional self-harm, subsequent encounter
T48.0X2S	Poisoning by oxytocic drugs, intentional self-harm, sequela
T48.1X2A	Poisoning by skeletal muscle relaxants [neuromuscular blocking agents], intentional self-harm, initial encounter
T48.1X2D	Poisoning by skeletal muscle relaxants [neuromuscular blocking agents], intentional self-harm, subsequent encounter
T48.1X2S	Poisoning by skeletal muscle relaxants [neuromuscular blocking agents], intentional self-harm, sequela
T48.202A	Poisoning by unspecified drugs acting on muscles, intentional self-harm, initial encounter
T48.202D	Poisoning by unspecified drugs acting on muscles, intentional self-harm, subsequent encounter
T48.202S	Poisoning by unspecified drugs acting on muscles, intentional self-harm, sequela
T48.292A	Poisoning by other drugs acting on muscles, intentional self-harm, initial encounter
T48.292D	Poisoning by other drugs acting on muscles, intentional self-harm, subsequent encounter
T48.292S	Poisoning by other drugs acting on muscles, intentional self-harm, sequela
T48.3X2A	Poisoning by antitussives, intentional self-harm, initial encounter
T48.3X2D	Poisoning by antitussives, intentional self-harm, subsequent encounter
T48.3X2S	Poisoning by antitussives, intentional self-harm, sequela
T48.4X2A	Poisoning by expectorants, intentional self-harm, initial encounter
T48.4X2D	Poisoning by expectorants, intentional self-harm, subsequent encounter
T48.4X2S	Poisoning by expectorants, intentional self-harm, sequela
T48.5X2A	Poisoning by other anti-common-cold drugs, intentional self-harm, initial encounter
T48.5X2D	Poisoning by other anti-common-cold drugs, intentional self-harm, subsequent encounter
T48.5X2S	Poisoning by other anti-common-cold drugs, intentional self-harm, sequela

© 2022 Gujral K et al. *JAMA Network Open.*

T48.6X2A	Poisoning by antiasthmatics, intentional self-harm, initial encounter
T48.6X2D	Poisoning by antiasthmatics, intentional self-harm, subsequent encounter
T48.6X2S	Poisoning by antiasthmatics, intentional self-harm, sequela
T48.902A	Poisoning by unspecified agents primarily acting on the respiratory system, intentional self-harm, initial encounter
T48.902D	Poisoning by unspecified agents primarily acting on the respiratory system, intentional self-harm, subsequent encounter
T48.902S	Poisoning by unspecified agents primarily acting on the respiratory system, intentional self-harm, sequela
T48.992A	Poisoning by other agents primarily acting on the respiratory system, intentional self-harm, initial encounter
T48.992D	Poisoning by other agents primarily acting on the respiratory system, intentional self-harm, subsequent encounter
T48.992S	Poisoning by other agents primarily acting on the respiratory system, intentional self-harm, sequela
T49.0X2A	Poisoning by local antifungal, anti-infective and anti-inflammatory drugs, intentional self-harm, initial encounter
T49.0X2D	Poisoning by local antifungal, anti-infective and anti-inflammatory drugs, intentional self-harm, subsequent encounter
T49.0X2S	Poisoning by local antifungal, anti-infective and anti-inflammatory drugs, intentional self-harm, sequela
T49.1X2A	Poisoning by antipruritics, intentional self-harm, initial encounter
T49.1X2D	Poisoning by antipruritics, intentional self-harm, subsequent encounter
T49.1X2S	Poisoning by antipruritics, intentional self-harm, sequela
T49.2X2A	Poisoning by local astringents and local detergents, intentional self-harm, initial encounter
T49.2X2D	Poisoning by local astringents and local detergents, intentional self-harm, subsequent encounter
T49.2X2S	Poisoning by local astringents and local detergents, intentional self-harm, sequela
T49.3X2A	Poisoning by emollients, demulcents and protectants, intentional self-harm, initial encounter
T49.3X2D	Poisoning by emollients, demulcents and protectants, intentional self-harm, subsequent encounter
T49.3X2S	Poisoning by emollients, demulcents and protectants, intentional self-harm, sequela
T49.4X2A	Poisoning by keratolytics, keratoplastics, and other hair treatment drugs and preparations, intentional self-harm, initial encounter
T49.4X2D	Poisoning by keratolytics, keratoplastics, and other hair treatment drugs and preparations, intentional self-harm, subsequent encounter
T49.4X2S	Poisoning by keratolytics, keratoplastics, and other hair treatment drugs and preparations, intentional self-harm, sequela
T49.5X2A	Poisoning by ophthalmological drugs and preparations, intentional self-harm, initial encounter
T49.5X2D	Poisoning by ophthalmological drugs and preparations, intentional self-harm, subsequent encounter
T49.5X2S	Poisoning by ophthalmological drugs and preparations, intentional self-harm, sequela
T49.6X2A	Poisoning by otorhinolaryngological drugs and preparations, intentional self-harm, initial encounter

© 2022 Gujral K et al. *JAMA Network Open.*

T49.6X2D	Poisoning by otorhinolaryngological drugs and preparations, intentional self-harm, subsequent encounter
T49.6X2S	Poisoning by otorhinolaryngological drugs and preparations, intentional self-harm, sequela
T49.7X2A	Poisoning by dental drugs, topically applied, intentional self-harm, initial encounter
T49.7X2D	Poisoning by dental drugs, topically applied, intentional self-harm, subsequent encounter
T49.7X2S	Poisoning by dental drugs, topically applied, intentional self-harm, sequela
T49.8X2A	Poisoning by other topical agents, intentional self-harm, initial encounter
T49.8X2D	Poisoning by other topical agents, intentional self-harm, subsequent encounter
T49.8X2S	Poisoning by other topical agents, intentional self-harm, sequela
T49.92XA	Poisoning by unspecified topical agent, intentional self-harm, initial encounter
T49.92XD	Poisoning by unspecified topical agent, intentional self-harm, subsequent encounter
T49.92XS	Poisoning by unspecified topical agent, intentional self-harm, sequela
T50.0X2A	Poisoning by mineralocorticoids and their antagonists, intentional self-harm, initial encounter
T50.0X2D	Poisoning by mineralocorticoids and their antagonists, intentional self-harm, subsequent encounter
T50.0X2S	Poisoning by mineralocorticoids and their antagonists, intentional self-harm, sequela
T50.1X2A	Poisoning by loop [high-ceiling] diuretics, intentional self-harm, initial encounter
T50.1X2D	Poisoning by loop [high-ceiling] diuretics, intentional self-harm, subsequent encounter
T50.1X2S	Poisoning by loop [high-ceiling] diuretics, intentional self-harm, sequela
T50.2X2A	Poisoning by carbonic-anhydrase inhibitors, benzothiadiazides and other diuretics, intentional self-harm, initial encounter
T50.2X2D	Poisoning by carbonic-anhydrase inhibitors, benzothiadiazides and other diuretics, intentional self-harm, subsequent encounter
T50.2X2S	Poisoning by carbonic-anhydrase inhibitors, benzothiadiazides and other diuretics, intentional self-harm, sequela
T50.3X2A	Poisoning by electrolytic, caloric and water-balance agents, intentional self-harm, initial encounter
T50.3X2D	Poisoning by electrolytic, caloric and water-balance agents, intentional self-harm, subsequent encounter
T50.3X2S	Poisoning by electrolytic, caloric and water-balance agents, intentional self-harm, sequela
T50.4X2A	Poisoning by drugs affecting uric acid metabolism, intentional self-harm, initial encounter
T50.4X2D	Poisoning by drugs affecting uric acid metabolism, intentional self-harm, subsequent encounter
T50.4X2S	Poisoning by drugs affecting uric acid metabolism, intentional self-harm, sequela
T50.5X2A	Poisoning by appetite depressants, intentional self-harm, initial encounter
T50.5X2D	Poisoning by appetite depressants, intentional self-harm, subsequent encounter
T50.5X2S	Poisoning by appetite depressants, intentional self-harm, sequela
T50.6X2A	Poisoning by antidotes and chelating agents, intentional self-harm, initial encounter
T50.6X2D	Poisoning by antidotes and chelating agents, intentional self-harm, subsequent encounter
T50.6X2S	Poisoning by antidotes and chelating agents, intentional self-harm, sequela
T50.7X2A	Poisoning by analeptics and opioid receptor antagonists, intentional self-harm, initial encounter
T50.7X2D	Poisoning by analeptics and opioid receptor antagonists, intentional self-harm, subsequent encounter

© 2022 Gujral K et al. *JAMA Network Open.*

T50.7X2S	Poisoning by analeptics and opioid receptor antagonists, intentional self-harm, sequela
T50.8X2A	Poisoning by diagnostic agents, intentional self-harm, initial encounter
T50.8X2D	Poisoning by diagnostic agents, intentional self-harm, subsequent encounter
T50.8X2S	Poisoning by diagnostic agents, intentional self-harm, sequela
T50.902A	Poisoning by unspecified drugs, medicaments and biological substances, intentional self-harm, initial encounter
T50.902D	Poisoning by unspecified drugs, medicaments and biological substances, intentional self-harm, subsequent encounter
T50.902S	Poisoning by unspecified drugs, medicaments and biological substances, intentional self-harm, sequela
T50.912A	Poisoning by multiple unspecified drugs, medicaments and biological substances, intentional self-harm, initial encounter
T50.912D	Poisoning by multiple unspecified drugs, medicaments and biological substances, intentional self-harm, subsequent encounter
T50.912S	Poisoning by multiple unspecified drugs, medicaments and biological substances, intentional self-harm, sequela
T50.992A	Poisoning by other drugs, medicaments and biological substances, intentional self-harm, initial encounter
T50.992D	Poisoning by other drugs, medicaments and biological substances, intentional self-harm, subsequent encounter
T50.992S	Poisoning by other drugs, medicaments and biological substances, intentional self-harm, sequela
T50.A12A	Poisoning by pertussis vaccine, including combinations with a pertussis component, intentional self-harm, initial encounter
T50.A12D	Poisoning by pertussis vaccine, including combinations with a pertussis component, intentional self-harm, subsequent encounter
T50.A12S	Poisoning by pertussis vaccine, including combinations with a pertussis component, intentional self-harm, sequela
T50.A22A	Poisoning by mixed bacterial vaccines without a pertussis component, intentional self-harm, initial encounter
T50.A22D	Poisoning by mixed bacterial vaccines without a pertussis component, intentional self-harm, subsequent encounter
T50.A22S	Poisoning by mixed bacterial vaccines without a pertussis component, intentional self-harm, sequela
T50.A92A	Poisoning by other bacterial vaccines, intentional self-harm, initial encounter
T50.A92D	Poisoning by other bacterial vaccines, intentional self-harm, subsequent encounter
T50.A92S	Poisoning by other bacterial vaccines, intentional self-harm, sequela
T50.B12A	Poisoning by smallpox vaccines, intentional self-harm, initial encounter
T50.B12D	Poisoning by smallpox vaccines, intentional self-harm, subsequent encounter
T50.B12S	Poisoning by smallpox vaccines, intentional self-harm, sequela
T50.B92A	Poisoning by other viral vaccines, intentional self-harm, initial encounter
T50.B92D	Poisoning by other viral vaccines, intentional self-harm, subsequent encounter
T50.B92S	Poisoning by other viral vaccines, intentional self-harm, sequela
T50.Z12A	Poisoning by immunoglobulin, intentional self-harm, initial encounter
T50.Z12D	Poisoning by immunoglobulin, intentional self-harm, subsequent encounter

T50.Z12S	Poisoning by immunoglobulin, intentional self-harm, sequela
T50.Z92A	Poisoning by other vaccines and biological substances, intentional self-harm, initial encounter
T50.Z92D	Poisoning by other vaccines and biological substances, intentional self-harm, subsequent encounter
T50.Z92S	Poisoning by other vaccines and biological substances, intentional self-harm, sequela
T51.0X2A	Toxic effect of ethanol, intentional self-harm, initial encounter
T51.0X2D	Toxic effect of ethanol, intentional self-harm, subsequent encounter
T51.0X2S	Toxic effect of ethanol, intentional self-harm, sequela
T51.1X2A	Toxic effect of methanol, intentional self-harm, initial encounter
T51.1X2D	Toxic effect of methanol, intentional self-harm, subsequent encounter
T51.1X2S	Toxic effect of methanol, intentional self-harm, sequela
T51.2X2A	Toxic effect of 2-Propanol, intentional self-harm, initial encounter
T51.2X2D	Toxic effect of 2-Propanol, intentional self-harm, subsequent encounter
T51.2X2S	Toxic effect of 2-Propanol, intentional self-harm, sequela
T51.3X2A	Toxic effect of fusel oil, intentional self-harm, initial encounter
T51.3X2D	Toxic effect of fusel oil, intentional self-harm, subsequent encounter
T51.3X2S	Toxic effect of fusel oil, intentional self-harm, sequela
T51.8X2A	Toxic effect of other alcohols, intentional self-harm, initial encounter
T51.8X2D	Toxic effect of other alcohols, intentional self-harm, subsequent encounter
T51.8X2S	Toxic effect of other alcohols, intentional self-harm, sequela
T51.92XA	Toxic effect of unspecified alcohol, intentional self-harm, initial encounter
T51.92XD	Toxic effect of unspecified alcohol, intentional self-harm, subsequent encounter
T51.92XS	Toxic effect of unspecified alcohol, intentional self-harm, sequela
T52.0X2A	Toxic effect of petroleum products, intentional self-harm, initial encounter
T52.0X2D	Toxic effect of petroleum products, intentional self-harm, subsequent encounter
T52.0X2S	Toxic effect of petroleum products, intentional self-harm, sequela
T52.1X2A	Toxic effect of benzene, intentional self-harm, initial encounter
T52.1X2D	Toxic effect of benzene, intentional self-harm, subsequent encounter
T52.1X2S	Toxic effect of benzene, intentional self-harm, sequela
T52.2X2A	Toxic effect of homologues of benzene, intentional self-harm, initial encounter
T52.2X2D	Toxic effect of homologues of benzene, intentional self-harm, subsequent encounter
T52.2X2S	Toxic effect of homologues of benzene, intentional self-harm, sequela
T52.3X2A	Toxic effect of glycols, intentional self-harm, initial encounter
T52.3X2D	Toxic effect of glycols, intentional self-harm, subsequent encounter
T52.3X2S	Toxic effect of glycols, intentional self-harm, sequela
T52.4X2A	Toxic effect of ketones, intentional self-harm, initial encounter
T52.4X2D	Toxic effect of ketones, intentional self-harm, subsequent encounter
T52.4X2S	Toxic effect of ketones, intentional self-harm, sequela
T52.8X2A	Toxic effect of other organic solvents, intentional self-harm, initial encounter
T52.8X2D	Toxic effect of other organic solvents, intentional self-harm, subsequent encounter
T52.8X2S	Toxic effect of other organic solvents, intentional self-harm, sequela
T52.92XA	Toxic effect of unspecified organic solvent, intentional self-harm, initial encounter

© 2022 Gujral K et al. *JAMA Network Open.*

T52.92XD	Toxic effect of unspecified organic solvent, intentional self-harm, subsequent encounter
T52.92XS	Toxic effect of unspecified organic solvent, intentional self-harm, sequela
T53.0X2A	Toxic effect of carbon tetrachloride, intentional self-harm, initial encounter
T53.0X2D	Toxic effect of carbon tetrachloride, intentional self-harm, subsequent encounter
T53.0X2S	Toxic effect of carbon tetrachloride, intentional self-harm, sequela
T53.1X2A	Toxic effect of chloroform, intentional self-harm, initial encounter
T53.1X2D	Toxic effect of chloroform, intentional self-harm, subsequent encounter
T53.1X2S	Toxic effect of chloroform, intentional self-harm, sequela
T53.2X2A	Toxic effect of trichloroethylene, intentional self-harm, initial encounter
T53.2X2D	Toxic effect of trichloroethylene, intentional self-harm, subsequent encounter
T53.2X2S	Toxic effect of trichloroethylene, intentional self-harm, sequela
T53.3X2A	Toxic effect of tetrachloroethylene, intentional self-harm, initial encounter
T53.3X2D	Toxic effect of tetrachloroethylene, intentional self-harm, subsequent encounter
T53.3X2S	Toxic effect of tetrachloroethylene, intentional self-harm, sequela
T53.4X2A	Toxic effect of dichloromethane, intentional self-harm, initial encounter
T53.4X2D	Toxic effect of dichloromethane, intentional self-harm, subsequent encounter
T53.4X2S	Toxic effect of dichloromethane, intentional self-harm, sequela
T53.5X2A	Toxic effect of chlorofluorocarbons, intentional self-harm, initial encounter
T53.5X2D	Toxic effect of chlorofluorocarbons, intentional self-harm, subsequent encounter
T53.5X2S	Toxic effect of chlorofluorocarbons, intentional self-harm, sequela
T53.6X2A	Toxic effect of other halogen derivatives of aliphatic hydrocarbons, intentional self-harm, initial encounter
T53.6X2D	Toxic effect of other halogen derivatives of aliphatic hydrocarbons, intentional self-harm, subsequent encounter
T53.6X2S	Toxic effect of other halogen derivatives of aliphatic hydrocarbons, intentional self-harm, sequela
T53.7X2A	Toxic effect of other halogen derivatives of aromatic hydrocarbons, intentional self-harm, initial encounter
T53.7X2D	Toxic effect of other halogen derivatives of aromatic hydrocarbons, intentional self-harm, subsequent encounter
T53.7X2S	Toxic effect of other halogen derivatives of aromatic hydrocarbons, intentional self-harm, sequela
T53.92XA	Toxic effect of unspecified halogen derivatives of aliphatic and aromatic hydrocarbons, intentional self-harm, initial encounter
T53.92XD	Toxic effect of unspecified halogen derivatives of aliphatic and aromatic hydrocarbons, intentional self-harm, subsequent encounter
T53.92XS	Toxic effect of unspecified halogen derivatives of aliphatic and aromatic hydrocarbons, intentional self-harm, sequela
T54.0X2A	Toxic effect of phenol and phenol homologues, intentional self-harm, initial encounter
T54.0X2D	Toxic effect of phenol and phenol homologues, intentional self-harm, subsequent encounter
T54.0X2S	Toxic effect of phenol and phenol homologues, intentional self-harm, sequela
T54.1X2A	Toxic effect of other corrosive organic compounds, intentional self-harm, initial encounter
T54.1X2D	Toxic effect of other corrosive organic compounds, intentional self-harm, subsequent encounter

© 2022 Gujral K et al. *JAMA Network Open.*

T54.1X2S	Toxic effect of other corrosive organic compounds, intentional self-harm, sequela
T54.2X2A	Toxic effect of corrosive acids and acid-like substances, intentional self-harm, initial encounter
T54.2X2D	Toxic effect of corrosive acids and acid-like substances, intentional self-harm, subsequent encounter
T54.2X2S	Toxic effect of corrosive acids and acid-like substances, intentional self-harm, sequela
T54.3X2A	Toxic effect of corrosive alkalis and alkali-like substances, intentional self-harm, initial encounter
T54.3X2D	Toxic effect of corrosive alkalis and alkali-like substances, intentional self-harm, subsequent encounter
T54.3X2S	Toxic effect of corrosive alkalis and alkali-like substances, intentional self-harm, sequela
T54.92XA	Toxic effect of unspecified corrosive substance, intentional self-harm, initial encounter
T54.92XD	Toxic effect of unspecified corrosive substance, intentional self-harm, subsequent encounter
T54.92XS	Toxic effect of unspecified corrosive substance, intentional self-harm, sequela
T55.0X2A	Toxic effect of soaps, intentional self-harm, initial encounter
T55.0X2D	Toxic effect of soaps, intentional self-harm, subsequent encounter
T55.0X2S	Toxic effect of soaps, intentional self-harm, sequela
T55.1X2A	Toxic effect of detergents, intentional self-harm, initial encounter
T55.1X2D	Toxic effect of detergents, intentional self-harm, subsequent encounter
T55.1X2S	Toxic effect of detergents, intentional self-harm, sequela
T56.0X2A	Toxic effect of lead and its compounds, intentional self-harm, initial encounter
T56.0X2D	Toxic effect of lead and its compounds, intentional self-harm, subsequent encounter
T56.0X2S	Toxic effect of lead and its compounds, intentional self-harm, sequela
T56.1X2A	Toxic effect of mercury and its compounds, intentional self-harm, initial encounter
T56.1X2D	Toxic effect of mercury and its compounds, intentional self-harm, subsequent encounter
T56.1X2S	Toxic effect of mercury and its compounds, intentional self-harm, sequela
T56.2X2A	Toxic effect of chromium and its compounds, intentional self-harm, initial encounter
T56.2X2D	Toxic effect of chromium and its compounds, intentional self-harm, subsequent encounter
T56.2X2S	Toxic effect of chromium and its compounds, intentional self-harm, sequela
T56.3X2A	Toxic effect of cadmium and its compounds, intentional self-harm, initial encounter
T56.3X2D	Toxic effect of cadmium and its compounds, intentional self-harm, subsequent encounter
T56.3X2S	Toxic effect of cadmium and its compounds, intentional self-harm, sequela
T56.4X2A	Toxic effect of copper and its compounds, intentional self-harm, initial encounter
T56.4X2D	Toxic effect of copper and its compounds, intentional self-harm, subsequent encounter
T56.4X2S	Toxic effect of copper and its compounds, intentional self-harm, sequela
T56.5X2A	Toxic effect of zinc and its compounds, intentional self-harm, initial encounter
T56.5X2D	Toxic effect of zinc and its compounds, intentional self-harm, subsequent encounter
T56.5X2S	Toxic effect of zinc and its compounds, intentional self-harm, sequela
T56.6X2A	Toxic effect of tin and its compounds, intentional self-harm, initial encounter
T56.6X2D	Toxic effect of tin and its compounds, intentional self-harm, subsequent encounter
T56.6X2S	Toxic effect of tin and its compounds, intentional self-harm, sequela
T56.7X2A	Toxic effect of beryllium and its compounds, intentional self-harm, initial encounter
T56.7X2D	Toxic effect of beryllium and its compounds, intentional self-harm, subsequent encounter

© 2022 Gujral K et al. *JAMA Network Open.*

T56.7X2S	Toxic effect of beryllium and its compounds, intentional self-harm, sequela
T56.812A	Toxic effect of thallium, intentional self-harm, initial encounter
T56.812D	Toxic effect of thallium, intentional self-harm, subsequent encounter
T56.812S	Toxic effect of thallium, intentional self-harm, sequela
T56.892A	Toxic effect of other metals, intentional self-harm, initial encounter
T56.892D	Toxic effect of other metals, intentional self-harm, subsequent encounter
T56.892S	Toxic effect of other metals, intentional self-harm, sequela
T56.92XA	Toxic effect of unspecified metal, intentional self-harm, initial encounter
T56.92XD	Toxic effect of unspecified metal, intentional self-harm, subsequent encounter
T56.92XS	Toxic effect of unspecified metal, intentional self-harm, sequela
T57.0X2A	Toxic effect of arsenic and its compounds, intentional self-harm, initial encounter
T57.0X2D	Toxic effect of arsenic and its compounds, intentional self-harm, subsequent encounter
T57.0X2S	Toxic effect of arsenic and its compounds, intentional self-harm, sequela
T57.1X2A	Toxic effect of phosphorus and its compounds, intentional self-harm, initial encounter
T57.1X2D	Toxic effect of phosphorus and its compounds, intentional self-harm, subsequent encounter
T57.1X2S	Toxic effect of phosphorus and its compounds, intentional self-harm, sequela
T57.2X2A	Toxic effect of manganese and its compounds, intentional self-harm, initial encounter
T57.2X2D	Toxic effect of manganese and its compounds, intentional self-harm, subsequent encounter
T57.2X2S	Toxic effect of manganese and its compounds, intentional self-harm, sequela
T57.3X2A	Toxic effect of hydrogen cyanide, intentional self-harm, initial encounter
T57.3X2D	Toxic effect of hydrogen cyanide, intentional self-harm, subsequent encounter
T57.3X2S	Toxic effect of hydrogen cyanide, intentional self-harm, sequela
T57.8X2A	Toxic effect of other specified inorganic substances, intentional self-harm, initial encounter
T57.8X2D	Toxic effect of other specified inorganic substances, intentional self-harm, subsequent encounter
T57.8X2S	Toxic effect of other specified inorganic substances, intentional self-harm, sequela
T57.92XA	Toxic effect of unspecified inorganic substance, intentional self-harm, initial encounter
T57.92XD	Toxic effect of unspecified inorganic substance, intentional self-harm, subsequent encounter
T57.92XS	Toxic effect of unspecified inorganic substance, intentional self-harm, sequela
T58.02XA	Toxic effect of carbon monoxide from motor vehicle exhaust, intentional self-harm, initial encounter
T58.02XD	Toxic effect of carbon monoxide from motor vehicle exhaust, intentional self-harm, subsequent encounter
T58.02XS	Toxic effect of carbon monoxide from motor vehicle exhaust, intentional self-harm, sequela
T58.12XA	Toxic effect of carbon monoxide from utility gas, intentional self-harm, initial encounter
T58.12XD	Toxic effect of carbon monoxide from utility gas, intentional self-harm, subsequent encounter
T58.12XS	Toxic effect of carbon monoxide from utility gas, intentional self-harm, sequela
T58.2X2A	Toxic effect of carbon monoxide from incomplete combustion of other domestic fuels, intentional self-harm, initial encounter

© 2022 Gujral K et al. *JAMA Network Open.*

T58.2X2D	Toxic effect of carbon monoxide from incomplete combustion of other domestic fuels, intentional self-harm, subsequent encounter
T58.2X2S	Toxic effect of carbon monoxide from incomplete combustion of other domestic fuels, intentional self-harm, sequela
T58.8X2A	Toxic effect of carbon monoxide from other source, intentional self-harm, initial encounter
T58.8X2D	Toxic effect of carbon monoxide from other source, intentional self-harm, subsequent encounter
T58.8X2S	Toxic effect of carbon monoxide from other source, intentional self-harm, sequela
T58.92XA	Toxic effect of carbon monoxide from unspecified source, intentional self-harm, initial encounter
T58.92XD	Toxic effect of carbon monoxide from unspecified source, intentional self-harm, subsequent encounter
T58.92XS	Toxic effect of carbon monoxide from unspecified source, intentional self-harm, sequela
T59.0X2A	Toxic effect of nitrogen oxides, intentional self-harm, initial encounter
T59.0X2D	Toxic effect of nitrogen oxides, intentional self-harm, subsequent encounter
T59.0X2S	Toxic effect of nitrogen oxides, intentional self-harm, sequela
T59.1X2A	Toxic effect of sulfur dioxide, intentional self-harm, initial encounter
T59.1X2D	Toxic effect of sulfur dioxide, intentional self-harm, subsequent encounter
T59.1X2S	Toxic effect of sulfur dioxide, intentional self-harm, sequela
T59.2X2A	Toxic effect of formaldehyde, intentional self-harm, initial encounter
T59.2X2D	Toxic effect of formaldehyde, intentional self-harm, subsequent encounter
T59.2X2S	Toxic effect of formaldehyde, intentional self-harm, sequela
T59.3X2A	Toxic effect of lacrimogenic gas, intentional self-harm, initial encounter
T59.3X2D	Toxic effect of lacrimogenic gas, intentional self-harm, subsequent encounter
T59.3X2S	Toxic effect of lacrimogenic gas, intentional self-harm, sequela
T59.4X2A	Toxic effect of chlorine gas, intentional self-harm, initial encounter
T59.4X2D	Toxic effect of chlorine gas, intentional self-harm, subsequent encounter
T59.4X2S	Toxic effect of chlorine gas, intentional self-harm, sequela
T59.5X2A	Toxic effect of fluorine gas and hydrogen fluoride, intentional self-harm, initial encounter
T59.5X2D	Toxic effect of fluorine gas and hydrogen fluoride, intentional self-harm, subsequent encounter
T59.5X2S	Toxic effect of fluorine gas and hydrogen fluoride, intentional self-harm, sequela
T59.6X2A	Toxic effect of hydrogen sulfide, intentional self-harm, initial encounter
T59.6X2D	Toxic effect of hydrogen sulfide, intentional self-harm, subsequent encounter
T59.6X2S	Toxic effect of hydrogen sulfide, intentional self-harm, sequela
T59.7X2A	Toxic effect of carbon dioxide, intentional self-harm, initial encounter
T59.7X2D	Toxic effect of carbon dioxide, intentional self-harm, subsequent encounter
T59.7X2S	Toxic effect of carbon dioxide, intentional self-harm, sequela
T59.812A	Toxic effect of smoke, intentional self-harm, initial encounter
T59.812D	Toxic effect of smoke, intentional self-harm, subsequent encounter
T59.812S	Toxic effect of smoke, intentional self-harm, sequela
T59.892A	Toxic effect of other specified gases, fumes and vapors, intentional self-harm, initial encounter

© 2022 Gujral K et al. *JAMA Network Open.*

T59.892D	Toxic effect of other specified gases, fumes and vapors, intentional self-harm, subsequent encounter
T59.892S	Toxic effect of other specified gases, fumes and vapors, intentional self-harm, sequela
T59.92XA	Toxic effect of unspecified gases, fumes and vapors, intentional self-harm, initial encounter
T59.92XD	Toxic effect of unspecified gases, fumes and vapors, intentional self-harm, subsequent encounter
T59.92XS	Toxic effect of unspecified gases, fumes and vapors, intentional self-harm, sequela
T60.0X2A	Toxic effect of organophosphate and carbamate insecticides, intentional self-harm, initial encounter
T60.0X2D	Toxic effect of organophosphate and carbamate insecticides, intentional self-harm, subsequent encounter
T60.0X2S	Toxic effect of organophosphate and carbamate insecticides, intentional self-harm, sequela
T60.1X2A	Toxic effect of halogenated insecticides, intentional self-harm, initial encounter
T60.1X2D	Toxic effect of halogenated insecticides, intentional self-harm, subsequent encounter
T60.1X2S	Toxic effect of halogenated insecticides, intentional self-harm, sequela
T60.2X2A	Toxic effect of other insecticides, intentional self-harm, initial encounter
T60.2X2D	Toxic effect of other insecticides, intentional self-harm, subsequent encounter
T60.2X2S	Toxic effect of other insecticides, intentional self-harm, sequela
T60.3X2A	Toxic effect of herbicides and fungicides, intentional self-harm, initial encounter
T60.3X2D	Toxic effect of herbicides and fungicides, intentional self-harm, subsequent encounter
T60.3X2S	Toxic effect of herbicides and fungicides, intentional self-harm, sequela
T60.4X2A	Toxic effect of rodenticides, intentional self-harm, initial encounter
T60.4X2D	Toxic effect of rodenticides, intentional self-harm, subsequent encounter
T60.4X2S	Toxic effect of rodenticides, intentional self-harm, sequela
T60.8X2A	Toxic effect of other pesticides, intentional self-harm, initial encounter
T60.8X2D	Toxic effect of other pesticides, intentional self-harm, subsequent encounter
T60.8X2S	Toxic effect of other pesticides, intentional self-harm, sequela
T60.92XA	Toxic effect of unspecified pesticide, intentional self-harm, initial encounter
T60.92XD	Toxic effect of unspecified pesticide, intentional self-harm, subsequent encounter
T60.92XS	Toxic effect of unspecified pesticide, intentional self-harm, sequela
T61.02XA	Ciguatera fish poisoning, intentional self-harm, initial encounter
T61.02XD	Ciguatera fish poisoning, intentional self-harm, subsequent encounter
T61.02XS	Ciguatera fish poisoning, intentional self-harm, sequela
T61.12XA	Scombroid fish poisoning, intentional self-harm, initial encounter
T61.12XD	Scombroid fish poisoning, intentional self-harm, subsequent encounter
T61.12XS	Scombroid fish poisoning, intentional self-harm, sequela
T61.772A	Other fish poisoning, intentional self-harm, initial encounter
T61.772D	Other fish poisoning, intentional self-harm, subsequent encounter
T61.772S	Other fish poisoning, intentional self-harm, sequela
T61.782A	Other shellfish poisoning, intentional self-harm, initial encounter
T61.782D	Other shellfish poisoning, intentional self-harm, subsequent encounter
T61.782S	Other shellfish poisoning, intentional self-harm, sequela

© 2022 Gujral K et al. *JAMA Network Open.*

T61.8X2A	Toxic effect of other seafood, intentional self-harm, initial encounter
T61.8X2D	Toxic effect of other seafood, intentional self-harm, subsequent encounter
T61.8X2S	Toxic effect of other seafood, intentional self-harm, sequela
T61.92XA	Toxic effect of unspecified seafood, intentional self-harm, initial encounter
T61.92XD	Toxic effect of unspecified seafood, intentional self-harm, subsequent encounter
T61.92XS	Toxic effect of unspecified seafood, intentional self-harm, sequela
T62.0X2A	Toxic effect of ingested mushrooms, intentional self-harm, initial encounter
T62.0X2D	Toxic effect of ingested mushrooms, intentional self-harm, subsequent encounter
T62.0X2S	Toxic effect of ingested mushrooms, intentional self-harm, sequela
T62.1X2A	Toxic effect of ingested berries, intentional self-harm, initial encounter
T62.1X2D	Toxic effect of ingested berries, intentional self-harm, subsequent encounter
T62.1X2S	Toxic effect of ingested berries, intentional self-harm, sequela
T62.2X2A	Toxic effect of other ingested (parts of) plant(s), intentional self-harm, initial encounter
T62.2X2D	Toxic effect of other ingested (parts of) plant(s), intentional self-harm, subsequent encounter
T62.2X2S	Toxic effect of other ingested (parts of) plant(s), intentional self-harm, sequela
T62.8X2A	Toxic effect of other specified noxious substances eaten as food, intentional self-harm, initial encounter
T62.8X2D	Toxic effect of other specified noxious substances eaten as food, intentional self-harm, subsequent encounter
T62.8X2S	Toxic effect of other specified noxious substances eaten as food, intentional self-harm, sequela
T62.92XA	Toxic effect of unspecified noxious substance eaten as food, intentional self-harm, initial encounter
T62.92XD	Toxic effect of unspecified noxious substance eaten as food, intentional self-harm, subsequent encounter
T62.92XS	Toxic effect of unspecified noxious substance eaten as food, intentional self-harm, sequela
T63.002A	Toxic effect of unspecified snake venom, intentional self-harm, initial encounter
T63.002D	Toxic effect of unspecified snake venom, intentional self-harm, subsequent encounter
T63.002S	Toxic effect of unspecified snake venom, intentional self-harm, sequela
T63.012A	Toxic effect of rattlesnake venom, intentional self-harm, initial encounter
T63.012D	Toxic effect of rattlesnake venom, intentional self-harm, subsequent encounter
T63.012S	Toxic effect of rattlesnake venom, intentional self-harm, sequela
T63.022A	Toxic effect of coral snake venom, intentional self-harm, initial encounter
T63.022D	Toxic effect of coral snake venom, intentional self-harm, subsequent encounter
T63.022S	Toxic effect of coral snake venom, intentional self-harm, sequela
T63.032A	Toxic effect of taipan venom, intentional self-harm, initial encounter
T63.032D	Toxic effect of taipan venom, intentional self-harm, subsequent encounter
T63.032S	Toxic effect of taipan venom, intentional self-harm, sequela
T63.042A	Toxic effect of cobra venom, intentional self-harm, initial encounter
T63.042D	Toxic effect of cobra venom, intentional self-harm, subsequent encounter
T63.042S	Toxic effect of cobra venom, intentional self-harm, sequela
T63.062A	Toxic effect of venom of other North and South American snake, intentional self-harm, initial encounter

© 2022 Gujral K et al. *JAMA Network Open.*

T63.062D	Toxic effect of venom of other North and South American snake, intentional self-harm, subsequent encounter
T63.062S	Toxic effect of venom of other North and South American snake, intentional self-harm, sequela
T63.072A	Toxic effect of venom of other Australian snake, intentional self-harm, initial encounter
T63.072D	Toxic effect of venom of other Australian snake, intentional self-harm, subsequent encounter
T63.072S	Toxic effect of venom of other Australian snake, intentional self-harm, sequela
T63.082A	Toxic effect of venom of other African and Asian snake, intentional self-harm, initial encounter
T63.082D	Toxic effect of venom of other African and Asian snake, intentional self-harm, subsequent encounter
T63.082S	Toxic effect of venom of other African and Asian snake, intentional self-harm, sequela
T63.092A	Toxic effect of venom of other snake, intentional self-harm, initial encounter
T63.092D	Toxic effect of venom of other snake, intentional self-harm, subsequent encounter
T63.092S	Toxic effect of venom of other snake, intentional self-harm, sequela
T63.112A	Toxic effect of venom of gila monster, intentional self-harm, initial encounter
T63.112D	Toxic effect of venom of gila monster, intentional self-harm, subsequent encounter
T63.112S	Toxic effect of venom of gila monster, intentional self-harm, sequela
T63.122A	Toxic effect of venom of other venomous lizard, intentional self-harm, initial encounter
T63.122D	Toxic effect of venom of other venomous lizard, intentional self-harm, subsequent encounter
T63.122S	Toxic effect of venom of other venomous lizard, intentional self-harm, sequela
T63.192A	Toxic effect of venom of other reptiles, intentional self-harm, initial encounter
T63.192D	Toxic effect of venom of other reptiles, intentional self-harm, subsequent encounter
T63.192S	Toxic effect of venom of other reptiles, intentional self-harm, sequela
T63.2X2A	Toxic effect of venom of scorpion, intentional self-harm, initial encounter
T63.2X2D	Toxic effect of venom of scorpion, intentional self-harm, subsequent encounter
T63.2X2S	Toxic effect of venom of scorpion, intentional self-harm, sequela
T63.302A	Toxic effect of unspecified spider venom, intentional self-harm, initial encounter
T63.302D	Toxic effect of unspecified spider venom, intentional self-harm, subsequent encounter
T63.302S	Toxic effect of unspecified spider venom, intentional self-harm, sequela
T63.312A	Toxic effect of venom of black widow spider, intentional self-harm, initial encounter
T63.312D	Toxic effect of venom of black widow spider, intentional self-harm, subsequent encounter
T63.312S	Toxic effect of venom of black widow spider, intentional self-harm, sequela
T63.322A	Toxic effect of venom of tarantula, intentional self-harm, initial encounter
T63.322D	Toxic effect of venom of tarantula, intentional self-harm, subsequent encounter
T63.322S	Toxic effect of venom of tarantula, intentional self-harm, sequela
T63.332A	Toxic effect of venom of brown recluse spider, intentional self-harm, initial encounter
T63.332D	Toxic effect of venom of brown recluse spider, intentional self-harm, subsequent encounter
T63.332S	Toxic effect of venom of brown recluse spider, intentional self-harm, sequela
T63.392A	Toxic effect of venom of other spider, intentional self-harm, initial encounter
T63.392D	Toxic effect of venom of other spider, intentional self-harm, subsequent encounter

© 2022 Gujral K et al. *JAMA Network Open.*

T63.392S	Toxic effect of venom of other spider, intentional self-harm, sequela
T63.412A	Toxic effect of venom of centipedes and venomous millipedes, intentional self-harm, initial encounter
T63.412D	Toxic effect of venom of centipedes and venomous millipedes, intentional self-harm, subsequent encounter
T63.412S	Toxic effect of venom of centipedes and venomous millipedes, intentional self-harm, sequela
T63.422A	Toxic effect of venom of ants, intentional self-harm, initial encounter
T63.422D	Toxic effect of venom of ants, intentional self-harm, subsequent encounter
T63.422S	Toxic effect of venom of ants, intentional self-harm, sequela
T63.432A	Toxic effect of venom of caterpillars, intentional self-harm, initial encounter
T63.432D	Toxic effect of venom of caterpillars, intentional self-harm, subsequent encounter
T63.432S	Toxic effect of venom of caterpillars, intentional self-harm, sequela
T63.442A	Toxic effect of venom of bees, intentional self-harm, initial encounter
T63.442D	Toxic effect of venom of bees, intentional self-harm, subsequent encounter
T63.442S	Toxic effect of venom of bees, intentional self-harm, sequela
T63.452A	Toxic effect of venom of hornets, intentional self-harm, initial encounter
T63.452D	Toxic effect of venom of hornets, intentional self-harm, subsequent encounter
T63.452S	Toxic effect of venom of hornets, intentional self-harm, sequela
T63.462A	Toxic effect of venom of wasps, intentional self-harm, initial encounter
T63.462D	Toxic effect of venom of wasps, intentional self-harm, subsequent encounter
T63.462S	Toxic effect of venom of wasps, intentional self-harm, sequela
T63.482A	Toxic effect of venom of other arthropod, intentional self-harm, initial encounter
T63.482D	Toxic effect of venom of other arthropod, intentional self-harm, subsequent encounter
T63.482S	Toxic effect of venom of other arthropod, intentional self-harm, sequela
T63.512A	Toxic effect of contact with stingray, intentional self-harm, initial encounter
T63.512D	Toxic effect of contact with stingray, intentional self-harm, subsequent encounter
T63.512S	Toxic effect of contact with stingray, intentional self-harm, sequela
T63.592A	Toxic effect of contact with other venomous fish, intentional self-harm, initial encounter
T63.592D	Toxic effect of contact with other venomous fish, intentional self-harm, subsequent encounter
T63.592S	Toxic effect of contact with other venomous fish, intentional self-harm, sequela
T63.612A	Toxic effect of contact with Portugese Man-o-war, intentional self-harm, initial encounter
T63.612D	Toxic effect of contact with Portugese Man-o-war, intentional self-harm, subsequent encounter
T63.612S	Toxic effect of contact with Portugese Man-o-war, intentional self-harm, sequela
T63.622A	Toxic effect of contact with other jellyfish, intentional self-harm, initial encounter
T63.622D	Toxic effect of contact with other jellyfish, intentional self-harm, subsequent encounter
T63.622S	Toxic effect of contact with other jellyfish, intentional self-harm, sequela
T63.632A	Toxic effect of contact with sea anemone, intentional self-harm, initial encounter
T63.632D	Toxic effect of contact with sea anemone, intentional self-harm, subsequent encounter
T63.632S	Toxic effect of contact with sea anemone, intentional self-harm, sequela
T63.692A	Toxic effect of contact with other venomous marine animals, intentional self-harm, initial encounter

© 2022 Gujral K et al. *JAMA Network Open.*

T63.692D	Toxic effect of contact with other venomous marine animals, intentional self-harm, subsequent encounter
T63.692S	Toxic effect of contact with other venomous marine animals, intentional self-harm, sequela
T63.712A	Toxic effect of contact with venomous marine plant, intentional self-harm, initial encounter
T63.712D	Toxic effect of contact with venomous marine plant, intentional self-harm, subsequent encounter
T63.712S	Toxic effect of contact with venomous marine plant, intentional self-harm, sequela
T63.792A	Toxic effect of contact with other venomous plant, intentional self-harm, initial encounter
T63.792D	Toxic effect of contact with other venomous plant, intentional self-harm, subsequent encounter
T63.792S	Toxic effect of contact with other venomous plant, intentional self-harm, sequela
T63.812A	Toxic effect of contact with venomous frog, intentional self-harm, initial encounter
T63.812D	Toxic effect of contact with venomous frog, intentional self-harm, subsequent encounter
T63.812S	Toxic effect of contact with venomous frog, intentional self-harm, sequela
T63.822A	Toxic effect of contact with venomous toad, intentional self-harm, initial encounter
T63.822D	Toxic effect of contact with venomous toad, intentional self-harm, subsequent encounter
T63.822S	Toxic effect of contact with venomous toad, intentional self-harm, sequela
T63.832A	Toxic effect of contact with other venomous amphibian, intentional self-harm, initial encounter
T63.832D	Toxic effect of contact with other venomous amphibian, intentional self-harm, subsequent encounter
T63.832S	Toxic effect of contact with other venomous amphibian, intentional self-harm, sequela
T63.892A	Toxic effect of contact with other venomous animals, intentional self-harm, initial encounter
T63.892D	Toxic effect of contact with other venomous animals, intentional self-harm, subsequent encounter
T63.892S	Toxic effect of contact with other venomous animals, intentional self-harm, sequela
T63.92XA	Toxic effect of contact with unspecified venomous animal, intentional self-harm, initial encounter
T63.92XD	Toxic effect of contact with unspecified venomous animal, intentional self-harm, subsequent encounter
T63.92XS	Toxic effect of contact with unspecified venomous animal, intentional self-harm, sequela
T64.02XA	Toxic effect of aflatoxin, intentional self-harm, initial encounter
T64.02XD	Toxic effect of aflatoxin, intentional self-harm, subsequent encounter
T64.02XS	Toxic effect of aflatoxin, intentional self-harm, sequela
T64.82XA	Toxic effect of other mycotoxin food contaminants, intentional self-harm, initial encounter
T64.82XD	Toxic effect of other mycotoxin food contaminants, intentional self-harm, subsequent encounter
T64.82XS	Toxic effect of other mycotoxin food contaminants, intentional self-harm, sequela
T65.0X2A	Toxic effect of cyanides, intentional self-harm, initial encounter
T65.0X2D	Toxic effect of cyanides, intentional self-harm, subsequent encounter
T65.0X2S	Toxic effect of cyanides, intentional self-harm, sequela
T65.1X2A	Toxic effect of strychnine and its salts, intentional self-harm, initial encounter

© 2022 Gujral K et al. *JAMA Network Open.*

T65.1X2D	Toxic effect of strychnine and its salts, intentional self-harm, subsequent encounter
T65.1X2S	Toxic effect of strychnine and its salts, intentional self-harm, sequela
T65.212A	Toxic effect of chewing tobacco, intentional self-harm, initial encounter
T65.212D	Toxic effect of chewing tobacco, intentional self-harm, subsequent encounter
T65.212S	Toxic effect of chewing tobacco, intentional self-harm, sequela
T65.222A	Toxic effect of tobacco cigarettes, intentional self-harm, initial encounter
T65.222D	Toxic effect of tobacco cigarettes, intentional self-harm, subsequent encounter
T65.222S	Toxic effect of tobacco cigarettes, intentional self-harm, sequela
T65.292A	Toxic effect of other tobacco and nicotine, intentional self-harm, initial encounter
T65.292D	Toxic effect of other tobacco and nicotine, intentional self-harm, subsequent encounter
T65.292S	Toxic effect of other tobacco and nicotine, intentional self-harm, sequela
T65.3X2A	Toxic effect of nitroderivatives and aminoderivatives of benzene and its homologues, intentional self-harm, initial encounter
T65.3X2D	Toxic effect of nitroderivatives and aminoderivatives of benzene and its homologues, intentional self-harm, subsequent encounter
T65.3X2S	Toxic effect of nitroderivatives and aminoderivatives of benzene and its homologues, intentional self-harm, sequela
T65.4X2A	Toxic effect of carbon disulfide, intentional self-harm, initial encounter
T65.4X2D	Toxic effect of carbon disulfide, intentional self-harm, subsequent encounter
T65.4X2S	Toxic effect of carbon disulfide, intentional self-harm, sequela
T65.5X2A	Toxic effect of nitroglycerin and other nitric acids and esters, intentional self-harm, initial encounter
T65.5X2D	Toxic effect of nitroglycerin and other nitric acids and esters, intentional self-harm, subsequent encounter
T65.5X2S	Toxic effect of nitroglycerin and other nitric acids and esters, intentional self-harm, sequela
T65.6X2A	Toxic effect of paints and dyes, not elsewhere classified, intentional self-harm, initial encounter
T65.6X2D	Toxic effect of paints and dyes, not elsewhere classified, intentional self-harm, subsequent encounter
T65.6X2S	Toxic effect of paints and dyes, not elsewhere classified, intentional self-harm, sequela
T65.812A	Toxic effect of latex, intentional self-harm, initial encounter
T65.812D	Toxic effect of latex, intentional self-harm, subsequent encounter
T65.812S	Toxic effect of latex, intentional self-harm, sequela
T65.822A	Toxic effect of harmful algae and algae toxins, intentional self-harm, initial encounter
T65.822D	Toxic effect of harmful algae and algae toxins, intentional self-harm, subsequent encounter
T65.822S	Toxic effect of harmful algae and algae toxins, intentional self-harm, sequela
T65.832A	Toxic effect of fiberglass, intentional self-harm, initial encounter
T65.832D	Toxic effect of fiberglass, intentional self-harm, subsequent encounter
T65.832S	Toxic effect of fiberglass, intentional self-harm, sequela
T65.892A	Toxic effect of other specified substances, intentional self-harm, initial encounter
T65.892D	Toxic effect of other specified substances, intentional self-harm, subsequent encounter
T65.892S	Toxic effect of other specified substances, intentional self-harm, sequela

© 2022 Gujral K et al. *JAMA Network Open.*

T65.92XA	Toxic effect of unspecified substance, intentional self-harm, initial encounter
T65.92XD	Toxic effect of unspecified substance, intentional self-harm, subsequent encounter
T65.92XS	Toxic effect of unspecified substance, intentional self-harm, sequela
T71.112A	Asphyxiation due to smothering under pillow, intentional self-harm, initial encounter
T71.112D	Asphyxiation due to smothering under pillow, intentional self-harm, subsequent encounter
T71.112S	Asphyxiation due to smothering under pillow, intentional self-harm, sequela
T71.122A	Asphyxiation due to plastic bag, intentional self-harm, initial encounter
T71.122D	Asphyxiation due to plastic bag, intentional self-harm, subsequent encounter
T71.122S	Asphyxiation due to plastic bag, intentional self-harm, sequela
T71.132A	Asphyxiation due to being trapped in bed linens, intentional self-harm, initial encounter
T71.132D	Asphyxiation due to being trapped in bed linens, intentional self-harm, subsequent encounter
T71.132S	Asphyxiation due to being trapped in bed linens, intentional self-harm, sequela
T71.152A	Asphyxiation due to smothering in furniture, intentional self-harm, initial encounter
T71.152D	Asphyxiation due to smothering in furniture, intentional self-harm, subsequent encounter
T71.152S	Asphyxiation due to smothering in furniture, intentional self-harm, sequela
T71.162A	Asphyxiation due to hanging, intentional self-harm, initial encounter
T71.162D	Asphyxiation due to hanging, intentional self-harm, subsequent encounter
T71.162S	Asphyxiation due to hanging, intentional self-harm, sequela
T71.192A	Asphyxiation due to mechanical threat to breathing due to other causes, intentional self-harm, initial encounter
T71.192D	Asphyxiation due to mechanical threat to breathing due to other causes, intentional self-harm, subsequent encounter
T71.192S	Asphyxiation due to mechanical threat to breathing due to other causes, intentional self-harm, sequela
T71.222A	Asphyxiation due to being trapped in a car trunk, intentional self-harm, initial encounter
T71.222D	Asphyxiation due to being trapped in a car trunk, intentional self-harm, subsequent encounter
T71.222S	Asphyxiation due to being trapped in a car trunk, intentional self-harm, sequela
T71.232A	Asphyxiation due to being trapped in a (discarded) refrigerator, intentional self-harm, initial encounter
T71.232D	Asphyxiation due to being trapped in a (discarded) refrigerator, intentional self-harm, subsequent encounter
T71.232S	Asphyxiation due to being trapped in a (discarded) refrigerator, intentional self-harm, sequela
X71.0XXA	Intentional self-harm by drowning and submersion while in bathtub, initial encounter
X71.0XXD	Intentional self-harm by drowning and submersion while in bathtub, subsequent encounter
X71.0XXS	Intentional self-harm by drowning and submersion while in bathtub, sequela
X71.1XXA	Intentional self-harm by drowning and submersion while in swimming pool, initial encounter
X71.1XXD	Intentional self-harm by drowning and submersion while in swimming pool, subsequent encounter
X71.1XXS	Intentional self-harm by drowning and submersion while in swimming pool, sequela

© 2022 Gujral K et al. *JAMA Network Open.*

X71.2XXA	Intentional self-harm by drowning and submersion after jump into swimming pool, initial encounter
X71.2XXD	Intentional self-harm by drowning and submersion after jump into swimming pool, subsequent encounter
X71.2XXS	Intentional self-harm by drowning and submersion after jump into swimming pool, sequela
X71.3XXA	Intentional self-harm by drowning and submersion in natural water, initial encounter
X71.3XXD	Intentional self-harm by drowning and submersion in natural water, subsequent encounter
X71.3XXS	Intentional self-harm by drowning and submersion in natural water, sequela
X71.8XXA	Other intentional self-harm by drowning and submersion, initial encounter
X71.8XXD	Other intentional self-harm by drowning and submersion, subsequent encounter
X71.8XXS	Other intentional self-harm by drowning and submersion, sequela
X71.9XXA	Intentional self-harm by drowning and submersion, unspecified, initial encounter
X71.9XXD	Intentional self-harm by drowning and submersion, unspecified, subsequent encounter
X71.9XXS	Intentional self-harm by drowning and submersion, unspecified, sequela
X72.XXXA	Intentional self-harm by handgun discharge, initial encounter
X72.XXXD	Intentional self-harm by handgun discharge, subsequent encounter
X72.XXXS	Intentional self-harm by handgun discharge, sequela
X73.0XXA	Intentional self-harm by shotgun discharge, initial encounter
X73.0XXD	Intentional self-harm by shotgun discharge, subsequent encounter
X73.0XXS	Intentional self-harm by shotgun discharge, sequela
X73.1XXA	Intentional self-harm by hunting rifle discharge, initial encounter
X73.1XXD	Intentional self-harm by hunting rifle discharge, subsequent encounter
X73.1XXS	Intentional self-harm by hunting rifle discharge, sequela
X73.2XXA	Intentional self-harm by machine gun discharge, initial encounter
X73.2XXD	Intentional self-harm by machine gun discharge, subsequent encounter
X73.2XXS	Intentional self-harm by machine gun discharge, sequela
X73.8XXA	Intentional self-harm by other larger firearm discharge, initial encounter
X73.8XXD	Intentional self-harm by other larger firearm discharge, subsequent encounter
X73.8XXS	Intentional self-harm by other larger firearm discharge, sequela
X73.9XXA	Intentional self-harm by unspecified larger firearm discharge, initial encounter
X73.9XXD	Intentional self-harm by unspecified larger firearm discharge, subsequent encounter
X73.9XXS	Intentional self-harm by unspecified larger firearm discharge, sequela
X74.01XA	Intentional self-harm by airgun, initial encounter
X74.01XD	Intentional self-harm by airgun, subsequent encounter
X74.01XS	Intentional self-harm by airgun, sequela
X74.02XA	Intentional self-harm by paintball gun, initial encounter
X74.02XD	Intentional self-harm by paintball gun, subsequent encounter
X74.02XS	Intentional self-harm by paintball gun, sequela
X74.09XA	Intentional self-harm by other gas, air or spring-operated gun, initial encounter
X74.09XD	Intentional self-harm by other gas, air or spring-operated gun, subsequent encounter
X74.09XS	Intentional self-harm by other gas, air or spring-operated gun, sequela

© 2022 Gujral K et al. *JAMA Network Open.*

X74.8XXA	Intentional self-harm by other firearm discharge, initial encounter
X74.8XXD	Intentional self-harm by other firearm discharge, subsequent encounter
X74.8XXS	Intentional self-harm by other firearm discharge, sequela
X74.9XXA	Intentional self-harm by unspecified firearm discharge, initial encounter
X74.9XXD	Intentional self-harm by unspecified firearm discharge, subsequent encounter
X74.9XXS	Intentional self-harm by unspecified firearm discharge, sequela
X75.XXXA	Intentional self-harm by explosive material, initial encounter
X75.XXXD	Intentional self-harm by explosive material, subsequent encounter
X75.XXXS	Intentional self-harm by explosive material, sequela
X76.XXXA	Intentional self-harm by smoke, fire and flames, initial encounter
X76.XXXD	Intentional self-harm by smoke, fire and flames, subsequent encounter
X76.XXXS	Intentional self-harm by smoke, fire and flames, sequela
X77.0XXA	Intentional self-harm by steam or hot vapors, initial encounter
X77.0XXD	Intentional self-harm by steam or hot vapors, subsequent encounter
X77.0XXS	Intentional self-harm by steam or hot vapors, sequela
X77.1XXA	Intentional self-harm by hot tap water, initial encounter
X77.1XXD	Intentional self-harm by hot tap water, subsequent encounter
X77.1XXS	Intentional self-harm by hot tap water, sequela
X77.2XXA	Intentional self-harm by other hot fluids, initial encounter
X77.2XXD	Intentional self-harm by other hot fluids, subsequent encounter
X77.2XXS	Intentional self-harm by other hot fluids, sequela
X77.3XXA	Intentional self-harm by hot household appliances, initial encounter
X77.3XXD	Intentional self-harm by hot household appliances, subsequent encounter
X77.3XXS	Intentional self-harm by hot household appliances, sequela
X77.8XXA	Intentional self-harm by other hot objects, initial encounter
X77.8XXD	Intentional self-harm by other hot objects, subsequent encounter
X77.8XXS	Intentional self-harm by other hot objects, sequela
X77.9XXA	Intentional self-harm by unspecified hot objects, initial encounter
X77.9XXD	Intentional self-harm by unspecified hot objects, subsequent encounter
X77.9XXS	Intentional self-harm by unspecified hot objects, sequela
X78.0XXA	Intentional self-harm by sharp glass, initial encounter
X78.0XXD	Intentional self-harm by sharp glass, subsequent encounter
X78.0XXS	Intentional self-harm by sharp glass, sequela
X78.1XXA	Intentional self-harm by knife, initial encounter
X78.1XXD	Intentional self-harm by knife, subsequent encounter
X78.1XXS	Intentional self-harm by knife, sequela
X78.2XXA	Intentional self-harm by sword or dagger, initial encounter
X78.2XXD	Intentional self-harm by sword or dagger, subsequent encounter
X78.2XXS	Intentional self-harm by sword or dagger, sequela
X78.8XXA	Intentional self-harm by other sharp object, initial encounter
X78.8XXD	Intentional self-harm by other sharp object, subsequent encounter
X78.8XXS	Intentional self-harm by other sharp object, sequela
X78.9XXA	Intentional self-harm by unspecified sharp object, initial encounter

© 2022 Gujral K et al. *JAMA Network Open.*

X78.9XXD	Intentional self-harm by unspecified sharp object, subsequent encounter
X78.9XXS	Intentional self-harm by unspecified sharp object, sequela
X79.XXXA	Intentional self-harm by blunt object, initial encounter
X79.XXXD	Intentional self-harm by blunt object, subsequent encounter
X79.XXXS	Intentional self-harm by blunt object, sequela
X80.XXXA	Intentional self-harm by jumping from a high place, initial encounter
X80.XXXD	Intentional self-harm by jumping from a high place, subsequent encounter
X80.XXXS	Intentional self-harm by jumping from a high place, sequela
X81.0XXA	Intentional self-harm by jumping or lying in front of motor vehicle, initial encounter
X81.0XXD	Intentional self-harm by jumping or lying in front of motor vehicle, subsequent encounter
X81.0XXS	Intentional self-harm by jumping or lying in front of motor vehicle, sequela
X81.1XXA	Intentional self-harm by jumping or lying in front of (subway) train, initial encounter
X81.1XXD	Intentional self-harm by jumping or lying in front of (subway) train, subsequent encounter
X81.1XXS	Intentional self-harm by jumping or lying in front of (subway) train, sequela
X81.8XXA	Intentional self-harm by jumping or lying in front of other moving object, initial encounter
X81.8XXD	Intentional self-harm by jumping or lying in front of other moving object, subsequent encounter
X81.8XXS	Intentional self-harm by jumping or lying in front of other moving object, sequela
X82.8XXA	Other intentional self-harm by crashing of motor vehicle, initial encounter
X82.8XXD	Other intentional self-harm by crashing of motor vehicle, subsequent encounter
X82.8XXS	Other intentional self-harm by crashing of motor vehicle, sequela
X83.0XXA	Intentional self-harm by crashing of aircraft, initial encounter
X83.0XXD	Intentional self-harm by crashing of aircraft, subsequent encounter
X83.0XXS	Intentional self-harm by crashing of aircraft, sequela
X83.1XXA	Intentional self-harm by electrocution, initial encounter
X83.1XXD	Intentional self-harm by electrocution, subsequent encounter
X83.1XXS	Intentional self-harm by electrocution, sequela
X83.2XXA	Intentional self-harm by exposure to extremes of cold, initial encounter
X83.2XXD	Intentional self-harm by exposure to extremes of cold, subsequent encounter
X83.2XXS	Intentional self-harm by exposure to extremes of cold, sequela
X83.8XXA	Intentional self-harm by other specified means, initial encounter
X83.8XXD	Intentional self-harm by other specified means, subsequent encounter
X83.8XXS	Intentional self-harm by other specified means, sequela
T14.91XA	Suicide Attempt, initial encounter
T14.91XD	Suicide Attempt, subsequent encounter
T14.91XS	Suicide Attempt, sequela
R45.851	Suicidal ideations

eTable 9: Physical and mental health chronic condition included as covariates

**Physical Health Chronic Conditions**
HIV/AIDS
Allergic and Other Chronic Sinusitis/Rhinitis

© 2022 Gujral K et al. *JAMA Network Open.*

Anemia
Arrhythmia/Conduction Disorder
Asthma
Coronary Artery Disease
Cancer
Cerebrovascular Disease
Coagulation and Hemorrhagic Disorders
Chronic Obstructive Pulmonary Disease
Chronic Pain Syndromes
Dementia
Diabetes Mellitus
Epilepsy/Convulsions
Esophageal/Gastric/Duodenal Disorders
Heart Failure
Liver Disease or Hepatitis C
Hypertension
Inflammatory Bowel Disease
Joint Disorders
Lipid Disorders
Multiple Sclerosis
Overweight/Obesity
Osteoporosis
Parkinson's Disease
Peripheral Nerve Disorders
Renal Failure or Nephropathy
Rheumatologic/Autoimmune Disorders
Spinal Cord Injury or Paralysis
Spine Disorders
Tobacco Use Disorder
Tuberculosis
Traumatic Brain Injury
Thrombocytopenia
Thyroid disorders
Valvular Disease
Vascular Disease
**Mental Health Chronic Conditions**
Alcohol Use Disorders
Anxiety Disorders, Other
Bipolar Disorders
Depression
Drug Use Disorders, nonopioid
Drug Use Disorders, opioid
Personality Disorders

© 2022 Gujral K et al. *JAMA Network Open.*

Psychotic Disorders, Other
PTSD
Schizophrenia

© 2022 Gujral K et al. *JAMA Network Open.*

eAppendix 1. Methods Details

Event study specifications were as follows:

$$\begin{aligned} Y_{i,m} = {} & \beta_0 + \beta_1 TabletRecipient_i + \beta_{2-21}(month\ relative\ to\ tablet\ shipment)_{i,m} + \beta_{22} gender_i \\ & + \beta_{23} race_i + \beta_{24} ethnicity_i + \beta_{25} age_i \\ & + \beta_{26} NumMHconds_i + \beta_{27} NumPHYSconds_i + \beta_{28} Depression_i + \beta_{29} PTSD_i \\ & + \beta_{30} SUD_i + \beta_{31} CANscore_i + \beta_{32} VApriority_i + \beta_{33} maritalstatus_i \\ & + \beta_{34} reachvetstatus_i + \beta_{35} reachvetstatus_i \\ & + \beta_{36} cumltv\ covid\ cases\ in\ county_{i,m} + closestSCfacilityFEs_i \\ & + monthyearFEs_m + \varepsilon \end{aligned}$$

where $Y_{i,m}$ is the outcome of interest, *i* denotes an individual, and m denotes months. β_{2-21} were the coefficients of interest which reflect the tablet associations in each month pre- and post- tablet shipment. These coefficients were plotted on the event study graphs to illustrate the tablet associations. Month relative to tablet shipment was calculated as the tablet shipment month minus the observation month, such that relative month = 0 is the month in which a tablet was shipped to a veteran, and relative month =-1 was the month just prior to tablet shipment, and so on. Note that we excluded relative months = -1 and = 0 from the analysis because treatment assignment i.e. tablet assignment likely occurred in these months and we did not want to attribute tablet assignment-related visits to tablet-associated effects. Excluding month 0 also allowed for visits related to tablet set-up to be omitted. We thus used relative month =-2, i.e. 2 months prior to tablet shipment, as the baseline month, which marks the end of the pre-tablet period. The post-tablet period begins with relative month = 1, i.e. one month after tablet was shipped to veterans.

To obtain the usual DiD estimate, we used the following specification:

$$\begin{aligned} Y_{i,m} = {} & \beta_0 + \beta_1 TabletRecipient_i + \beta_2 (TabletRecipient * PostTabletShipment)_{i,m} + \beta_3 gender_i \\ & + \beta_4 race_i + \beta_5 ethnicity_i + \beta_6 age_i + \beta_7 NumMHconds_i + \beta_8 NumPHYSconds_i \\ & + \beta_9 Depression_i + \beta_{10} PTSD_i + \beta_{11} SUD_i + \beta_{12} CANscore_i + \beta_{13} VApriority_i \\ & + \beta_{14} maritalstatus_i + \beta_{15} reachvetstatus_i + \beta_{16} reachvetstatus_i \\ & + \beta_{17} cumltv\ covid\ cases\ in\ county_{i,m} + closestSCfacilityFEs_i \\ & + monthyearFEs_m + \varepsilon \end{aligned}$$

where all of the variables were the same as in the event study specification, except that the "month relative to tablet shipment" variable was replaced with the variable "TabletRecipient * PostTabletShipment," an interaction of indicators for tablet recipient (vs. non-recipient) and for post- (vs. pre-) tablet shipment observation. The coefficient on this interaction term, β_2 , was the coefficient of interest and was the DiD estimate.

© 2022 Gujral K et al. *JAMA Network Open.*

eFigure 2. Tablet Recipients' Visits for Medication Management and CSRES Compared to The Baseline and Compared to Non-Recipients - Regression Coefficients From Event Study Specifications

Below are event study graphs showing tablet-associations for medication management visits and visits for CSREs across all modalities. In an event study analysis, without a significant visual break in the difference in the pattern of visits pre- and post-tablets, we cannot claim any significant associations between tablets and mental health service use. In eFigure 2, the graph to the left shows that there was a slight upward trend in the difference in medication management visits for tablet recipients compared to non-recipients prior to tablet shipment, a trend that may have continued post-tablets. The graph on the right (eFigure 2) shows that the difference in visits for CSREs for tablet recipients compared to non-recipients was increasing slightly prior to tablet shipment, and post-tablets, the pattern in the difference in visits for tablet recipients does not differ abruptly from the pattern in the pre-tablet period. As such, we cannot claim any significant tablet-associations for these outcomes.

eFigure 2: Tablet recipients' visits for medication management and CSREs compared to the baseline and compared to non-recipients - regression coefficients from event study specifications

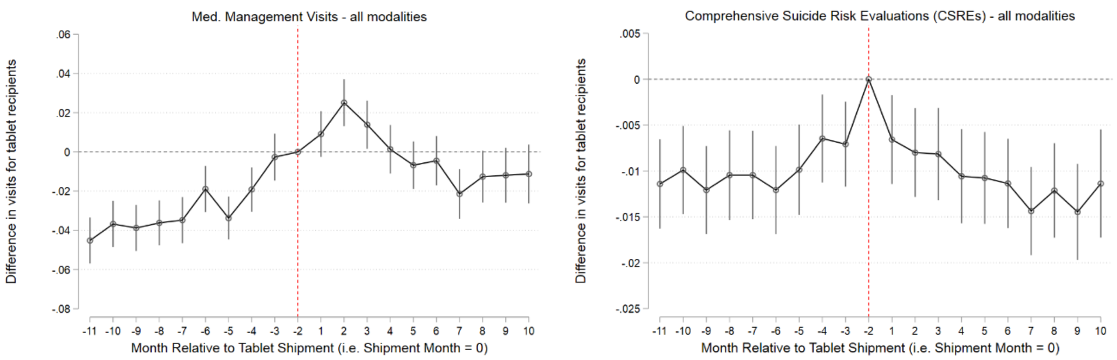

© 2022 Gujral K et al. *JAMA Network Open.*

eFigure 3. Unadjusted average number of monthly visit outcomes for rural tablet recipients and rural tablet non-recipients by calendar month-year

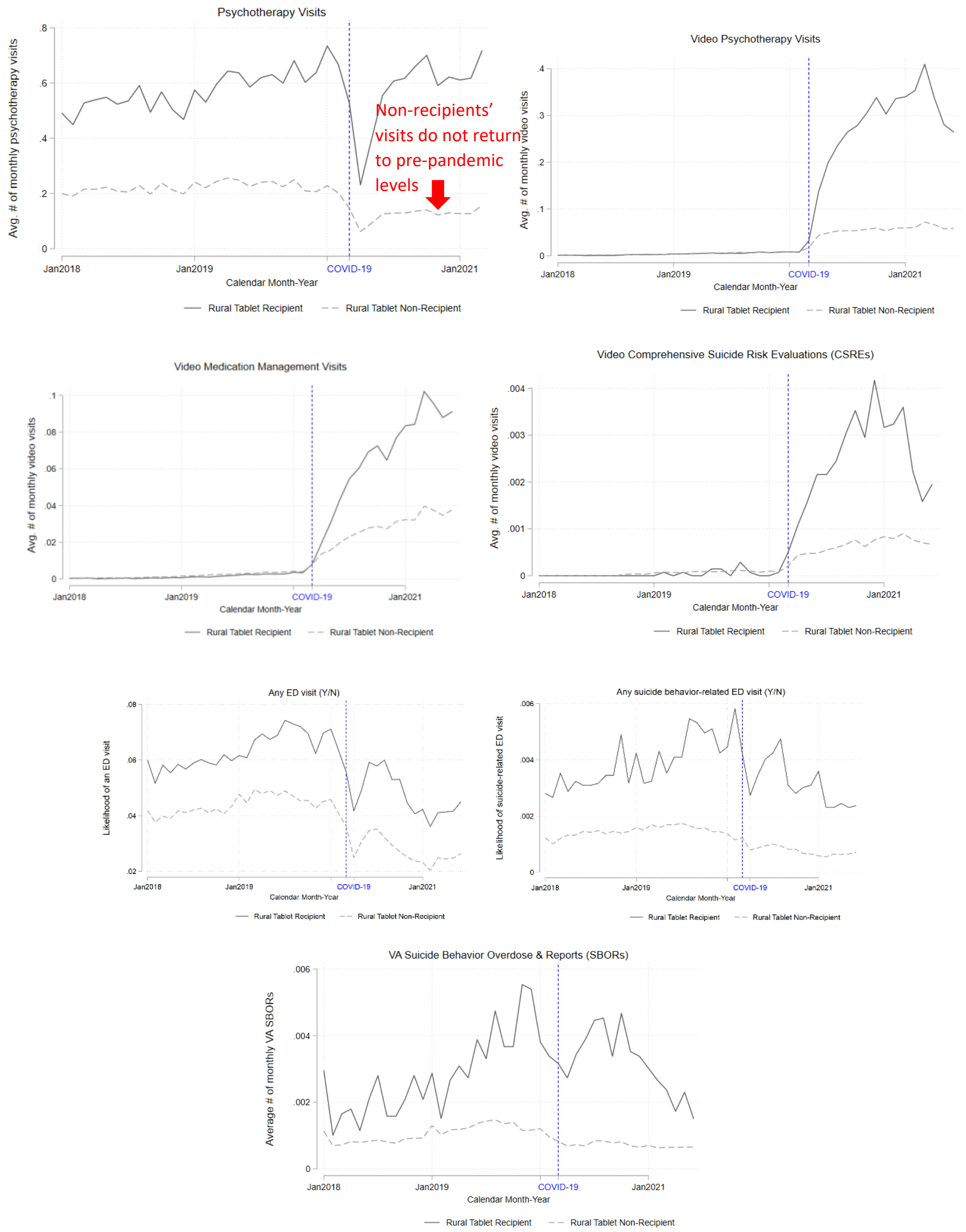

© 2022 Gujral K et al. *JAMA Network Open.*
